# Supplementary material for: Does electronic consent improve the logistics and uptake of HPV vaccination in adolescent girls? A mixed-methods theory informed evaluation of a pilot intervention
Source: BMJ Open. 2020 Nov 3;10(11):e038963. doi: 10.1136/bmjopen-2020-038963 (PMC7640514; doi:10.1136/bmjopen-2020-038963)
Supplement: Supplementary data [file bmjopen-2020-038963supp001.pdf]

Additional File 1: School evaluation forms

DOES ELECTRONIC CONSENT IMPROVE THE LOGISTICS AND UPTAKE OF HPV VACCINATION IN ADOLESCENT GIRLS? A MIXED METHODS THEORY INFORMED EVALUATION OF AN INTERVENTION

The research published in this manuscript was approved by the PHE Research Support & Governance Office (Ref: NR0131) and the London School of Hygiene and Tropical Medicine Observational/Interventions Research Ethics Committee (Ref: 15839).

School evaluation form for e-consent schools

Introduction

The Hounslow and Richmond Community Health Trust is in the process of introducing a system that facilitates the collection of electronic consent for school-aged vaccinations. Your school has been involved in using the e-consent system as part of the HPV vaccination programme and we are keen to learn from this experience. We would be very grateful if you could complete this anonymous evaluation form and return it in the attached envelope to a member of the immunisation team at the end of the immunisation session today.

The information that you share with us will help us to evaluate the usability and acceptability of the e-consent system for school-based immunisations and assess its effectiveness in promoting consent form return and HPV vaccine uptake. The findings will guide decision-making about the future use of the e-consent system and help improve the system.

If you have any questions about this please do not hesitate to contact the research team who are based at the London School of Hygiene & Tropical Medicine.

Contacts:

Dr Tracey Chantler
LSHTM, 15-17 Tavistock Place
London, WC1H 9SH
tracey.chantler@lshtm.ac.uk

Sandra Mounier-Jack
LSHTM, 15-17 Tavistock Place
London, WC1H 9SH
sandra.mounier-jack@lshtm.ac.uk

Evaluation form

Part 1

On a scale of 1-10 (1 being poor, 10 being excellent) please rate the following by circling your number of choice. Please add comment to explain your ratings.

a) The organisation of the immunisation session

1	2	3	4	5	6	7	8	9	10
Poor				Average				Excellent	

Comments:
____
____
____
____

b) The usability of the e-consent system for obtaining consent for adolescent vaccination.

1	2	3	4	5	6	7	8	9	10
Poor				Average				Excellent	

Comments:
____
____
____
____

c) The efficiency of the e-consent system (in terms of reducing administrative burden and improving the organisation of immunisation sessions)

1	2	3	4	5	6	7	8	9	10
Poor				Average				Excellent	

Comments:
____
____
____
____

d) Communication between the immunisation team and the school

1	2	3	4	5	6	7	8	9	10
Poor				Average				Excellent	

Comments:
____
____
____
____

Part 2

a) What role have your school staff played in preparing for the HPV immunisation session?

1. Educational activities (*please tick*): Yes ☐ No ☐

   If yes please state what these were (e.g. assembly):

   ____
   ____
   ____
   ____

2. Providing list of eligible students (*please tick*) Yes ☐ No ☐

3. Send reminders to parents (tick all relevant methods):

   a. Parent Mail ☐
   b. Post reminders to home address ☐
   c. Handing out reminders students ☐
   d. Text messages to parents/guardians ☐
   e. Phone call to parents/guardians ☐
   f. Email to parent/guardians with consent form link ☐

4. How many reminders did you send to non-respondents: No: ______________

5. Have you helped parents/adolescents access computers to be able to fill in the form online?

   Yes ☐ No ☐

b) What is your experience of using or facilitating use of the e-consent system? What do you think about obtaining consent for school-based teenage vaccination electronically?

____
____
____
____

c) What have parents and students said about the e-consent system?

____
____
____
____

d) Which means of obtaining consent do you think is more efficient: paper based or e-consent?

____

____

____

____

____

e) How do you think the system could be improved?

____

____

____

____

____

____

Thank you for completing this evaluation form

School evaluation form for paper-based consent schools

Introduction

The Hounslow and Richmond Community Health Trust is in the process of introducing a system that facilitates the collection of electronic consent for school-aged vaccinations. Your school is likely to use this system in the future hence we are keen to learn from your current experience of being involved in coordinating the HPV vaccination programme for adolescent girls. We would be very grateful if you could complete this anonymous evaluation form and return it in the attached envelope to a member of the immunisation team at the end of the immunisation session today.

The information that you share with us will help us to evaluate the usability and acceptability of the e-consent system for school-based immunisations and assess its effectiveness in promoting consent form return and HPV vaccine uptake. The findings will guide decision-making about the future use of the e-consent system and help improve the system.

If you have any questions about this please do not hesitate to contact the research team who are based at the London School of Hygiene & Tropical Medicine.

Contacts:

Dr Tracey Chantler
LSHTM, 15-17 Tavistock Place
London, WC1H 9SH
tracey.chantler@lshtm.ac.uk
07969404745

Sandra Mounier-Jack
LSHTM, 15-17 Tavistock Place
London, WC1H 9SH
sandra.mounier-jack@lshtm.ac.uk
020 7927 2929

Rosie Cooper
Address to be added
rosie.cooper@phe.gov.uk

Evaluation form

Part 1

On a scale of 1-10 (1 being poor, 10 being excellent) please rate the following by circling your number of choice. Please add comments to explain your ratings.

e) The organisation of the immunisation session

1	2	3	4	5	6	7	8	9	10
Poor				Average				Excellent	

Comments:

____
____
____
____

f) The usability of the current paper-based system for obtaining consent for adolescent vaccination.

1	2	3	4	5	6	7	8	9	10
Poor				Average				Excellent	

Comments:

____
____
____
____

g) The efficiency of the paper-based system (in terms of administrative burden and organisation of immunisation sessions)

1	2	3	4	5	6	7	8	9	10
Poor				Average				Excellent	

Comments:

____
____
____
____

h) Communication between the immunisation team and the school

1	2	3	4	5	6	7	8	9	10
Poor				Average				Excellent	

Comments:

____
____
____
____
____

Part 2

a) What role have your school staff played in preparing for the HPV immunisation session?

1. Educational activities (*please tick*): Yes ☐ No ☐

If yes please state what these were (e.g. assembly):

____
____
____
____

2. Providing list of eligible students (*please tick*) Yes ☐ No ☐

3. Send reminders/consent forms to parents (tick all relevant methods):

   a. Parent Mail ☐

   b. Post reminders/consent forms to home address ☐

   c. Handing out reminders/consent forms to students ☐

   d. Text messages to parents/guardians ☐

   e. Phone call to parents/guardians ☐

   f. Email to parent/guardians with consent form attached ☐

4. How many reminders did you send to non-respondents: No: _______________

b) What is your experience of using or facilitating use of the existing consent system?

____
____
____
____
____

c) What do you think about obtaining consent for school-based teenage vaccination electronically?

____
____
____
____
____

Thank you for completing this evaluation form
