# Supplementary material for: Does electronic consent improve the logistics and uptake of HPV vaccination in adolescent girls? A mixed-methods theory informed evaluation of a pilot intervention
Source: BMJ Open. 2020 Nov 3;10(11):e038963. doi: 10.1136/bmjopen-2020-038963 (PMC7640514; doi:10.1136/bmjopen-2020-038963)
Supplement: Supplementary data [file bmjopen-2020-038963supp002.pdf]

# Additional File 2: Interview Topic Guides

## DOES ELECTRONIC CONSENT IMPROVE THE LOGISTICS AND UPTAKE OF HPV VACCINATION IN ADOLESCENT GIRLS? A MIXED METHODS THEORY INFORMED EVALUATION OF AN INTERVENTION

The research published in this manuscript was approved by the PHE Research Support & Governance Office (Ref: NR0131) and the London School of Hygiene and Tropical Medicine Observational/Interventions Research Ethics Committee (Ref: 15839).

## Interviewees: Immunisation team members

### Experience of obtaining consent in school-based vaccination programmes

- Could you tell me about your experience of obtaining consent from parents and adolescents for school-aged vaccinations?
  - What has worked well and what has been challenging?
  - How have you overcome challenges in the past?

### Acceptability of the e-consent system

- What were your initial thoughts about the e-consent system? (Probes: concerns, hopes, expectations)
- What did your colleagues think about the system?
- How acceptable do you think the system is for parents, teenagers, and school staff involved in the programme?

### Usability of the e-consent system

- How helpful was the training in preparing you to use the e-consent system?
- What was your experience of using the e-consent system? What worked well, what was difficult in the following stages?
  - Triage of consent forms
  - Use of the system during immunisation sessions in schools
  - Uploading data to Trust databases and Child Health Immunisation Systems
  - Any other stages
- What contact did you have with parents about the use of the e-consent system? How did parents find using the e-consent system? What did they find difficult?
- What do you think about how the system may support communication between parents/guardians and adolescents about immunisation? How does this compare with the paper-based system?
- How does the e-consent system support communication between immunisation teams, parents and students? How does this compare with the paper-based system?
- What are the advantages and disadvantaged of the e-consent system in comparison with the paper-based system?

### Interaction between schools and immunisation teams

- What is your experience of working with schools in organising immunisation sessions?
- What were schools reactions to the introduction of the e-consent system?
- How has the e-consent system supported interactions between schools and immunisation teams? (Probes: what has worked well, what has been challenging?) How does this compare with the paper-based system?
- What have schools told you about their experience of using the e-consent system? How do they compare it with the paper-based system?
- Have schools conducted any educational activities in preparation for the HPV immunisation visits? What were these? How have you been involved in these?

### Adolescent self-consent

- What do you think about giving teen-agers the opportunity to self-consent for vaccination? (Probes: age, what vaccines e.g. HPV)
- Have you obtained self-consent for adolescent immunisation? If yes, in what instances, what was the procedure, and how did you assess for competency?
- How does the e-consent system support self-consent? What is the procedure for this? Did any teen-agers use the e-consent system to give consent for HPV vaccination? What was that experience like?
- Have you had conversations with schools about adolescent self-consent? What are their views?
- Have you had conversations with parents/guardians about adolescent self-consent? What were their views?

### Reflections of the use of the e-consent system

- What have been the main lessons in piloting the e-consent system (Probes: what has worked well, what needs to be changed?)
- How would you modify and improve the e-consent system based on the piloting experience?
- What is your advice for other immunisation providers thinking about implementing e-consent?
- Has the e-consent system achieved its intended outcomes from your perspective?

### Any other comments

Do you have any other comments?

**Thank you for your time.**

## Interviewees: Service managers

### Experience of obtaining consent in school-based vaccination programmes

- Could you tell me about your past experience of managing the logistics of obtaining consent from parents and adolescents for school-aged vaccinations?
  - What has worked well and what has been challenging?
  - How have you overcome challenges in the past?

### Decision to launch the e-consent system

- What were the main reasons you decided to implement the e-consent system?
- How did you decide who to collaborate with in developing the e-consent system?
- How was the process of development? What went well, what was difficult?
- How did your test the system prior to the piloting phase?

### Acceptability of the e-consent system

- How did your team initially react to the plans to introduce the e-consent system? (Probes: Expectations, concerns, questions)
- Did opinion change over time, and if yes, how?
- How acceptable do you think the e-consent system is for nurses, parents, teenagers, and school staff involved in the programme?

### Usability of the e-consent system

- How were you involved in using the e-consent system?
- What was your/your teams' experience of using the e-consent system? What worked well, what was difficult in the following stages?
  - Triage of consent forms
  - Use of the system during immunisation sessions in schools
  - Uploading data to Trust databases and Child Health Immunisation Systems
  - Any other stages
- What contact did you/your team have with parents about the use of the e-consent system? How did parents find using the e-consent system? What did they find difficult?
- What do you think about how the system may support communication between parents/guardians and adolescents about immunisation? How does this compare with the paper-based system?
- How does the e-consent system support communication between immunisation teams, parents and students? How does this compare with the paper-based system?
- What are the advantages and disadvantaged of the e-consent system in comparison with the paper-based system?

### Interaction between schools and immunisation teams

- What is your experience of working with schools in organising immunisation sessions?
- What were schools reactions to the introduction of the e-consent system? How did you inform them about the e-consent system, what guidance or training did you provide?
- How has the e-consent system supported interactions between schools and immunisation teams? (Probes: what has worked well, what has been challenging?)
- How does this compare with schools that are using the paper system?
- What have schools told you about their experience of using the e-consent system? How do they compare it with the paper-based system?
- Have schools (those using e-consent and paper system) conducted any educational activities in preparation for the HPV immunisation visits? What were these? How have you/your team been involved in these?

### Adolescent self-consent

- Please describe the process you have used for self-consent
- What do you think about giving teenagers the opportunity to self-consent for vaccination? (Probes: age, what vaccines e.g. HPV)
- Have you had conversations with schools about adolescent self-consent? What are their views?
- Have you had conversations with parents/guardians about adolescent self-consent? What were their views?

### Reflections of the use of the e-consent system

- What have been the main lessons in piloting the e-consent system (Probes: what has worked well, what needs to be changed?)
- How will you modify and improve the e-consent system based on the piloting experience?
- What have been the costs of implementing the e-consent system?
- What is your advice for other immunisation providers thinking about implementing e-consent?
- Has the e-consent system achieved its intended outcomes from your perspective?

### Any other comments

Do you have any other comments?

**Thank you for your time.**

## Interviewees: Service administrators

### Experience of managing the administration of the school-based vaccination programmes

- Could you tell me about your past experience of managing the administration of the school-aged vaccination programme?
    - What has worked well and what has been challenging?
    - How have you overcome challenges in the past?

### Decision to launch the e-consent system (for senior administrators)

- What were the main reasons the Trust decided to implement the e-consent system?
- How was the process of development? What went well, what was difficult?
- How did your test the system prior to the piloting phase?

### Acceptability of the e-consent system

- What were your initial thoughts about the plans to introduce the e-consent system? (Probes: Expectations, concerns, questions)
- Did your opinion change over time, and if yes, how?
- What is your current view on the acceptability of the e-consent system?

### Usability of the e-consent system

- How are you involved in using the e-consent system?
- What was your/your teams' experience of using the e-consent system? What worked well, what was difficult in the following stages?
    - Administration of data from the consent forms
    - Administration of data from school and catch-up immunisation sessions
    - Uploading data to Trust databases and Child Health Immunisation Systems
    - Sharing data with GPs
    - Any other stages
- What are the advantages and disadvantaged of the e-consent system in comparison with the paper-based system?

### Interaction between schools and immunisation teams

- What role do you play in collaborating with schools to organising immunisation sessions?
- What were schools reactions to the introduction of the e-consent system? How were they informed about the e-consent system, what guidance or training did they receive?
- How has the e-consent system supported interactions between schools and immunisation providers? (Probes: what has worked well, what has been challenging?)
- How does this compare with schools that are using the paper system?
- What have schools told you about their experience of using the e-consent system? How do they compare it with the paper-based system?

### Reflections of the use of the e-consent system

- What have been the main lessons in piloting the e-consent system (Probes: what has worked well, what needs to be changed?)
- How will you modify and improve the e-consent system based on the piloting experience?
- What have been the costs of implementing the e-consent system?
- What is your advice for other immunisation providers thinking about implementing e-consent?
- Has the e-consent system achieved its intended outcomes from your perspective?

### Any other comments

Do you have any other comments?

**Thank you for your time.**

## Interviewees: Parents/guardians

### Views on adolescent vaccination

- What do you know about the vaccines that are offered to teenagers? (Probes: HPV, teenage booster, Men ACWY)
- What information have you received about teenage vaccinations? Who gave you this information? Did you search for additional information, if yes where did you look and what did you find?
- What is your view about adolescent vaccination? Are there any vaccines that you are concerned about?

### Experience of providing consent for adolescent vaccinations

- Could you tell me about your experience of providing consent for your teenager(s) to be vaccinated?
- How did you make your decision about providing consent?
  - Who else did you talk to about this? What did you discuss? What were their views? (Probes: Partner, teenager, other family members, friends, health professionals)
- How did you involve your teenager in this decision-making? Were you in agreement about having the vaccine?
- Have you ever changed your mind about providing consent for a vaccination, if yes why and what did you do?

### Usability of the e-consent system

- How did you find using the e-consent system? Who completed the form?
- Was it easy to access the e-consent site via the email sent from school?
- What was you experience of completing the consent form online? Were there any parts that you found difficult to complete? How did you know if you had completed the form properly?
- What was your experience of accessing the HPV vaccination programme leaflets via the e-consent system? Did you read these online or did you print them out? Did you share these leaflets with your teenager or other members of the family?
- Did you contact the immunisation team for help to complete the e-consent form? What support did you require? How easy was it to contact them?
- How do you think that the e-consent form could be improved?

### Acceptability of the e-consent system

- What are your views on giving consent electronically for your teenagers' vaccinations? (Probes: any concerns about sharing personal data online?)
- What did your teen-ager think about this method of giving consent?
- What are your views on how the e-consent system supports information sharing and communication about teenage vaccination?

### Teenagers experience of immunisation in school

- What did your teenager tell you about their experience of being immunised in school? (Probes: what went well, what did they find difficult)
- How well prepared do you think your teenager was prior to the immunisation session? (Probes: access to information, educational sessions in school)

### Views on adolescent self-consent

- What do you think about the option of teen-agers providing self-consent for vaccination?
- What is your understanding of self-consent in adolescence?
- Would you be happy for your teen-ager to give self-consent for vaccination? If yes, why. If no, why?

### Any other comments

Do you have any other thoughts about adolescent vaccination and how consent is obtained for this?

### Thank you for your time.

## Interviewees: Adolescents (questions asked with parents)

### Views on adolescent vaccination

- What do you know about the vaccines that are offered to teenagers? (Probes: HPV, teenage booster, Men ACWY)
- What information have you received about the HPV vaccination? Who gave you this information? Have you accessed any other information, if yes what was this?
- What is your view about the HPV vaccination? (Probes: what is good about it, any concerns about the vaccine or the process of vaccination)
- Who have you talked to about having the HPV vaccine? (Probes: parents, friends, school nurse, other health professionals, other family members) What did you discuss?

### Experience of giving consent for vaccination

- Who decided whether you would receive the HPV vaccine? How did they make this decision?
- How were you involved in this decision-making? Were you in agreement with your parents about this decision?

### Usability of the e-consent system

- Were you involved in using the e-consent system? If yes, how were you involved either at home or in school and what did you think about this system?
- What did your parents think about the e-consent system? Were there any parts of the online form that they found difficult to complete?
- Were you given the opportunity to find out more about the HPV vaccine via the e-consent system? (Probes: Access leaflets, read and discuss these with your parents)
- What do your friends think about the e-consent system?
- How do you think that the e-consent system could be improved?

### Acceptability of the e-consent system

- What are your views on giving consent online for your vaccinations? (Probes: any concerns about sharing personal data?)
- What are your views on how the e-consent system supports finding out about and talking about teenage vaccination?

### Experience of the immunisation session

- Could you tell me a bit about your experience of receiving the HPV vaccine in school? What was the session like, how did you feel, what went well, what was difficult?
- How well prepared were you for the immunisation session? (Probe: know you would be vaccinated against HPV, understood what the vaccine was for any why you needed it)
- Were you given the opportunity to ask any questions before you were vaccinated?
- Had you participated in any information sessions about vaccination at school before you were vaccinated? If yes, what were these and what did you learn?
- Had you received any other information about the HPV vaccine before you were vaccinated? If yes, who gave you this?

### Views on adolescent self-consent

- What do you think about the option of teen-agers providing self-consent for vaccination?
- What is your understanding of self-consent?
- Have you ever been given the option to provide self-consent for vaccination?
- Would you be happy to provide self-consent for vaccination? If yes, why. If no, why?

### Any other comments

Do you have any other thoughts about vaccination and how consent is obtained for this?

**Thank you for your time**

## Adolescent Focus Group Discussion Topic Guide

**Interviewees:** Adolescents (Year 8/9 girls)

**Views on adolescent vaccination**

- What do you know about the vaccines that are offered to teenagers? (Probes: HPV, teenage booster, Men ACWY)
- What information have you received about the HPV vaccination? Who gave you this information? Have you accessed any other information, if yes what was this?
- What is your view about the HPV vaccination? (Probes: what is good about it, any concerns about the vaccine or the process of vaccination)
- Who have you talked to about having the HPV vaccine? (Probes: parents, friends, school nurse, other health professionals, other family members) What did you discuss?

**Experience of giving consent for vaccination**

- Who decided whether you would receive the HPV vaccine?
- How were you involved in this decision-making? Were you in agreement with your parents about this decision?

**Usability of the e-consent system**

- Were you involved in using the e-consent system? If yes, how were you involved either at home or in school and what did you think about this system?
- What did your parents think about the e-consent system? Were there any parts of the online form that they found difficult to complete?
- Were you given the opportunity to find out more about the HPV vaccine via the e-consent system? (Probes: Access leaflets, read and discuss these with your parents)
- What do your friends think about the e-consent system?
- How do you think that the e-consent system could be improved?

**Acceptability of the e-consent system**

- What are your views on giving consent online for your vaccinations? (Probes: any concerns about sharing personal data?)
- What did your parents think about this way of obtaining consent?

**Experience of the immunisation session**

- Could you tell me a bit about your experience of receiving the HPV vaccine in school? What was the session like, how did you feel, what went well, what was difficult?
- How well prepared were you for the immunisation session? (Probe: know you would be vaccinated against HPV, understood what the vaccine was for any why you needed it)
- Were you given the opportunity to ask any questions before you were vaccinated?

- Had you participated in any information sessions about vaccination at school before you were vaccinated? If yes, what were these and what did you learn?
- Had you received any other information about the HPV vaccine before you were vaccinated? If yes, who gave you this?

### Views on adolescent self-consent

- What do you know about the option of teenagers providing self-consent for vaccination?
- Do you know what self-consent means?
- Would you be happy to provide self-consent for vaccination? If yes, why. If no, why?

### Any other comments

Do you have any other thoughts about vaccination and how consent is obtained for this?

**Thank you for your time.**
