# Supplementary material for: Does electronic consent improve the logistics and uptake of HPV vaccination in adolescent girls? A mixed-methods theory informed evaluation of a pilot intervention
Source: BMJ Open. 2020 Nov 3;10(11):e038963. doi: 10.1136/bmjopen-2020-038963 (PMC7640514; doi:10.1136/bmjopen-2020-038963)
Supplement: Author's manuscript [file bmjopen-2020-038963.draft_revisions.pdf]

BMJ Open is committed to open peer review. As part of this commitment we make the peer review history of every article we publish publicly available.

When an article is published we post the peer reviewers' comments and the authors' responses online. We also post the versions of the paper that were used during peer review. These are the versions that the peer review comments apply to.

The versions of the paper that follow are the versions that were submitted during the peer review process. They are not the versions of record or the final published versions. They should not be cited or distributed as the published version of this manuscript.

BMJ Open is an open access journal and the full, final, typeset and author-corrected version of record of the manuscript is available on our site with no access controls, subscription charges or pay-per-view fees (http://bmjopen.bmj.com).

If you have any questions on BMJ Open's open peer review process please email info.bmjopen@bmj.com

BMJ Open

DOES ELECTRONIC CONSENT IMPROVE THE LOGISTICS AND UPTAKE OF HPV VACCINATION IN ADOLESCENT GIRLS? A MIXED METHODS THEORY INFORMED EVALUATION OF AN INTERVENTION

Journal:	*BMJ Open*
Manuscript ID	bmjopen-2020-038963
Article Type:	Original research
Date Submitted by the Author:	30-Mar-2020
Complete List of Authors:	Chantler, Tracey; London School of Hygiene & Tropical Medicine, Global Health & Development<br>Pringle, Ellen; Public Health England, Department of Immunisation, Hepatitis and Blood Safety, Public Health England, UK<br>Bell, Sadie; London School of Hygiene and Tropical Medicine Faculty of Public Health and Policy, Global Health and Development<br>Cooper, Rosie; University Hospitals of Derby and Burton NHS Foundation Trust<br>Edmundson, Emily; Hounslow and Richmond Community Healthcare NHS Trust<br>Nielsen, Heidi; Hounslow and Richmond Community Healthcare NHS Trust<br>Roberts, Sheila; Hounslow and Richmond Community Healthcare NHS Trust<br>Edelstein, Michael; Public Health England, Immunisation and countermeasures division<br>Mounier-Jack, Sandra; London School of Hygiene and Tropical Medicine,
Keywords:	Public health < INFECTIOUS DISEASES, Information technology < BIOTECHNOLOGY & BIOINFORMATICS, Paediatric infectious disease & immunisation < PAEDIATRICS

SCHOLARONE™
Manuscripts

I, the Submitting Author has the right to grant and does grant on behalf of all authors of the Work (as defined in the below author licence), an exclusive licence and/or a non-exclusive licence for contributions from authors who are: i) UK Crown employees; ii) where BMJ has agreed a CC-BY licence shall apply, and/or iii) in accordance with the terms applicable for US Federal Government officers or employees acting as part of their official duties; on a worldwide, perpetual, irrevocable, royalty-free basis to BMJ Publishing Group Ltd ("BMJ") its licensees and where the relevant Journal is co-owned by BMJ to the co-owners of the Journal, to publish the Work in this journal and any other BMJ products and to exploit all rights, as set out in our licence.

The Submitting Author accepts and understands that any supply made under these terms is made by BMJ to the Submitting Author unless you are acting as an employee on behalf of your employer or a postgraduate student of an affiliated institution which is paying any applicable article publishing charge ("APC") for Open Access articles. Where the Submitting Author wishes to make the Work available on an Open Access basis (and intends to pay the relevant APC), the terms of reuse of such Open Access shall be governed by a Creative Commons licence – details of these licences and which Creative Commons licence will apply to this Work are set out in our licence referred to above.

Other than as permitted in any relevant BMJ Author's Self Archiving Policies, I confirm this Work has not been accepted for publication elsewhere, is not being considered for publication elsewhere and does not duplicate material already published. I confirm all authors consent to publication of this Work and authorise the granting of this licence.

For peer review only

DOES ELECTRONIC CONSENT IMPROVE THE LOGISTICS AND UPTAKE OF HPV VACCINATION IN ADOLESCENT GIRLS? A MIXED METHODS THEORY INFORMED EVALUATION OF AN INTERVENTION

Authors

Tracey Chantler[1]* §, Ellen Pringle*[2]

Sadie Bell[1], Rosie Cooper[3], Emily Edmundson[4], Heidi Nielsen[4], Sheila Roberts[4], Michael Edelstein[1,2], Sandra Mounier Jack[1]

1. Department of Global Health and Development, Faculty of Public Health and Policy, London School of Hygiene & Tropical Medicine, 15-17 Tavistock Place, London, WC1H 9SH, UK
2. Department of Immunisation, Hepatitis and Blood Safety, Public Health England, UK
3. University Hospitals of Derby and Burton NHS Foundation Trust, Uttoxeter Road, Derby, DE22 3NE
4. Hounslow Richmond Community Healthcare NHS Trust, 18 High Street, Teddington, London, TW118HU, UK

*Joint first authors

§Corresponding author: Tracey Chantler, Department of Global Health and Development, Faculty of Public Health and Policy, London School of Hygiene & Tropical Medicine, 15-17 Tavistock Place, London, WC1H 9SH, UK, tracey.chantler@lshtm.ac.uk Tel: 07969404745

Word count: 3984

For peer review only

For peer review only - http://bmjopen.bmj.com/site/about/guidelines.xhtml

Abstract

Objectives

To evaluate the usability and acceptability of an electronic consent intervention for school-based immunisations and assess its impact on consent form returns and HPV vaccine uptake.

Design

This was a mixed-methods theory-informed study which used quantitative methods to assess the impact of the intervention and qualitative methods to examine its usability and acceptability.

Setting and participants

The intervention was implemented in 14 secondary schools in seven London boroughs. Intervention schools were matched with schools using paper consent based on the proportion of students with English as a second language and students receiving free school meals. Participants included nurses, data managers, school link staff, parents and adolescents.

Interventions

An electronic consent portal where parents could agree to or decline vaccination and nurses could access data to help them manage the immunisation programme.

Primary and Secondary Outcome Measures

Comparison of consent form return rates and HPV vaccine uptake between intervention and matched schools.

Results

HPV vaccination uptake did not differ between intervention and matched schools, but timely consent form return was significantly lower intervention schools (73.3% (n=11) vs 91.6% (n=11), p=0.008). The transition to using electronic consent was not straightforward, whilst schools and staff understood the potential benefits, they found it difficult to adapt to new ways of working which removed some level of control from schools. Reasons for lower consent form return in e-consent

For peer review only - http://bmjopen.bmj.com/site/about/guidelines.xhtml

schools included difficulties encountered by some parents in accessing and using the intervention. Adolescents highlighted the potential for electronic consent to by-pass their information needs.

Conclusions:

The intervention did not improve consent form return or vaccine uptake due to challenges encountered in transitioning to a new way of working. New technologies require embedding before they become incorporated in everyday practice. A re-evaluation once stakeholders are accustomed with electronic consent may be required to understand its impact.

Keywords

Public health, Information technology, Paediatric infectious disease and immunisation

Strengths and limitations

- The use of a theory-informed mixed methods study design allowed us to measure the effect of an e-consent intervention on immunisation performance and identify mechanisms that facilitated or impeded implementation.
- The study design allowed us to account for schools, nurses, data managers, parents and adolescents' experiences of using the e-consent technology in this evaluation.
- Data limitations include the lack of interviews with school staff to complement the feedback forms and not being able to access and interview parents who found the intervention more challenging to use.
- This evaluation was limited to one cycle of implementation which did not allow us to account for the time it takes for new practices to become embedded.

For peer review only - http://bmjopen.bmj.com/site/about/guidelines.xhtml

For peer review only

Introduction

Vaccination against human papilloma virus (HPV) provides long-term protection against cervical and other cancers and genital warts[1, 2]. In England HPV vaccination (2 doses 6 months apart) is offered to adolescents aged 12-14 years in the school-based immunisation programme. Whilst this delivery model is widely accepted[3], it raises logistical challenges regarding obtaining parental consent. Students are typically given a paper consent form that their parents/legal guardians need to sign. The passage of the form from teachers to students to parents and back again can impact on form return and have a detrimental effect on vaccine uptake[4-7]. Follow-up by school staff and immunisation teams improves uptake but is resource intensive[8, 9]. In the context of the drive towards a "paperless NHS"[10], there is increasing interest in the potential for technological solutions, such as electronic consent (e-consent). We evaluated the usability and acceptability of an e-consent intervention from the perspective of parents and adolescents, health professionals and schools, and assessed its impact on consent form returns and HPV vaccine uptake.

The electronic consent intervention

The e-consent intervention was developed by Hounslow and Richmond Community Healthcare NHS Trust (HRCH) with the support of a software development company in 2017/18 and piloted in their adolescent girls' vaccination programme in June/July 2018. At the time of the study HRCH was responsible for administering this vaccination programme in secondary schools across eight boroughs in South London.

The e-consent intervention comprised an online portal with an e-consent form and a data platform and related implementation procedures (Figure 1). Functionally it aimed to: 1) give parents access to an online portal with information about the vaccination programme where they could register

For peer review only - http://bmjopen.bmj.com/site/about/guidelines.xhtml

their adolescent and agree to or decline HPV vaccination; 2) give nurses electronic access to the portal to facilitate screening and enable them to update records during immunisation sessions; 3) enable automatic updating of central vaccination record databases. Parts of the online portal and data platform (specifically those relating to points 2 & 3) were not fully functioning before the intervention was first used in June 2018. Hence modifications had to be made to the way nurses screened students' information and consent forms before and during immunisation sessions. Figure 1 differentiates what happened in Year 1: June/July 2018 and in Year 2: June/July 2019 (blue text). In this evaluation we focused primarily on Year 1, especially in the quantitative measurement of outcomes and impacts, however we were also able to capture how Year 1 experience informed adaptations to the intervention before it was reused in Year 2.

Methods

Study design

This was a mixed-methods theory-informed study which used a 'Theory of Change' (Figure 2) as an evaluation framework. Quantitative methods were used to assess whether the intervention increased consent form return and the uptake of the first dose of HPV vaccine in adolescent girls in June/July 2018. The steps involved in implementing the e-consent system, people's experiences of these, the interactions between inputs, activities, pathways and outputs, outcomes and impacts were investigated using qualitative methods between June 2018-July 2019.

School selection

The e-consent intervention was used in 14 secondary schools in seven South London boroughs in June/July 2018. Schools selection was purposive with the aim of including schools that differed in terms of denomination (private, state, grammar), type (mixed, single sex), socio demographic, size, vaccination uptake, and level of support to the programme.

For peer review only - http://bmjopen.bmj.com/site/about/guidelines.xhtml

Schools were divided into low, medium or high based on the proportion of pupils receiving free school meals and with English as an additional language. Each e-consent school was matched, as closely as possible, to a paper consent school in the same tertile for both characteristics.

Quantitative methods & analysis

Data collection

At each vaccination session nurses completed a "tally sheet", with details of the consents received prior to or during the session, any absences and the number of vaccinations given. These tally sheets were completed for paper and e-consent schools.

In addition, we extracted the name of the school, date and time of consent form completion and type of consent (agreement by parent or adolescent self-consent or decline) for each consent form in the e-consent system. This non-identifiable information was combined with tally sheet data, which was manually transcribed into MS Excel. Where there were discrepancies or missing data this was checked with the immunisation teams and in the case of the e-consent schools the system data was used in preference to the tally sheet data.

Data analysis

Characteristics of the paper and e-consent schools were described in terms of proportion of pupils receiving free school meals, speaking English as an additional language, ethnicity and characteristics of the schools (religious affiliation and state/privately funded), using data from the Office of National Statistics. (REF: https://www.gov.uk/government/statistics/schools-pupils-and-their-characteristics-january-2018).

For both paper and e-consent schools, we calculated: (i) the proportion of the pupils for whom a consent form had not been returned prior to the vaccination session, (ii) the proportion of pupils vaccinated at the planned session and (iii) the proportion of pupils for who a "yes" consent was received (prior to or on the day of the planned vaccination session).

For peer review only - http://bmjopen.bmj.com/site/about/guidelines.xhtml

We compared e-consent schools with paper consent schools in terms of timely form return, outcome of consent and vaccination uptake using Kruskall Wallis tests for statistical significance. Where data was missing from a school that school and its matched pair were excluded from the analysis. All analyses were done in MS Excel and Stata 15.

Qualitative methods & analysis

Data collection

Data collection occurred in June-December 2018 and June-July 2019. The first period coincided with and followed the first year of e-consent implementation, the second occurred during the use of e-consent in a subset of schools.

June-July 2018

Observations of e-consent school HPV immunisation sessions

Members of the evaluation team (RC, TC, SMJ) accompanied immunisation teams during seven e-consent school HPV immunisation sessions to obtain a contextual understanding of the implementation of the intervention. The evaluators documented what worked well and what if any problem-solving was applied during sessions.

School feedback forms from paper and e-consent schools

School staff involved in organising immunisation sessions were asked to complete a feedback form after the HPV immunisation sessions (Additional file 1). Forms included questions about the organisation of immunisation sessions and the usability and acceptability of e-consent and paper consent. Seven e-consent and seven paper consent schools completed forms.

Individual and peer group interviews with HRCH staff

These semi-structured interviews (SSI's) involved HRCH staff who were responsible for implementing the intervention. The programme manager was interviewed individually, the two data specialists

For peer review only - http://bmjopen.bmj.com/site/about/guidelines.xhtml

together and member of four immunisation teams in peer-groups of two to four participants (12 interviewees) at their respective offices. In total 15 members of staff took part in hour long interviews.

Interviews with parents and adolescents from e-consent schools

These SSIs involved parents and adolescents who had used the e-consent system. Parents/legal guardians were asked to indicate their willingness to be contacted by researchers in the e-consent form (ticked a statement). Parents who responded positively were invited to take part in an interview to discuss their experience of using the e-consent system. A total of 12 interviews were conducted, nine with HPV vaccine acceptors and three with decliners. Four interviews were conducted in family homes, seven by telephone, and one by Skype video. Adolescents participated in five interviews.

June-July 2019

Results from data collected in 2018 informed the implementation of the e-consent intervention in 22 schools in the 2018/19 HPV vaccine programme. To reflect on these changes two group interviews with HRCH staff (one with nine and the other with five members of staff) and one focus group discussion (FGD) with eight adolescent girls (who had recently received an HPV vaccine at an e-consent school) were conducted.

The topics covered in the staff, parent and adolescent SSIs and adolescent FGD are summarised in Text Box 1 (See Additional File 2 for Topic Guides).

For peer review only - http://bmjopen.bmj.com/site/about/guidelines.xhtml

Text box 1: Topics covered in interview

Staff interviews	Parent and adolescent interviews
• Experience of managing the administration of the school-based vaccination programme<br>• Experience of obtaining consent in school-based vaccination programme<br>• Acceptability and usability of the e-consent system<br>• Interaction between schools and immunisation teams<br>• Adolescent self-consent<br>• Reflections on use of the e-consent system	• Views on adolescent vaccination<br>• Understanding of adolescent vaccination<br>• Experience of providing consent for the HPV vaccine<br>• Acceptability and usability of the e-consent system<br>• Teenagers experience of immunisation in school<br>• Vaccine programme communication<br>• Views on adolescent self-consent

Data analysis

The SSIs and FGD were audio recorded with participant's permission and transcribed anonymously. Transcripts, observational field notes and school feedback forms were downloaded into a qualitative data analysis management software programme (NVivo 12). We adopted a thematic analytical approach which combined semi-deductive mapping of data to the 'input', 'activities', 'pathways', 'outputs', 'outcomes' and 'impacts' depicted in the ToC and inductive open coding to capture emerging themes[11]. We sought to account for the interdependence of ToC categories and real-life experience of managing organisational change, which do not always progress from inputs – impacts in a seamless linear manner.

Patient and public involvement

This evaluation was designed with members of HRCH who were responsible for coordinating the implementation of the e-consent intervention. This collaboration supported the iterative and reflexive development of the intervention which will be essential its longer-term integration.

For peer review only - http://bmjopen.bmj.com/site/about/guidelines.xhtml

Results

Quantitative

Participants

Twenty-eight schools (14 paper and 14 e-consent schools) comprising 3219 girls (1733 in paper consent and 1486 in e-consent schools) were included in the study. Of those schools, 26 were state and 2 were private schools. Twenty-one of the schools had no religious affiliation, three were Roman Catholic (all paper consent), three Church of England (two e-consent, one paper consent) and one was another Christian faith school (paper consent).

The proportion of pupils eligible for free school meals, with English as an additional language and students' ethnicity profile was similar between the e-consent and paper consent schools (Table 1).

Table 1: Characteristics of paper and e-consent schools[1,2]

	Paper consent schools (n=13)	E-consent schools (n=13)	P-value*
**% (range) of children eligible for free school meals**	17.3 (1.5-52.1)	17.3 (1.5-43.2)	0.84
**% (range) of children with English as additional language**	30.9 (5.5-53.6)	32.4 (9.1-59.2)	0.72
**% (range) of children of white British ethnicity**	30.9 (1.5-69.6)	34.6 (6.3-65.7)	0.63

*Refers to comparison of each characteristic using Kruskal-Wallis test

1. Characteristics information was not available for the two private schools
2. Data from Schools, pupils and their characteristics: January 2018, Department of Education

For peer review only

For peer review only - http://bmjopen.bmj.com/site/about/guidelines.xhtml

Return of consent forms ahead of session

Overall 83% of consent forms (paper or e-consent) were returned prior to the vaccination session. However, compared with paper schools timely (prior to the planned session) return was lower in the e-consent schools (73.3% (n=11) vs 91.6% (n=11), p=0.008). We could only measure this difference in 22 matched schools.

Outcome of consent

There was no statistically significant difference in the proportion of pupils for whom a "yes" consent was received (prior to or on the day of the session) between the paper (n=14) and e-consent (n=14) schools (85% in e-consent schools, 83% in paper consent schools, p=0.89).

Vaccination uptake

There was no statistically significant difference in the proportion of pupils that were vaccinated at the scheduled vaccination session between the paper (n=14) and e-consent (n=14) schools (80.6% vs 81.3%, p=0.93). These figures did not include those who were absent on the day and vaccinated later. The final vaccine uptake across all the schools was over 86%.

Qualitative

The results of the ToC thematic analytical mapping are presented under relevant headings ('input', 'activities', 'pathways', 'outputs', 'outcomes') followed by an overarching theme on managing change.

Inputs

Resources and Training

There was a *'buzz'* about the development of the e-consent intervention, its potential to streamline consent and facilitate safer data collection and NHS England had provided funding for HRCH to pilot it during the 2017/18 HPV vaccine programme. Immunisation team members were positive but

For peer review only - http://bmjopen.bmj.com/site/about/guidelines.xhtml

expressed reservations about not able to review paper consent forms prior to immunisation. Due to tight deadlines only one orientation session took place before the e-consent intervention was introduced, which meant that the bulk of learning happened on the job.

"I think as well, it was probably four days before our first session, we didn't know what we were doing...so I do feel we are running before we can walk." (Immunisation Team 2)

Intervention not fully operational

The data platform component of the online portal was not operational prior to implementation. Parents were able to access and complete the e-consent form, but the immunisation teams could not review student's online consent forms or upload data during immunisation sessions. Instead large (A3) paper sheets with information about who had provided consent were prepared by the data managers. The sheets were difficult for to decipher during busy sessions and nurses were less able to prepare cohort figures and tally sheets in advance.

"It was an anti-climax not being able to use the laptops and still have a paper sheet in front of me." (Immunisation Team 4)

Mobilizing and resourcing schools

There was limited time to collaborate with schools before the vaccination programme, although all e-consent schools were guided on how to disseminate the weblink. A few schools declined to use the intervention due to concerns about barriers to electronic communication with parents.

School immunisation link staff reported a *'loss of control'*, for example they could no longer see *'who had said yes, and who had said no'*, which restricted their ability to follow-up unreturned forms. Paper-consent schools could monitor this directly by counting forms, but with e-consent schools immunisation teams had to check parental responses and tell schools which families had not replied.

For peer review only

For peer review only - http://bmjopen.bmj.com/site/about/guidelines.xhtml

Activities

Dissemination of the online e-consent portal weblink to parents

E-consent schools used different means (parent mail, email, school website, newsletters, letters) to send parents the portal weblink. Blanket reminders were mainly sent electronically, unless immunisation teams provided schools with details of non-responders. In this case follow-up could be more targeted and involve text messages and phone calls as wells as emails. One school used a translator to engage parents who did not understand the consent process due to language barriers. Another school was not willing to send out emails and asked the immunisation team to provide them with printed letters referring to the weblink to send to parents.

Of the seven e-consent schools who completed the feedback form, four were positive about the intervention and how it had been implemented stating that it had reduced their workload. Another school was mainly positive but noted that some parents had found the e-consent form difficult to access, another reported that their parent cohort had found the system very difficult to access and use, and the last school was the one who had used letters to disseminate the weblink.

Pathways

Navigating the e-consent form and related information

The e-consent form included links to an HPV vaccination leaflet. However, none of the interviewees had downloaded or read this leaflet for the following reasons: accessed information elsewhere, already sufficiently informed, older daughter vaccinated, positive about vaccination.

Proactive information seeking was more common in families who were vaccine hesitant. Parents who were more confident about vaccines restricted their information seeking to NHS sources and suggested that a 'road map' to adolescent vaccination could be useful.

For peer review only - http://bmjopen.bmj.com/site/about/guidelines.xhtml

Adolescents reported a variety of information seeking behaviours. Some just accepted HPV vaccination as '*something that needs to be done*' and felt reassured that it was recommended by the NHS: *"I think because it's like by the NHS - it kind of gives it validation." (Adolescent 9 - Yes)*. Others wanted the HPV vaccine leaflet to include more information about HPV and related health risks and vaccine side-effects, so that they did not panic if they experienced any of these.

In the FGD students expressed a preference for paper leaflets and discussed how the e-consent could bypass them: *"…because like if it's emailed, like your mum doesn't have to share it with you. And like if I have something done like an injection, I'd like to know what's going on and when. But like she filled out the form without like telling me, so like if they'd been given out in school then I could have read it and see what's happening."*

Using the e-consent intervention

The parents we interviewed found the system easy to use and usually completed the form as soon as they received it. A few parents would have liked an email confirmation after they had completed the e-consent form.

"I thought it was very easy. I think you're probably going to get more responses that way from parents in this day and age. However, the downside is obviously you may not get that chance to discuss it. (Parent 5 – Yes)

According to feedback from nurses and schools not all parents found the intervention easy to access or use. Language barriers accounted for some difficulties, but practical issues also played a role, for example some parents had not signed up for the school parent mail system hence did not receive the weblink. Nurses also received a significant number of calls from parents reporting that the weblink would not open or that webpages froze. During an immunisation session one student stated: *"my dad said I should have the vaccine, but he did not understand the whole google business about it".* In some instances, the weblink closed a few days prior to an immunisation session to give immunisation teams time to screen student information prior to sessions. This resulted in some

For peer review only - http://bmjopen.bmj.com/site/about/guidelines.xhtml

parents who had missed the last sign-up date sending in written notes to confirm their consent/non-consent for vaccination.

Outputs

Nurses access to e-consent forms and student information

The immunisation sessions at e-consent schools were affected by nurses not being able to access electronic information about students before and during sessions. The lower return of consent forms in e-consent schools also resulted in nurses reporting that they had to contact more parents than usual during immunisation sessions to obtain verbal consent. This had implications for the nurses' workload distribution and the length of sessions. To manage these challenges immunisation teams increased the number of nurses and administrative assistants who attended e-consent school immunisation sessions.

"…we had 80 consent forms outstanding at a big school. But, normally, if you only have a couple it's fine. It meant us was making calls all morning, it took a nurse out of immunising to be able to do that, so that did have a big impact." (Immunisation Team 2)

Conducting phone calls during sessions was not straightforward. Firstly, nurses had to rely on students (if they had phones with them) or staff to help them access correct contact details. Secondly, immunisations sessions were busy and noisy which impeded communication and privacy. Thirdly, it was not always possible to reach parents who were at work or out of the house during day-time hours. If parents were uncontactable the nurses assessed if students who wanted to be vaccinated had sufficient maturity and intelligence to understand and appraise the nature and implications of the proposed vaccination[12, 13]. This process was time-consuming and not all nurses felt comfortable about vaccinating without verbal or written parent permission.

For peer review only - http://bmjopen.bmj.com/site/about/guidelines.xhtml

Transition: adapting to change and iterative development

The initial 'buzz' about the e-consent intervention decreased over time. Whilst some staff remained positive and receptive to the implementation coordinators enthusiasm and vision, others expressed a sense of half-heartedness about having to adapt quickly from a known way of obtaining consent, albeit with flaws (e.g. cost of paper, mileage clocked up in collection paper consent forms from schools), to a new technology enabled way with some functional limitations in Year 1 (see Figure 1).

In the HRCH staff group interviews conducted in July 2019 members of the immunisation teams reflected on lessons learnt from their experience of transitioning to a *'brand new way of working'* over the past year. Key learning points from an internal organisational perspective were: (i) adopt right pace of progress when introducing new interventions with have several components, (ii) be clear about which part of a multi-component intervention is being piloted and implemented (e.g. in Year 1 it was primarily about the e-consent form), (iii) importance of timely communication, quick thinking, and flexibility when things do not quite go to plan.

In terms of school engagement HRCH staff emphasised the importance of close collaboration to devise appropriate means of consent in different educational and social contexts. The right balance of responsibility between schools and immunisation teams needs to be negotiated to maintain positive working relationships and ensure that adolescents can access essential vaccines.

"I would also say the idea of just changing to e-consent… schools need different things… it is really important to work with the school and a make sure that they are happy with everything and it suits that school, because some schools it might just not suit right now. It might suit them in a couple of years, but right now it just doesn't work." (HRCH immunisers group interview, July 2019)

HRCH made several changes to the implementation of the e-consent intervention in Year 2 (2018/19) informed by evaluation findings (Text Box 2).

For peer review only - http://bmjopen.bmj.com/site/about/guidelines.xhtml

Text Box 2:
E-consent intervention implementation changes between Yr1 and Yr2

- Taking more time to engage (emails, phone calls and meetings) with schools in preparations to find the right level of involvement
- Ensuring students receive a paper copy of the HPV adolescent programme leaflet produced by Public Health England in addition to information provided in the e-consent form
- Pushing more for assemblies and contact with adolescent girls prior to the immunisation sessions
- Providing ongoing training and mentoring of immunisation teams on use of the intervention

Discussion

The e-consent intervention had no detected impact on vaccine uptake and did not improve the return rate of consent forms in its first year of implementation. This could be interpreted as a negative result, however this would fail to account for the embedding process required before new technologies become (or do not become) routinely incorporated into everyday practice[14], and inherent difficulties in achieving high vaccine uptake in urban centres. Our findings demonstrate that the transition from paper to e-consent was not straightforward, and that staff and schools, though mainly open to change, took time to adapt. The transition was hampered by the reduced functionality of the IT capability (intervention fidelity), limited staff training and engagement of schools. It is likely that this e-consent intervention would need to be used for more than one year to achieve any benefits to process efficiency and vaccine uptake.

This study addresses an evidence gap and demonstrates shows how real-time evaluation can support the iterative development of interventions essential for their longer-term integration. Key limitations include the lack of interviews with school staff to complement the feedback forms and not being able to access and interview parents who found the intervention more challenging to use.

For peer review only - http://bmjopen.bmj.com/site/about/guidelines.xhtml

The ‘Normalisation Process Theory’ (NPT) argues that new practices become routinely embedded as the result of people working, individually and collectively, to enact them. Enactment is promoted or inhibited through the operation of four mechanisms; coherence (sense-making), cognitive participation (individual buy-in), collective action (joint effort) and reflexive monitoring (user appraisal)[15]. Sense-making requires users to have a shared understanding concerning the purpose of an intervention, their responsibilities in its implementation, its potential benefits and the differences to existing practice[16]. In our study, immunisation teams recognised the potential value of the intervention but were not clear about their specific responsibilities and schools had to make sense of the change in their role in consent logistics. User buy-in is defined as user’s agreement to try out a new way of completing a task and their willingness to drive and sustain the implementation of a new intervention[15]. HRCH coordinators encouragement, ability to adapt when problems arose was critical to sustaining momentum throughout the implementation phase. Most schools bought into the concept of technology-enabled consent yet their willingness to sustain this change could have benefited from earlier collaboration between immunisation teams, head teachers, and link staff. Parents and student’s engagement with the e-consent intervention was variable. Parent interviewees accessed the online portal and were able to complete and submit the e-consent form with ease. However, the significant difference in consent form return rate between e-consent and paper consent schools (73.3% (n=11) vs 91.6% (n=11), p=0.008) response rate (73.3%) indicated that this was not the case for all parents. Of additional concern was that the e-consent intervention inadvertently bypassed some adolescents’ information needs and related opportunity to talk to their parents about HPV vaccination. Collective action was evidenced when immunisation teams and schools worked together to make the follow-up of e-consent form non-response more targeted. Reflexive monitoring was facilitated by this real-time evaluation although more co-production activities with HRCH staff, parents, school link staff and adolescents need to be integrated in subsequent evaluations[17, 18].

For peer review only

For peer review only - http://bmjopen.bmj.com/site/about/guidelines.xhtml

There is a need to streamline consent processes for adolescent immunisation and ensure that parents and adolescents are fully informed about these preventative measures. Electronic consent could be an effective option to achieve this. However, it needs to be tailored to specific contexts, and parents, schools and adolescents need to provide input in the development, implementation and evaluation of such technological interventions. Evaluations also need to factor in the time needed for new working practices to be fully integrated. Our experience suggests that to gain a complete and accurate assessment of the impact of new interventions evaluations need to collect data over more than one cycle of implementation. Introducing change that affects different actors requires all stakeholders to understand, buy in and work together in refining and co-producing complex behavioural interventions.

Acknowledgements

We are grateful for the information shared freely by interview participants, schools and the Hounslow and Richmond Community Healthcare NHS Trust.

Author Contributors

TC and SMJ designed the study with input from SR, ET, HN, ME and RC. EP led the quantitative data analysis with support from RC and SB. TC collected and led the analysis of the qualitative data with support from SB. All authors discussed the preliminary findings and contributed to and critically reviewed the manuscript.

Funding

The research was funded by the National Institute for Health Research Health Protection Research Unit (NIHR HPRU) in Immunisation (Grant reference: HPRU-2012-10096) at the London School of Hygiene and Tropical Medicine in partnership with Public Health England (PHE). The views expressed are those of the author(s) and not necessarily those of the NHS, the NIHR, the Department of Health or Public Health England.

For peer review only

For peer review only - http://bmjopen.bmj.com/site/about/guidelines.xhtml

Competing interests

Tracey Chantler, Sadie Bell, and Sandra Mounier-Jack report that they were in receipt of funding from the National Institute of Health Research while conducting this research. Michael Edelstein, Rosie Cooper and Ellen Pringle worked for Public Health England for the duration of this research.

Ethics approval

The research published in this manuscript was approved by the PHE Research Support & Governance Office (Ref: NR0131) and the London School of Hygiene and Tropical Medicine Observational/Interventions Research Ethics Committee (Ref: 15839).

Permission for publication

Not applicable

Data availability statement

Data collection tools and some anonymised data are available on request via the LSHTM public repository.

References

1. Lu, B., et al., *Efficacy and safety of prophylactic vaccines against cervical HPV infection and diseases among women: a systematic review & meta-analysis.* BMC infectious diseases, 2011. **11**: p. 13-13.
2. Gellin, B., et al., *Quadrivalent Human Papillomavirus Vaccine.* Clinical Infectious Diseases, 2007. **45**(5): p. 609-617.
3. Paul, P. and A. Fabio, *Literature review of HPV vaccine delivery strategies: Considerations for school- and non-school based immunization program.* Vaccine, 2014. **32**(3): p. 320-326.
4. Paterson, P., et al., *Strengthening HPV vaccination delivery: findings from a qualitative service evaluation of the adolescent girls' HPV vaccination programme in England.* Journal of Public Health, 2019.
5. Bentley C, *Tackling gaps and inequalities in girls' uptake of the current HPV vaccination programme. Available at: http://emhf.org/wp-content/ uploads/2015/10/EMHF-HPV-Symposium-April-2015- REPORT.final_.pdf (last accessed 29 June 2016).* 2015.
6. Ferrer, H.B., et al., *Barriers and facilitators to HPV vaccination of young women in high-income countries: a qualitative systematic review and evidence synthesis.* BMC public health, 2014. **14**: p. 700-700.

For peer review only - http://bmjopen.bmj.com/site/about/guidelines.xhtml

For peer review only

7. Batista Ferrer, H., et al., *Barriers and facilitators to uptake of the school-based HPV vaccination programme in an ethnically diverse group of young women.* Journal of public health (Oxford, England), 2016. **38**(3): p. 569-577.
8. Boyce, T. and A. Holmes, *Addressing Health Inequalities in the Delivery of the Human Papillomavirus Vaccination Programme: Examining the Role of the School Nurse.* PLOS ONE, 2012. **7**(9): p. e43416.
9. Brabin, L., et al., *The school nurse, the school and HPV vaccination: A qualitative study of factors affecting HPV vaccine uptake.* Vaccine, 2011. **29**(17): p. 3192-3196.
10. National Information Board, *Personalised Heatlh and Care 2020. Using Data and Technology to Transform Outcomes for Patients and Citizens: A Framework for Action*. 2014, HM Government, NHS: file:///C:/Users/sdbltcha/Downloads/national_information_board_s_personalised_health_and_care_2020_framework.pdf (accessed 16th Jan 2020).
11. Boyatzis, R., *Transforming qualitative information: Thematic analysis and code development*. 1998, Thousand Oaks, CA: Sage
12. Griffith, R., *What is Gillick competence?* Human vaccines & immunotherapeutics, 2016. **12**(1): p. 244-247.
13. Public Health England, *Consent: the green book, chapter 2*, in *The Green Book: Immunisation against infectious disease*. 2013, Public Health England: https://assets.publishing.service.gov.uk/government/uploads/system/uploads/attachment_data/file/144250/Green-Book-Chapter-2-Consent-PDF-77K.pdf (accessed 22nd October 2018).
14. May, C.R., et al., *Development of a theory of implementation and integration: Normalization Process Theory.* Implementation science : IS, 2009. **4**: p. 29-29.
15. Finch, T.L., et al., *Improving the normalization of complex interventions: measure development based on normalization process theory (NoMAD): study protocol.* Implementation Science, 2013. **8**(1): p. 43.
16. Mair, F.S., et al., *Factors that promote or inhibit the implementation of e-health systems: an explanatory systematic review.* Bulletin of the World Health Organization, 2012. **90**(5): p. 357-364.
17. Farr, M., et al., *Implementing online consultations in primary care: a mixed-method evaluation extending normalisation process theory through service co-production.* BMJ Open, 2018. **8**(3): p. e019966.
18. Osborne, S.P., Z. Radnor, and K. Strokosch, *Co-Production and the Co-Creation of Value in Public Services: A suitable case for treatment?* Public Management Review, 2016. **18**(5): p. 639-653.

Figures Legends

Figure 1: The Electronic consent intervention

Figure 2: Theory of Change for the e-consent intervention

For peer review only

Figure 1: The Electronic consent intervention

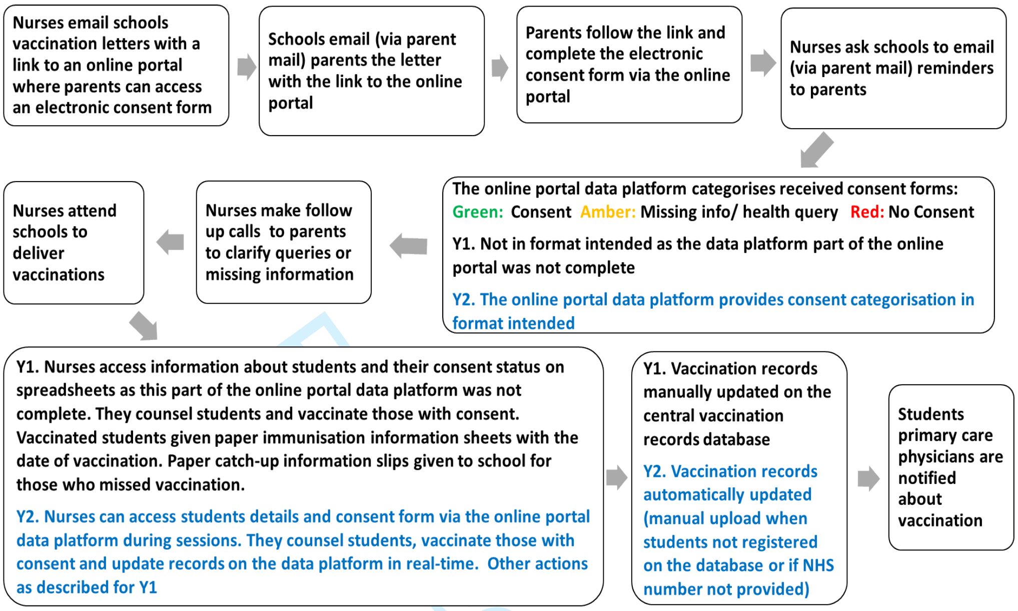

For peer review only

Figure 2: Theory of Change for the e-consent intervention

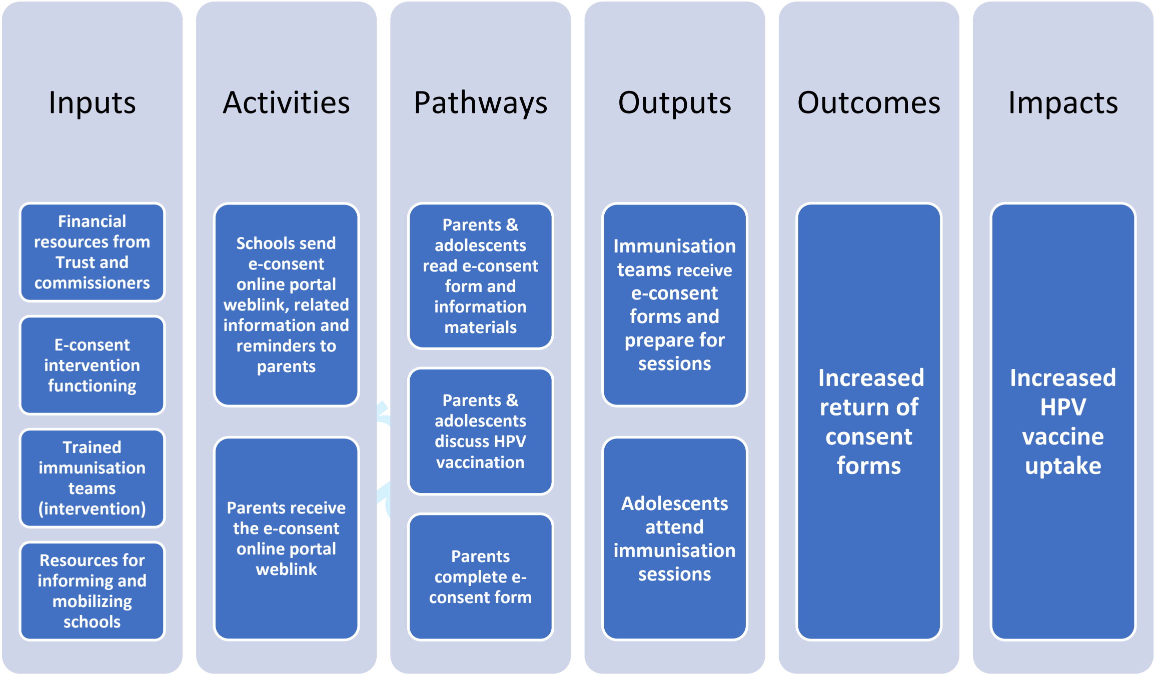

Additional File 1: School evaluation forms

DOES ELECTRONIC CONSENT IMPROVE THE LOGISTICS AND UPTAKE OF HPV VACCINATION IN ADOLESCENT GIRLS? A MIXED METHODS THEORY INFORMED EVALUATION OF AN INTERVENTION

The research published in this manuscript was approved by the PHE Research Support & Governance Office (Ref: NR0131) and the London School of Hygiene and Tropical Medicine Observational/Interventions Research Ethics Committee (Ref: 15839).

For peer review only

School evaluation form for e-consent schools

Introduction

The Hounslow and Richmond Community Health Trust is in the process of introducing a system that facilitates the collection of electronic consent for school-aged vaccinations. Your school has been involved in using the e-consent system as part of the HPV vaccination programme and we are keen to learn from this experience. We would be very grateful if you could complete this anonymous evaluation form and return it in the attached envelope to a member of the immunisation team at the end of the immunisation session today.

The information that you share with us will help us to evaluate the usability and acceptability of the e-consent system for school-based immunisations and assess its effectiveness in promoting consent form return and HPV vaccine uptake. The findings will guide decision-making about the future use of the e-consent system and help improve the system.

If you have any questions about this please do not hesitate to contact the research team who are based at the London School of Hygiene & Tropical Medicine.

Contacts:

Dr Tracey Chantler
LSHTM, 15-17 Tavistock Place
London, WC1H 9SH
tracey.chantler@lshtm.ac.uk

Sandra Mounier-Jack
LSHTM, 15-17 Tavistock Place
London, WC1H 9SH
sandra.mounier-jack@lshtm.ac.uk

Evaluation form

Part 1

On a scale of 1-10 (1 being poor, 10 being excellent) please rate the following by circling your number of choice. Please add comment to explain your ratings.

a) The organisation of the immunisation session

1	2	3	4	5	6	7	8	9	10
Poor				Average				Excellent	

Comments:

____
____
____
____

b) The usability of the e-consent system for obtaining consent for adolescent vaccination.

1	2	3	4	5	6	7	8	9	10
Poor				Average				Excellent	

Comments:

____
____
____
____

c) The efficiency of the e-consent system (in terms of reducing administrative burden and improving the organisation of immunisation sessions)

1	2	3	4	5	6	7	8	9	10
Poor				Average				Excellent	

Comments:

____
____
____
____

d) Communication between the immunisation team and the school

1	2	3	4	5	6	7	8	9	10
Poor				Average				Excellent	

Comments:

____
____
____
____

For peer review only

Part 2

a) What role have your school staff played in preparing for the HPV immunisation session?

1. Educational activities (*please tick*): Yes ☐ No ☐

If yes please state what these were (e.g. assembly):

____

____

____

____

2. Providing list of eligible students (*please tick*) Yes ☐ No ☐

3. Send reminders to parents (tick all relevant methods):

   a. Parent Mail ☐

   b. Post reminders to home address ☐

   c. Handing out reminders students ☐

   d. Text messages to parents/guardians ☐

   e. Phone call to parents/guardians ☐

   f. Email to parent/guardians with consent form link ☐

4. How many reminders did you send to non-respondents: No: ______________

5. Have you helped parents/adolescents access computers to be able to fill in the form online?

   Yes ☐ No ☐

b) What is your experience of using or facilitating use of the e-consent system? What do you think about obtaining consent for school-based teenage vaccination electronically?

____

____

____

____

c) What have parents and students said about the e-consent system?

____

____

____

____

For peer review only

d) Which means of obtaining consent do you think is more efficient: paper based or e-consent?

e) How do you think the system could be improved?

Thank you for completing this evaluation form

For peer review only

School evaluation form for paper-based consent schools

Introduction

The Hounslow and Richmond Community Health Trust is in the process of introducing a system that facilitates the collection of electronic consent for school-aged vaccinations. Your school is likely to use this system in the future hence we are keen to learn from your current experience of being involved in coordinating the HPV vaccination programme for adolescent girls. We would be very grateful if you could complete this anonymous evaluation form and return it in the attached envelope to a member of the immunisation team at the end of the immunisation session today.

The information that you share with us will help us to evaluate the usability and acceptability of the e-consent system for school-based immunisations and assess its effectiveness in promoting consent form return and HPV vaccine uptake. The findings will guide decision-making about the future use of the e-consent system and help improve the system.

If you have any questions about this please do not hesitate to contact the research team who are based at the London School of Hygiene & Tropical Medicine.

Contacts:

Dr Tracey Chantler
LSHTM, 15-17 Tavistock Place
London, WC1H 9SH
tracey.chantler@lshtm.ac.uk
07969404745

Sandra Mounier-Jack
LSHTM, 15-17 Tavistock Place
London, WC1H 9SH
sandra.mounier-jack@lshtm.ac.uk
020 7927 2929

Rosie Cooper
Address to be added
rosie.cooper@phe.gov.uk

For peer review only

Evaluation form

Part 1

On a scale of 1-10 (1 being poor, 10 being excellent) please rate the following by circling your number of choice. Please add comments to explain your ratings.

e) The organisation of the immunisation session

1	2	3	4	5	6	7	8	9	10
Poor				Average				Excellent	

Comments:

f) The usability of the current paper-based system for obtaining consent for adolescent vaccination.

1	2	3	4	5	6	7	8	9	10
Poor				Average				Excellent	

Comments:

g) The efficiency of the paper-based system (in terms of administrative burden and organisation of immunisation sessions)

1	2	3	4	5	6	7	8	9	10
Poor				Average				Excellent	

Comments:

h) Communication between the immunisation team and the school

1	2	3	4	5	6	7	8	9	10
Poor				Average				Excellent	

Comments:

For peer review only

Part 2

a) What role have your school staff played in preparing for the HPV immunisation session?

1. Educational activities (*please tick*): Yes ☐ No ☐

If yes please state what these were (e.g. assembly):

____
____
____
____

2. Providing list of eligible students (*please tick*) Yes ☐ No ☐

3. Send reminders/consent forms to parents (tick all relevant methods):

   a. Parent Mail ☐

   b. Post reminders/consent forms to home address ☐

   c. Handing out reminders/consent forms to students ☐

   d. Text messages to parents/guardians ☐

   e. Phone call to parents/guardians ☐

   f. Email to parent/guardians with consent form attached ☐

4. How many reminders did you send to non-respondents: No: ______________

b) What is your experience of using or facilitating use of the existing consent system?

____
____
____
____
____

c) What do you think about obtaining consent for school-based teenage vaccination electronically?

____
____
____
____
____

Thank you for completing this evaluation form

For peer review only

Additional File 2: Interview Topic Guides

DOES ELECTRONIC CONSENT IMPROVE THE LOGISTICS AND UPTAKE OF HPV VACCINATION IN ADOLESCENT GIRLS? A MIXED METHODS THEORY INFORMED EVALUATION OF AN INTERVENTION

The research published in this manuscript was approved by the PHE Research Support & Governance Office (Ref: NR0131) and the London School of Hygiene and Tropical Medicine Observational/Interventions Research Ethics Committee (Ref: 15839).

Interviewees: Immunisation team members

For peer review only

Experience of obtaining consent in school-based vaccination programmes

- Could you tell me about your experience of obtaining consent from parents and adolescents for school-aged vaccinations?
    - What has worked well and what has been challenging?
    - How have you overcome challenges in the past?

Acceptability of the e-consent system

- What were your initial thoughts about the e-consent system? (Probes: concerns, hopes, expectations)
- What did your colleagues think about the system?
- How acceptable do you think the system is for parents, teenagers, and school staff involved in the programme?

Usability of the e-consent system

- How helpful was the training in preparing you to use the e-consent system?
- What was your experience of using the e-consent system? What worked well, what was difficult in the following stages?
    - Triage of consent forms
    - Use of the system during immunisation sessions in schools
    - Uploading data to Trust databases and Child Health Immunisation Systems
    - Any other stages
- What contact did you have with parents about the use of the e-consent system? How did parents find using the e-consent system? What did they find difficult?
- What do you think about how the system may support communication between parents/guardians and adolescents about immunisation? How does this compare with the paper-based system?
- How does the e-consent system support communication between immunisation teams, parents and students? How does this compare with the paper-based system?
- What are the advantages and disadvantaged of the e-consent system in comparison with the paper-based system?

Interaction between schools and immunisation teams

- What is your experience of working with schools in organising immunisation sessions?
- What were schools reactions to the introduction of the e-consent system?
- How has the e-consent system supported interactions between schools and immunisation teams? (Probes: what has worked well, what has been challenging?) How does this compare with the paper-based system?
- What have schools told you about their experience of using the e-consent system? How do they compare it with the paper-based system?
- Have schools conducted any educational activities in preparation for the HPV immunisation visits? What were these? How have you been involved in these?

Adolescent self-consent

- What do you think about giving teen-agers the opportunity to self-consent for vaccination? (Probes: age, what vaccines e.g. HPV)
- Have you obtained self-consent for adolescent immunisation? If yes, in what instances, what was the procedure, and how did you assess for competency?
- How does the e-consent system support self-consent? What is the procedure for this? Did any teen-agers use the e-consent system to give consent for HPV vaccination? What was that experience like?
- Have you had conversations with schools about adolescent self-consent? What are their views?
- Have you had conversations with parents/guardians about adolescent self-consent? What were their views?

Reflections of the use of the e-consent system

- What have been the main lessons in piloting the e-consent system (Probes: what has worked well, what needs to be changed?)
- How would you modify and improve the e-consent system based on the piloting experience?
- What is your advice for other immunisation providers thinking about implementing e-consent?
- Has the e-consent system achieved its intended outcomes from your perspective?

Any other comments

Do you have any other comments?

Thank you for your time.

For peer review only

Interviewees: Service managers

Experience of obtaining consent in school-based vaccination programmes

- Could you tell me about your past experience of managing the logistics of obtaining consent from parents and adolescents for school-aged vaccinations?
  - What has worked well and what has been challenging?
  - How have you overcome challenges in the past?

Decision to launch the e-consent system

- What were the main reasons you decided to implement the e-consent system?
- How did you decide who to collaborate with in developing the e-consent system?
- How was the process of development? What went well, what was difficult?
- How did your test the system prior to the piloting phase?

Acceptability of the e-consent system

- How did your team initially react to the plans to introduce the e-consent system? (Probes: Expectations, concerns, questions)
- Did opinion change over time, and if yes, how?
- How acceptable do you think the e-consent system is for nurses, parents, teenagers, and school staff involved in the programme?

Usability of the e-consent system

- How were you involved in using the e-consent system?
- What was your/your teams' experience of using the e-consent system? What worked well, what was difficult in the following stages?
  - Triage of consent forms
  - Use of the system during immunisation sessions in schools
  - Uploading data to Trust databases and Child Health Immunisation Systems
  - Any other stages
- What contact did you/your team have with parents about the use of the e-consent system? How did parents find using the e-consent system? What did they find difficult?
- What do you think about how the system may support communication between parents/guardians and adolescents about immunisation? How does this compare with the paper-based system?
- How does the e-consent system support communication between immunisation teams, parents and students? How does this compare with the paper-based system?
- What are the advantages and disadvantaged of the e-consent system in comparison with the paper-based system?

For peer review only

Interaction between schools and immunisation teams

- What is your experience of working with schools in organising immunisation sessions?
- What were schools reactions to the introduction of the e-consent system? How did you inform them about the e-consent system, what guidance or training did you provide?
- How has the e-consent system supported interactions between schools and immunisation teams? (Probes: what has worked well, what has been challenging?)
- How does this compare with schools that are using the paper system?
- What have schools told you about their experience of using the e-consent system? How do they compare it with the paper-based system?
- Have schools (those using e-consent and paper system) conducted any educational activities in preparation for the HPV immunisation visits? What were these? How have you/your team been involved in these?

Adolescent self-consent

- Please describe the process you have used for self-consent
- What do you think about giving teenagers the opportunity to self-consent for vaccination? (Probes: age, what vaccines e.g. HPV)
- Have you had conversations with schools about adolescent self-consent? What are their views?
- Have you had conversations with parents/guardians about adolescent self-consent? What were their views?

Reflections of the use of the e-consent system

- What have been the main lessons in piloting the e-consent system (Probes: what has worked well, what needs to be changed?)
- How will you modify and improve the e-consent system based on the piloting experience?
- What have been the costs of implementing the e-consent system?
- What is your advice for other immunisation providers thinking about implementing e-consent?
- Has the e-consent system achieved its intended outcomes from your perspective?

Any other comments

Do you have any other comments?

Thank you for your time.

For peer review only

Interviewees: Service administrators

Experience of managing the administration of the school-based vaccination programmes

- Could you tell me about your past experience of managing the administration of the school-aged vaccination programme?
    - What has worked well and what has been challenging?
    - How have you overcome challenges in the past?

Decision to launch the e-consent system (for senior administrators)

- What were the main reasons the Trust decided to implement the e-consent system?
- How was the process of development? What went well, what was difficult?
- How did your test the system prior to the piloting phase?

Acceptability of the e-consent system

- What were your initial thoughts about the plans to introduce the e-consent system? (Probes: Expectations, concerns, questions)
- Did your opinion change over time, and if yes, how?
- What is your current view on the acceptability of the e-consent system?

Usability of the e-consent system

- How are you involved in using the e-consent system?
- What was your/your teams' experience of using the e-consent system? What worked well, what was difficult in the following stages?
    - Administration of data from the consent forms
    - Administration of data from school and catch-up immunisation sessions
    - Uploading data to Trust databases and Child Health Immunisation Systems
    - Sharing data with GPs
    - Any other stages
- What are the advantages and disadvantaged of the e-consent system in comparison with the paper-based system?

Interaction between schools and immunisation teams

- What role do you play in collaborating with schools to organising immunisation sessions?
- What were schools reactions to the introduction of the e-consent system? How were they informed about the e-consent system, what guidance or training did they receive?
- How has the e-consent system supported interactions between schools and immunisation providers? (Probes: what has worked well, what has been challenging?)
- How does this compare with schools that are using the paper system?
- What have schools told you about their experience of using the e-consent system? How do they compare it with the paper-based system?

For peer review only

Reflections of the use of the e-consent system

- What have been the main lessons in piloting the e-consent system (Probes: what has worked well, what needs to be changed?)
- How will you modify and improve the e-consent system based on the piloting experience?
- What have been the costs of implementing the e-consent system?
- What is your advice for other immunisation providers thinking about implementing e-consent?
- Has the e-consent system achieved its intended outcomes from your perspective?

Any other comments

Do you have any other comments?

Thank you for your time.

For peer review only

Interviewees: Parents/guardians

Views on adolescent vaccination

- What do you know about the vaccines that are offered to teenagers? (Probes: HPV, teenage booster, Men ACWY)
- What information have you received about teenage vaccinations? Who gave you this information? Did you search for additional information, if yes where did you look and what did you find?
- What is your view about adolescent vaccination? Are there any vaccines that you are concerned about?

Experience of providing consent for adolescent vaccinations

- Could you tell me about your experience of providing consent for your teenager(s) to be vaccinated?
- How did you make your decision about providing consent?
    - Who else did you talk to about this? What did you discuss? What were their views? (Probes: Partner, teenager, other family members, friends, health professionals)
- How did you involve your teenager in this decision-making? Were you in agreement about having the vaccine?
- Have you ever changed your mind about providing consent for a vaccination, if yes why and what did you do?

Usability of the e-consent system

- How did you find using the e-consent system? Who completed the form?
- Was it easy to access the e-consent site via the email sent from school?
- What was you experience of completing the consent form online? Were there any parts that you found difficult to complete? How did you know if you had completed the form properly?
- What was your experience of accessing the HPV vaccination programme leaflets via the e-consent system? Did you read these online or did you print them out? Did you share these leaflets with your teenager or other members of the family?
- Did you contact the immunisation team for help to complete the e-consent form? What support did you require? How easy was it to contact them?
- How do you think that the e-consent form could be improved?

Acceptability of the e-consent system

- What are your views on giving consent electronically for your teenagers' vaccinations? (Probes: any concerns about sharing personal data online?)
- What did your teen-ager think about this method of giving consent?
- What are your views on how the e-consent system supports information sharing and communication about teenage vaccination?

For peer review only

Teenagers experience of immunisation in school

- What did your teenager tell you about their experience of being immunised in school? (Probes: what went well, what did they find difficult)
- How well prepared do you think your teenager was prior to the immunisation session? (Probes: access to information, educational sessions in school)

Views on adolescent self-consent

- What do you think about the option of teen-agers providing self-consent for vaccination?
- What is your understanding of self-consent in adolescence?
- Would you be happy for your teen-ager to give self-consent for vaccination? If yes, why. If no, why?

Any other comments

Do you have any other thoughts about adolescent vaccination and how consent is obtained for this?

Thank you for your time.

For peer review only

Interviewees: Adolescents (questions asked with parents)

Views on adolescent vaccination

- What do you know about the vaccines that are offered to teenagers? (Probes: HPV, teenage booster, Men ACWY)
- What information have you received about the HPV vaccination? Who gave you this information? Have you accessed any other information, if yes what was this?
- What is your view about the HPV vaccination? (Probes: what is good about it, any concerns about the vaccine or the process of vaccination)
- Who have you talked to about having the HPV vaccine? (Probes: parents, friends, school nurse, other health professionals, other family members) What did you discuss?

Experience of giving consent for vaccination

- Who decided whether you would receive the HPV vaccine? How did they make this decision?
- How were you involved in this decision-making? Were you in agreement with your parents about this decision?

Usability of the e-consent system

- Were you involved in using the e-consent system? If yes, how were you involved either at home or in school and what did you think about this system?
- What did your parents think about the e-consent system? Were there any parts of the online form that they found difficult to complete?
- Were you given the opportunity to find out more about the HPV vaccine via the e-consent system? (Probes: Access leaflets, read and discuss these with your parents)
- What do your friends think about the e-consent system?
- How do you think that the e-consent system could be improved?

Acceptability of the e-consent system

- What are your views on giving consent online for your vaccinations? (Probes: any concerns about sharing personal data?)
- What are your views on how the e-consent system supports finding out about and talking about teenage vaccination?

Experience of the immunisation session

For peer review only

- Could you tell me a bit about your experience of receiving the HPV vaccine in school? What was the session like, how did you feel, what went well, what was difficult?
- How well prepared were you for the immunisation session? (Probe: know you would be vaccinated against HPV, understood what the vaccine was for any why you needed it)
- Were you given the opportunity to ask any questions before you were vaccinated?
- Had you participated in any information sessions about vaccination at school before you were vaccinated? If yes, what were these and what did you learn?
- Had you received any other information about the HPV vaccine before you were vaccinated? If yes, who gave you this?

Views on adolescent self-consent

- What do you think about the option of teen-agers providing self-consent for vaccination?
- What is your understanding of self-consent?
- Have you ever been given the option to provide self-consent for vaccination?
- Would you be happy to provide self-consent for vaccination? If yes, why. If no, why?

Any other comments

Do you have any other thoughts about vaccination and how consent is obtained for this?

Thank you for your time

For peer review only

Adolescent Focus Group Discussion Topic Guide

Interviewees: Adolescents (Year 8/9 girls)

Views on adolescent vaccination

- What do you know about the vaccines that are offered to teenagers? (Probes: HPV, teenage booster, Men ACWY)
- What information have you received about the HPV vaccination? Who gave you this information? Have you accessed any other information, if yes what was this?
- What is your view about the HPV vaccination? (Probes: what is good about it, any concerns about the vaccine or the process of vaccination)
- Who have you talked to about having the HPV vaccine? (Probes: parents, friends, school nurse, other health professionals, other family members) What did you discuss?

Experience of giving consent for vaccination

- Who decided whether you would receive the HPV vaccine?
- How were you involved in this decision-making? Were you in agreement with your parents about this decision?

Usability of the e-consent system

- Were you involved in using the e-consent system? If yes, how were you involved either at home or in school and what did you think about this system?
- What did your parents think about the e-consent system? Were there any parts of the online form that they found difficult to complete?
- Were you given the opportunity to find out more about the HPV vaccine via the e-consent system? (Probes: Access leaflets, read and discuss these with your parents)
- What do your friends think about the e-consent system?
- How do you think that the e-consent system could be improved?

Acceptability of the e-consent system

- What are your views on giving consent online for your vaccinations? (Probes: any concerns about sharing personal data?)
- What did your parents think about this way of obtaining consent?

Experience of the immunisation session

- Could you tell me a bit about your experience of receiving the HPV vaccine in school? What was the session like, how did you feel, what went well, what was difficult?
- How well prepared were you for the immunisation session? (Probe: know you would be vaccinated against HPV, understood what the vaccine was for any why you needed it)
- Were you given the opportunity to ask any questions before you were vaccinated?

For peer review only

- Had you participated in any information sessions about vaccination at school before you were vaccinated? If yes, what were these and what did you learn?
- Had you received any other information about the HPV vaccine before you were vaccinated? If yes, who gave you this?

Views on adolescent self-consent

- What do you know about the option of teenagers providing self-consent for vaccination?
- Do you know what self-consent means?
- Would you be happy to provide self-consent for vaccination? If yes, why. If no, why?

Any other comments

Do you have any other thoughts about vaccination and how consent is obtained for this?

Thank you for your time.

For peer review only

BMJ Open

DOES ELECTRONIC CONSENT IMPROVE THE LOGISTICS AND UPTAKE OF HPV VACCINATION IN ADOLESCENT GIRLS? A MIXED METHODS THEORY INFORMED EVALUATION OF A PILOT INTERVENTION

Journal:	*BMJ Open*
Manuscript ID	bmjopen-2020-038963.R1
Article Type:	Original research
Date Submitted by the Author:	27-Aug-2020

Complete List of Authors:	Chantler, Tracey; London School of Hygiene & Tropical Medicine, Global Health & Development<br>Pringle, Ellen; Public Health England, Department of Immunisation, Hepatitis and Blood Safety, Public Health England, UK<br>Bell, Sadie; London School of Hygiene and Tropical Medicine Faculty of Public Health and Policy, Global Health and Development<br>Cooper, Rosie; University Hospitals of Derby and Burton NHS Foundation Trust<br>Edmundson, Emily; Hounslow and Richmond Community Healthcare NHS Trust<br>Nielsen, Heidi; Hounslow and Richmond Community Healthcare NHS Trust<br>Roberts, Sheila; Hounslow and Richmond Community Healthcare NHS Trust<br>Edelstein, Michael; Public Health England, Immunisation and countermeasures division<br>Mounier-Jack, Sandra; London School of Hygiene and Tropical Medicine,

**Primary Subject Heading**:	Public health
Secondary Subject Heading:	Health services research
Keywords:	Public health < INFECTIOUS DISEASES, Information technology < BIOTECHNOLOGY & BIOINFORMATICS, Paediatric infectious disease & immunisation < PAEDIATRICS

SCHOLARONE™
Manuscripts

I, the Submitting Author has the right to grant and does grant on behalf of all authors of the Work (as defined in the below author licence), an exclusive licence and/or a non-exclusive licence for contributions from authors who are: i) UK Crown employees; ii) where BMJ has agreed a CC-BY licence shall apply, and/or iii) in accordance with the terms applicable for US Federal Government officers or employees acting as part of their official duties; on a worldwide, perpetual, irrevocable, royalty-free basis to BMJ Publishing Group Ltd ("BMJ") its licensees and where the relevant Journal is co-owned by BMJ to the co-owners of the Journal, to publish the Work in this journal and any other BMJ products and to exploit all rights, as set out in our licence.

The Submitting Author accepts and understands that any supply made under these terms is made by BMJ to the Submitting Author unless you are acting as an employee on behalf of your employer or a postgraduate student of an affiliated institution which is paying any applicable article publishing charge ("APC") for Open Access articles. Where the Submitting Author wishes to make the Work available on an Open Access basis (and intends to pay the relevant APC), the terms of reuse of such Open Access shall be governed by a Creative Commons licence – details of these licences and which Creative Commons licence will apply to this Work are set out in our licence referred to above.

Other than as permitted in any relevant BMJ Author's Self Archiving Policies, I confirm this Work has not been accepted for publication elsewhere, is not being considered for publication elsewhere and does not duplicate material already published. I confirm all authors consent to publication of this Work and authorise the granting of this licence.

For peer review only

DOES ELECTRONIC CONSENT IMPROVE THE LOGISTICS AND UPTAKE OF HPV VACCINATION IN ADOLESCENT GIRLS? A MIXED METHODS THEORY INFORMED EVALUATION OF A PILOT INTERVENTION

Authors

Tracey Chantler[1] [*] [§], Ellen Pringle [*] [2]

Sadie Bell[1], Rosie Cooper[3], Emily Edmundson[4], Heidi Nielsen[4], Sheila Roberts[4], Michael Edelstein[1,2], Sandra Mounier Jack[1]

1. Department of Global Health and Development, Faculty of Public Health and Policy, London School of Hygiene & Tropical Medicine, 15-17 Tavistock Place, London, WC1H 9SH, UK
2. Department of Immunisation, Hepatitis and Blood Safety, Public Health England, UK
3. University Hospitals of Derby and Burton NHS Foundation Trust, Uttoxeter Road, Derby, DE22 3NE
4. Hounslow Richmond Community Healthcare NHS Trust, 18 High Street, Teddington, London, TW118HU, UK

*Joint first authors

§Corresponding author: Tracey Chantler, Department of Global Health and Development, Faculty of Public Health and Policy, London School of Hygiene & Tropical Medicine, 15-17 Tavistock Place, London, WC1H 9SH, UK, tracey.chantler@lshtm.ac.uk Tel: 07969404745

Word count: 4168

For peer review only

For peer review only - http://bmjopen.bmj.com/site/about/guidelines.xhtml

Abstract

Objectives

To evaluate the usability and acceptability of an electronic consent pilot intervention for school-based immunisations and assess its impact on consent form returns and HPV vaccine uptake.

Design

Mixed-methods theory-informed study applying qualitative methods to examine the usability and acceptability of the intervention and quantitative methods to assess its impact.

Setting and participants

The intervention was piloted in 14 secondary schools in seven London boroughs in 2018. Intervention schools were matched with schools using paper consent based on the proportion of students with English as a second language and students receiving free school meals. Participants included nurses, data managers, school-link staff, parents and adolescents.

Interventions

An electronic consent portal where parents could record whether they agreed to declined vaccination and nurses could access data to help them manage the immunisation programme.

Primary and Secondary Outcome Measures

Comparison of consent form return rates and HPV vaccine uptake between intervention and matched schools.

Results

HPV vaccination uptake did not differ between intervention and matched schools, but timely consent form return was significantly lower in intervention schools (73.3% (n=11) vs 91.6% (n=11), p=0.008). The transition to using electronic consent was not straightforward, whilst schools and staff understood the potential benefits, they found it difficult to adapt to new ways of working which removed some level of control from schools. Reasons for lower consent form return in e-consent

For peer review only

schools included difficulties encountered by some parents in accessing and using the intervention. Adolescents highlighted the potential for electronic consent to by-pass their information needs.

Conclusions

The pilot intervention did not improve consent form return or vaccine uptake due to challenges encountered in transitioning to new working practice. New technologies require embedding before they become incorporated in everyday practice. A re-evaluation once stakeholders are accustomed with electronic consent may be required to understand its impact.

Keywords

Public health, Information technology, Paediatric infectious disease and immunisation

Strengths and limitations

- The use of a theory-informed mixed methods study design allowed us to measure the effect of a pilot e-consent intervention on immunisation performance and identify mechanisms that facilitated or impeded implementation.
- The study design allowed us to account for schools, nurses, data managers, parents and adolescents' experiences of using the e-consent technology in this evaluation.
- Data limitations include the lack of interviews with school staff to complement the feedback forms and not being able to access and interview parents who found the intervention more challenging to use.
- This evaluation was limited to one cycle of implementation, which did not allow us to account for the time it takes for new practices to become embedded. The number of schools who declined to pilot the intervention was also not recorded.

For peer review only - http://bmjopen.bmj.com/site/about/guidelines.xhtml

Introduction

Vaccination against human papilloma virus (HPV) provides long-term protection against cervical and other cancers and genital warts[1, 2]. In England HPV vaccination (2 doses 6 months apart) is offered to adolescents aged 12-14 years in the school-based immunisation programme, which is delivered by nurse led immunisation teams. Whilst the school delivery model is widely accepted[3], it raises logistical challenges regarding obtaining parental consent. Immunisation teams and schools identify all students, who are eligible for vaccination and then students are typically given a paper consent form that their parents/legal guardians need to sign indicating their acceptance or refusal of vaccination. The passage of the form from teachers to students to parents and back again can impact on form return and have a detrimental effect on vaccine uptake[4-7]. Follow-up by school staff and immunisation teams improves uptake but is resource intensive[8, 9]. In the context of the drive towards a “paperless NHS”[10], there is increasing interest in the potential for technological solutions, such as electronic consent (e-consent). We evaluated the usability and acceptability of a pilot e-consent intervention from the perspective of parents and adolescents, health professionals and schools, and assessed its impact on consent form returns and HPV vaccine uptake.

The electronic consent intervention

The e-consent intervention was developed by Hounslow and Richmond Community Healthcare NHS Trust (HRCH) with the support of a software development company in 2017/18 and piloted in their adolescent girls’ vaccination programme in June/July 2018. At the time of the study HRCH was responsible for administering this vaccination programme in secondary schools across eight boroughs in South London.

The e-consent intervention consisted of an online portal with an e-consent form and a data platform and related implementation procedures (Figure 1). Functionally it aimed to: 1) give parents access

For peer review only - http://bmjopen.bmj.com/site/about/guidelines.xhtml

to an online portal with information about the vaccination programme where they could register their adolescent and agree to or decline HPV vaccination; 2) give nurses electronic access to the portal to facilitate screening and enable them to update records during immunisation sessions; 3) enable automatic updating of central vaccination record databases. Parts of the online portal and data platform (specifically those relating to points 2 & 3) were not fully functioning before the intervention was first used in June 2018. Hence modifications had to be made to the way nurses screened students' information and consent forms before and during immunisation sessions. Figure 1 differentiates what happened in Year 1: June/July 2018 and in Year 2: June/July 2019 (blue text). In the quantitative part of evaluation, we focused on Year 1; the measurement of outcomes and impacts. In the qualitative part of the evaluation we captured how Year 1 experience informed adaptations to the intervention prior to reuse in Year 2.

Methods

Study design

This was a mixed-methods theory-informed evaluationstudy which used a 'Theory of Change' (Figure 2) as an evaluation framework. Quantitative methods were used to assess whether the pilot intervention increased consent form return and the uptake of the first dose of HPV vaccine in adolescent girls in June/July 2018 (Year 1). The steps involved in implementing the e-consent system, people's experiences of these, the interactions between inputs, activities, pathways and outputs, outcomes and impacts were investigated using qualitative methods in Year 1 and Year 2. The reason that the quantitative analysis only focused on Year 1 was that the schools that received the intervention between Year 1 & 2 changed.

For peer review only - http://bmjopen.bmj.com/site/about/guidelines.xhtml

School selection

The e-consent intervention was piloted in 14 secondary schools in seven South London boroughs in June/July 2018 (Year 1). Schools selection was purposive with the aim of including schools that differed in terms of denomination (private, state, grammar), type (mixed, single sex), socio demographic, size, vaccination uptake, and level of support to the programme.

Schools were divided into low, medium or high based on the proportion of pupils receiving free school meals and with English as an additional language. Each e-consent school was matched, as closely as possible, to a paper consent school in the same tertiles for both characteristics.

Quantitative methods & analysis

Data collection (Year 1)

At each vaccination session nurses completed a "tally sheet", with details of the consents received prior to or during the session, any absences and the number of vaccinations given. These tally sheets were completed for paper and e-consent schools.

In addition, we extracted the name of the school, date and time of consent form completion and type of consent (agreement by parent or adolescent self-consent or decline) for each consent form in the e-consent system. This non-identifiable information was combined with tally sheet data, which was manually transcribed into MS Excel. Where there were discrepancies or missing data this was checked with the immunisation teams and in the case of the e-consent schools the system data was used in preference to the tally sheet data.

Data analysis

Characteristics of the paper and e-consent schools were described in terms of proportion of pupils receiving free school meals, speaking English as an additional language, ethnicity and characteristics of the schools (religious affiliation and state/privately funded), using data from the Office of National

For peer review only

Statistics. (REF: https://www.gov.uk/government/statistics/schools-pupils-and-their-characteristics-january-2018).

For both paper and e-consent schools, we calculated: (i) the proportion of the pupils for whom a consent form had not been returned prior to the vaccination session, (ii) the proportion of pupils vaccinated at the planned session and (iii) the proportion of pupils for who a “yes” consent was received (prior to or on the day of the planned vaccination session).

We compared e-consent schools with paper consent schools in terms of timely form return, outcome of consent and vaccination uptake using Kruskall Wallis tests for statistical significance. Where data was missing from a school that school and its matched pair were excluded from the analysis. All analyses were done in MS Excel and Stata 15.

Qualitative methods & analysis

Data collection

Data collection occurred in June-December 2018 (Year 1) and June-July 2019 (Year 2). The first period coincided with and followed the first year of e-consent implementation, the second occurred during the use of e-consent in a different subset of schools.

June-July 2018 (Year 1)

Observations of e-consent school HPV immunisation sessions

Members of the evaluation team (RC, TC, SMJ) accompanied immunisation teams during seven e-consent school HPV immunisation sessions to obtain a contextual understanding of the implementation of the intervention. The evaluators documented what worked well and what if any problem-solving was applied during sessions.

School feedback forms from paper and e-consent schools

For peer review only - http://bmjopen.bmj.com/site/about/guidelines.xhtml

School staff involved in organising immunisation sessions were asked to complete a feedback form after the HPV immunisation sessions (Additional file 1). Forms included questions about the organisation of immunisation sessions and the usability and acceptability of e-consent and paper consent. Seven e-consent and seven paper consent schools completed forms.

Individual and peer group interviews with HRCH staff

These semi-structured interviews (SSI's) involved HRCH staff who were responsible for implementing the intervention. The programme manager was interviewed individually, the two data specialists together and member of four immunisation teams in peer-groups of two to four participants (12 interviewees) at their respective offices. In total 15 members of staff took part in hour long interviews.

For peer review only

Interviews with parents and adolescents from e-consent schools

These SSIs involved parents and adolescents who had used the e-consent system. Parents/legal guardians were asked to indicate their willingness to be contacted by researchers in the e-consent form (ticked a statement). Parents who responded positively were invited by email to take part in an interview to discuss their experience of using the e-consent system. A total of 12 interviews were conducted, nine with HPV vaccine acceptors and three with decliners. Four interviews were conducted in family homes, seven by telephone, and one by Skype video. Adolescents participated in five interviews.

June-July 2019 (Year 2)

Results from data collected in Year 1 informed the implementation of the e-consent intervention in 22 schools located in South London in the 2018/19 HPV vaccine programme. Only one of these schools had taken part in the 2018 pilot, the reasons for using the improved intervention in this school and not the other pilot schools was mainly due to the scheduling of the HPV vaccination sessions. The Trust also wanted to expose additional schools to the e-consent system. To reflect the

For peer review only - http://bmjopen.bmj.com/site/about/guidelines.xhtml

changes made to the intervention and its implementation two group interviews with HRCH staff (one with nine and the other with five members of staff) and one focus group discussion (FGD) with eight adolescent girls (from a school who a school that used the e-consent intervention in 2019 only) were conducted.

The topics covered in the staff, parent and adolescent SSIs and adolescent FGD are summarised in Text Box 1 (see Additional File 2 for Topic Guides).

Text box 1: Topics covered in interview

**Staff interviews**	**Parent and adolescent interviews**
• Experience of managing the administration of the school-based vaccination programme<br>• Experience of obtaining consent in school-based vaccination programme<br>• Acceptability and usability of the e-consent system<br>• Interaction between schools and immunisation teams<br>• Adolescent self-consent<br>• Reflections on use of the e-consent system	• Views on adolescent vaccination<br>• Understanding of adolescent vaccination<br>• Experience of providing consent for the HPV vaccine<br>• Acceptability and usability of the e-consent system<br>• Teenagers experience of immunisation in school<br>• Vaccine programme communication<br>• Views on adolescent self-consent

Data analysis

The SSIs and FGD were audio recorded with participant's permission and transcribed anonymously. Transcripts, observational field notes and school feedback forms were downloaded into a qualitative data analysis management software programme (NVivo 12). We adopted a thematic analytical approach which combined semi-deductive mapping of data to the 'input', 'activities', 'pathways', 'outputs', 'outcomes' and 'impacts' depicted in the ToC and inductive open coding to capture emerging themes[11]. We sought to account for the interdependence of ToC categories and real-life experience of managing organisational change, which do not always progress from inputs – impacts in a seamless linear manner.

For peer review only - http://bmjopen.bmj.com/site/about/guidelines.xhtml

Patient and public involvement

This evaluation was designed with members of HRCH who were responsible for coordinating the implementation of the e-consent intervention. This collaboration supported the iterative and reflexive development of the intervention, which is essential for longer-term integration.

Results

Quantitative

Participants

Twenty-eight schools (14 paper and 14 e-consent schools) comprising 3219 girls (1733 in paper consent and 1486 in e-consent schools) were included in the study. Of those schools, 26 were state and 2 were private schools. Twenty-one of the schools had no religious affiliation, three were Roman Catholic (all paper consent), three Church of England (two e-consent, one paper consent) and one was another Christian faith school (paper consent).

The proportion of pupils eligible for free school meals, with English as an additional language and students' ethnicity profile was similar between the e-consent and paper consent schools (Table 1).

Table 1: Characteristics of paper and e-consent schools[1,2]

	Paper consent schools (n=13)	E-consent schools (n=13)	P-value*
**% (range) of children eligible for free school meals**	17.3 (1.5-52.1)	17.3 (1.5-43.2)	0.84
**% (range) of children with English as additional language**	30.9 (5.5-53.6)	32.4 (9.1-59.2)	0.72
**% (range) of children of white British ethnicity**	30.9 (1.5-69.6)	34.6 (6.3-65.7)	0.63

*Refers to comparison of each characteristic using Kruskal-Wallis test

1. Characteristics information was not available for the two private schools
2. Data from Schools, pupils and their characteristics: January 2018, Department of Education

For peer review only - http://bmjopen.bmj.com/site/about/guidelines.xhtml

Return of consent forms ahead of session

Overall 83% of consent forms (paper or e-consent) were returned prior to the vaccination session. However, compared with paper schools timely (prior to the planned session) return was lower in the e-consent schools (73.3% (n=11) vs 91.6% (n=11), p=0.008). We could only measure this difference in 22 matched schools due to missing data.

Outcome of consent

There was no statistically significant difference in the proportion of pupils for whom a “yes” consent was received (prior to or on the day of the session) between the paper (n=14) and e-consent (n=14) schools (85% in e-consent schools, 83% in paper consent schools, p=0.89).

Vaccination uptake

There was no statistically significant difference in the proportion of pupils that were vaccinated at the scheduled vaccination session between the paper (n=14) and e-consent (n=14) schools (80.6% vs 81.3%, p=0.93). These figures did not include those who were absent on the day and vaccinated later[i]. The final vaccine uptake across all the schools was over 86%.

Qualitative

The results of the ToC thematic analytical mapping are presented under relevant headings (‘input’, ‘activities’, ‘pathways’, ‘outputs’, ‘outcomes’) followed by an overarching theme on managing change.

Inputs

Resources and Training

There was a *‘buzz’* about the development of the e-consent intervention, its potential to streamline consent and facilitate safer data collection and NHS England had provided funding for HRCH to pilot it during the 2017/18 HPV vaccine programme. Immunisation team members were positive but

For peer review only

expressed reservations about not able to review paper consent forms prior to immunisation. Due to tight deadlines only one orientation session took place before the e-consent intervention was introduced, which meant that the bulk of learning happened on the job.

"I think as well, it was probably four days before our first session, we didn't know what we were doing...so I do feel we are running before we can walk." (Immunisation Team 2)

Intervention not fully operational

The data platform component of the online portal was not operational prior to implementation. Parents were able to access and complete the e-consent form, but the immunisation teams could not review student's online consent forms or upload data during immunisation sessions. Instead large (A3) paper sheets with information about who had provided consent were prepared by the data managers. The sheets were difficult for to decipher during busy sessions and nurses were less able to prepare cohort figures and tally sheets in advance.

"It was an anti-climax not being able to use the laptops and still have a paper sheet in front of me." (Immunisation Team 4)

Mobilizing and resourcing schools

There was limited time to collaborate with schools before the vaccination programme, although all e-consent schools were guided on how to disseminate the weblink. A few schools in 2 inner city boroughs declined to use the intervention due to concerns about: i) pre-existing barriers to electronic communication with parents, ii) whether a change to consent processes would reduce the return of consent forms and uptake of HPV vaccine, iii) adapting to a new way of working.

School immunisation link staff reported a *'loss of control'*, for example they could no longer see *'who had said yes, and who had said no'*, which restricted their ability to follow-up unreturned forms. Paper-consent schools could monitor this directly by counting forms, but with e-consent schools

For peer review only - http://bmjopen.bmj.com/site/about/guidelines.xhtml

immunisation teams had to check parental responses and tell schools which families had not replied.

Activities

Dissemination of the online e-consent portal weblink to parents

E-consent schools used different means (parent mail, email, school website, newsletters, letters) to send parents the portal weblink. Blanket reminders were mainly sent electronically, unless immunisation teams provided schools with details of non-responders. In this case follow-up could be more targeted and involve text messages and phone calls as wells as emails. One school used a translator to engage parents who did not understand the consent process due to language barriers. Another school was not willing to send out emails and asked the immunisation team to provide them with printed letters referring to the weblink to send to parents.

Of the seven e-consent schools who completed the feedback form, four were positive about the intervention and how it had been implemented stating that it had reduced their workload. Another school was mainly positive but noted that some parents had found the e-consent form difficult to access, another reported that their parent cohort had found the system very difficult to access and use, and the last school was the one who had used letters to disseminate the weblink.

Pathways

Navigating the e-consent form and related information

The e-consent form included links to an HPV vaccination leaflet. However, none of the interviewees had downloaded or read this leaflet for the following reasons: accessed information elsewhere, already sufficiently informed, older daughter vaccinated, positive about vaccination.

Proactive information seeking involving a wider range of sources was more common in families who were vaccine hesitant. Their concerns, which in some cases resulted in vaccine refusal, included the following: i) compatibility of vaccination with pre-existing illness, ii) unknown side-effects (e.g.

For peer review only - http://bmjopen.bmj.com/site/about/guidelines.xhtml

hormonal interferences while daughter establishing her cycle), iii) targeting of adolescents due to their ethnicity (nurses explained that some parents of Black-Caribbean origin were sceptical of any state intervention, like vaccination), iv) necessity of vaccination for adolescents who are not sexually active. Parents who were more confident about vaccines restricted their information seeking to NHS sources and suggested that a 'road map' to adolescent vaccination could be useful.

Adolescents reported a variety of information seeking behaviours. Some just accepted HPV vaccination as '*something that needs to be done'* and felt reassured that it was recommended by the NHS: *"I think because it's like by the NHS - it kind of gives it validation." (Adolescent 9 - Yes)*. Others wanted the HPV vaccine leaflet to include more information about HPV and related health risks and vaccine side-effects, so that they did not panic if they experienced any of these.

In the FGD students expressed a preference for paper leaflets and discussed how the e-consent could bypass them: *"…because like if it's emailed, like your mum doesn't have to share it with you. And like if I have something done like an injection, I'd like to know what's going on and when. But like she filled out the form without like telling me, so like if they'd been given out in school then I could have read it and see what's happening."*

These FGD participants wanted to have the opportunity to discuss vaccination with their parents; they were not seeking autonomy in decision-making rather wanted a degree of joint responsibility.

"I wouldn't like to be given the option to like not to have the injection done…so I'm kind of glad that my mum just decided like on her own. But I would have liked her to talk it through with me…" (FGD participant)

Parents approaches to talking to their daughters about having an HPV vaccination are summarised in Text Box 2.

For peer review only - http://bmjopen.bmj.com/site/about/guidelines.xhtml

Text Box 2: Parents approaches to discussing HPV vaccination with adolescent daughters

Very limited discussion

- Did not talk to daughter in detail since vaccination is a parental decision;
- Talking too much about pending vaccination may induce anxiety, especially if needle phobic.

Heads up as what to expect

- Ensured daughter knew what to expect; this exchange usually occurred shortly before the immunisation session;
- Offered to answer any questions depending on adolescents' desire to know more.

More in-depth discussions

- Joint decision-making between parents and adolescents about the important of vaccination, this sometimes involved accessing additional information.

For peer review only

Using the e-consent intervention

The parents we interviewed found the system easy to use and usually completed the form as soon as they received it. A few parents would have liked an email confirmation after they had completed the e-consent form.

"I thought it was very easy. I think you're probably going to get more responses that way from parents in this day and age. However, the downside is obviously you may not get that chance to discuss it. (Parent 5 – Yes)

According to feedback from nurses and schools not all parents found the intervention easy to access or use. Language barriers accounted for some difficulties, but practical issues also played a role, for example some parents had not signed up for the school parent mail system hence did not receive the weblink. Nurses also received a significant number of calls from parents reporting that the weblink would not open or that webpages froze. During an immunisation session one student stated: *"my dad said I should have the vaccine, but he did not understand the whole google business about it".* In some instances, the weblink closed a few days prior to an immunisation session to give immunisation teams time to screen student information prior to sessions. This resulted in some

For peer review only - http://bmjopen.bmj.com/site/about/guidelines.xhtml

parents who had missed the last sign-up date sending in written notes to confirm their consent/non-consent for vaccination.

Outputs

Nurses access to e-consent forms and student information

The immunisation sessions at e-consent schools were affected by nurses not being able to access electronic information about students before and during sessions. The lower return of consent forms in e-consent schools also resulted in nurses reporting that they had to contact more parents than usual during immunisation sessions to obtain verbal consent. This had implications for the nurses' workload distribution and the length of sessions. To manage these challenges immunisation teams increased the number of nurses and administrative assistants who attended e-consent school immunisation sessions.

"…we had 80 consent forms outstanding at a big school. But, normally, if you only have a couple it's fine. It meant us was making calls all morning, it took a nurse out of immunising to be able to do that, so that did have a big impact." (Immunisation Team 2)

Conducting phone calls during sessions was not straightforward. Firstly, nurses had to rely on students (if they had phones with them) or staff to help them access correct contact details. Secondly, immunisations sessions were busy and noisy which impeded communication and privacy. Thirdly, it was not always possible to reach parents who were at work or out of the house during day-time hours. If parents were uncontactable the nurses assessed if students who wanted to be vaccinated had sufficient maturity and intelligence to understand and appraise the nature and implications of the proposed vaccination[12, 13]. This process was time-consuming and not all nurses felt comfortable about vaccinating without verbal or written parent permission.

For peer review only - http://bmjopen.bmj.com/site/about/guidelines.xhtml

Transition: adapting to change and iterative development

The initial 'buzz' about the e-consent intervention decreased over time. Whilst some staff remained positive and receptive to the implementation coordinators enthusiasm and vision, others expressed a sense of half-heartedness about having to adapt quickly from a known way of obtaining consent, albeit with flaws (e.g. cost of paper, mileage clocked up in collection paper consent forms from schools), to a new technology enabled way with some functional limitations in Year 1 (see Figure 1).

In the HRCH staff group interviews conducted in July 2019 members of the immunisation teams reflected on lessons learnt from their experience of transitioning to a *'brand new way of working'* over the past year. Key learning points from an internal organisational perspective were: (i) adopt right pace of progress when introducing new interventions with have several components, (ii) be clear about which part of a multi-component intervention is being piloted and implemented (e.g. in Year 1 it was primarily about the e-consent form), (iii) importance of timely communication, quick thinking, and flexibility when things do not quite go to plan.

In terms of school engagement HRCH staff emphasised the importance of close collaboration to devise appropriate means of consent in different educational and social contexts. The right balance of responsibility between schools and immunisation teams needs to be negotiated to maintain positive working relationships and ensure that adolescents can access essential vaccines.

"I would also say the idea of just changing to e-consent… schools need different things… it is really important to work with the school and a make sure that they are happy with everything and it suits that school, because some schools it might just not suit right now. It might suit them in a couple of years, but right now it just doesn't work." (HRCH immunisers group interview, July 2019)

HRCH made several changes to the implementation of the e-consent intervention in Year 2 (2018/19) informed by evaluation findings (Text Box 3).

For peer review only - http://bmjopen.bmj.com/site/about/guidelines.xhtml

For peer review only

Text Box 3:
E-consent intervention implementation changes between Yr1 and Yr2

- ❖ Taking more time to engage (emails, phone calls and meetings) with schools in preparations to find the right level of involvement
- ❖ Ensuring students receive a paper copy of the HPV adolescent programme leaflet produced by Public Health England in addition to information provided in the e-consent form
- ❖ Pushing more for assemblies and contact with adolescent girls prior to the immunisation sessions
- ❖ Providing ongoing training and mentoring of immunisation teams on use of the intervention

Discussion

The pilot e-consent intervention had no detected impact on vaccine uptake and did not improve the return rate of consent forms in its first year of implementation. This could be interpreted as a negative result, however this would fail to account for the embedding process required before new technologies become (or do not become) routinely incorporated into everyday practice[14], and inherent difficulties in achieving high vaccine uptake in urban centres. Our findings demonstrate that the transition from paper to e-consent was not straightforward, and that staff and schools, though mainly open to change, took time to adapt. The transition was hampered by the reduced functionality of the IT capability (intervention fidelity), limited staff training and engagement of schools. It is likely that this e-consent intervention would need to be used for more than one year to achieve any benefits to process efficiency and vaccine uptake.

This study starts to address an evidence gap and demonstrates shows how real-time evaluation can support the iterative development of interventions essential for their longer-term integration. Key limitations include the lack of interviews with school staff to complement the feedback forms and not being able to access and interview parents who found the intervention more challenging to use.

The 'Normalisation Process Theory' (NPT) argues that new practices become routinely embedded as the result of people working, individually and collectively, to enact them. Enactment is promoted or

For peer review only - http://bmjopen.bmj.com/site/about/guidelines.xhtml

inhibited through the operation of four mechanisms; coherence (sense-making), cognitive participation (individual buy-in), collective action (joint effort) and reflexive monitoring (user appraisal)[15]. Sense-making requires users to have a shared understanding concerning the purpose of an intervention, their responsibilities in its implementation, its potential benefits and the differences to existing practice[16]. In our study, immunisation teams recognised the potential value of the intervention but were not clear about their specific responsibilities and schools had to make sense of the change in their role in consent logistics. User buy-in is defined as user's agreement to try out a new way of completing a task and their willingness to drive and sustain the implementation of a new intervention[15]. HRCH coordinators encouragement, ability to adapt when problems arose was critical to sustaining momentum throughout the implementation phase. Most schools bought into the concept of technology-enabled consent yet their willingness to sustain this change could have benefited from earlier collaboration between immunisation teams, head teachers, and link staff. Parents and student's engagement with the e-consent intervention was variable. Parent interviewees accessed the online portal and were able to complete and submit the e-consent form with ease. However, the significant difference in consent form return rate between e-consent and paper consent schools (73.3% (n=11) vs 91.6% (n=11), p=0.008) response rate (73.3%) indicated that this was not the case for all parents. Of additional concern was that the e-consent intervention inadvertently bypassed some adolescents' information needs and related opportunity to talk to their parents about HPV vaccination. Collective action was evidenced when immunisation teams and schools worked together to make the follow-up of e-consent form non-response more targeted. Reflexive monitoring was facilitated by this real-time evaluation although more co-production activities with HRCH staff, parents, school link staff and adolescents need to be integrated in subsequent evaluations[17, 18].

There is a need to streamline consent processes for adolescent immunisation and ensure that parents and adolescents are fully informed about these preventative measures. Electronic consent

For peer review only - http://bmjopen.bmj.com/site/about/guidelines.xhtml

For peer review only

could be an effective option to achieve this. However, it needs to be tailored to specific contexts, and parents, schools and adolescents need to provide input in the development, implementation and evaluation of such technological interventions. Evaluations also need to factor in the time needed for new working practices to be fully integrated. Our experience suggests that to gain a complete and accurate assessment of the impact of new interventions evaluations need to collect data over more than one cycle of implementation. Introducing change that affects different actors requires all stakeholders to understand, buy in and work together in refining and co-producing complex behavioural interventions.

Acknowledgements

We are grateful for the information shared freely by interview participants, schools and the Hounslow and Richmond Community Healthcare NHS Trust.

Author Contributors

TC and SMJ designed the study with input from SR, EE, HN, ME and RC. EP led the quantitative data analysis with support from RC and SB. TC collected and led the analysis of the qualitative data with support from SB. All authors discussed the preliminary findings and contributed to and critically reviewed the manuscript.

Funding

The research was funded by the National Institute for Health Research Health Protection Research Unit (NIHR HPRU) in Immunisation (Grant reference: HPRU-2012-10096) at the London School of Hygiene and Tropical Medicine in partnership with Public Health England (PHE). The views expressed are those of the author(s) and not necessarily those of the NHS, the NIHR, the Department of Health or Public Health England.

For peer review only

For peer review only - http://bmjopen.bmj.com/site/about/guidelines.xhtml

Competing interests

Tracey Chantler, Sadie Bell, and Sandra Mounier-Jack report that they were in receipt of funding from the National Institute of Health Research while conducting this research. Michael Edelstein, Rosie Cooper and Ellen Pringle worked for Public Health England for the duration of this research.

Ethics approval

The research published in this manuscript was approved by the PHE Research Support & Governance Office (Ref: NR0131) and the London School of Hygiene and Tropical Medicine Observational/Interventions Research Ethics Committee (Ref: 15839). Participants who took part in the qualitative component of this research received study information letters and were provided the opportunity to ask questions before they agreed to take part. Written informed consent was obtained from all research participants prior to interviews (individual, peer-group, parent adolescent dyad) and the FGD. For the latter parental and adolescent informed consent was obtained and school or immunisation staff were not present during the discussion. All participants were told that their participation was voluntary, and their data was stored and presented in a manner that seeks to protect their confidentiality.

Permission for publication

Not applicable

Data availability statement

Data collection tools are provided as supplementary documents and some anonymised data is available on request via the LSHTM public repository.

For peer review only - http://bmjopen.bmj.com/site/about/guidelines.xhtml

References

1. Lu, B., et al., *Efficacy and safety of prophylactic vaccines against cervical HPV infection and diseases among women: a systematic review & meta-analysis.* BMC infectious diseases, 2011. **11**: p. 13-13.
2. Gellin, B., et al., *Quadrivalent Human Papillomavirus Vaccine.* Clinical Infectious Diseases, 2007. **45**(5): p. 609-617.
3. Paul, P. and A. Fabio, *Literature review of HPV vaccine delivery strategies: Considerations for school- and non-school based immunization program.* Vaccine, 2014. **32**(3): p. 320-326.
4. Paterson, P., et al., *Strengthening HPV vaccination delivery: findings from a qualitative service evaluation of the adolescent girls' HPV vaccination programme in England.* Journal of Public Health, 2019.
5. Bentley C, *Tackling gaps and inequalities in girls' uptake of the current HPV vaccination programme. Available at: http://emhf.org/wp-content/ uploads/2015/10/EMHF-HPV-Symposium-April-2015- REPORT.final_.pdf (last accessed 29 June 2016).* 2015.
6. Ferrer, H.B., et al., *Barriers and facilitators to HPV vaccination of young women in high-income countries: a qualitative systematic review and evidence synthesis.* BMC public health, 2014. **14**: p. 700-700.
7. Batista Ferrer, H., et al., *Barriers and facilitators to uptake of the school-based HPV vaccination programme in an ethnically diverse group of young women.* Journal of public health (Oxford, England), 2016. **38**(3): p. 569-577.
8. Boyce, T. and A. Holmes, *Addressing Health Inequalities in the Delivery of the Human Papillomavirus Vaccination Programme: Examining the Role of the School Nurse.* PLOS ONE, 2012. **7**(9): p. e43416.
9. Brabin, L., et al., *The school nurse, the school and HPV vaccination: A qualitative study of factors affecting HPV vaccine uptake.* Vaccine, 2011. **29**(17): p. 3192-3196.
10. National Information Board, *Personalised Heatlh and Care 2020. Using Data and Technology to Transform Outcomes for Patients and Citizens: A Framework for Action*. 2014, HM Government, NHS: file:///C:/Users/sdbltcha/Downloads/national_information_board_s_personalised_health_and_care_2020_framework.pdf (accessed 16th Jan 2020).
11. Boyatzis, R., *Transforming qualitative information: Thematic analysis and code development*. 1998, Thousand Oaks, CA: Sage
12. Griffith, R., *What is Gillick competence?* Human vaccines & immunotherapeutics, 2016. **12**(1): p. 244-247.
13. Public Health England, *Consent: the green book, chapter 2*, in *The Green Book: Immunisation against infectious disease*. 2013, Public Health England: https://assets.publishing.service.gov.uk/government/uploads/system/uploads/attachment_data/file/144250/Green-Book-Chapter-2-Consent-PDF-77K.pdf (accessed 22nd October 2018).
14. May, C.R., et al., *Development of a theory of implementation and integration: Normalization Process Theory.* Implementation science : IS, 2009. **4**: p. 29-29.
15. Finch, T.L., et al., *Improving the normalization of complex interventions: measure development based on normalization process theory (NoMAD): study protocol.* Implementation Science, 2013. **8**(1): p. 43.
16. Mair, F.S., et al., *Factors that promote or inhibit the implementation of e-health systems: an explanatory systematic review.* Bulletin of the World Health Organization, 2012. **90**(5): p. 357-364.
17. Farr, M., et al., *Implementing online consultations in primary care: a mixed-method evaluation extending normalisation process theory through service co-production.* BMJ Open, 2018. **8**(3): p. e019966.

For peer review only - http://bmjopen.bmj.com/site/about/guidelines.xhtml

18. Osborne, S.P., Z. Radnor, and K. Strokosch, *Co-Production and the Co-Creation of Value in Public Services: A suitable case for treatment?* Public Management Review, 2016. **18**(5): p. 639-653.

Figures Legends

Figure 1: The Electronic consent intervention

Figure 2: Theory of Change for the e-consent intervention

[i] *Paper consent would have been used in the 14 control schools at school and community based catch up clinics.*

All parents in e-consent schools would have received the weblink to the e-consent portal and in most cases will have used this link to complete the consent form. Some students who attended e-consent school catch up clinics did not have a signed e-consent form. In those instances, the nurses would phone parents during clinics or obtain adolescent self-consent if appropriate. In the case of community-based catch up clinics parents/guardians and students would usually attend together, if the e-consent had not been completed nurses would either complete the e-consent form at the clinic with the parent and student, if Wifi/technology was available or complete a paper consent form.

For peer review only - http://bmjopen.bmj.com/site/about/guidelines.xhtml

Figure 1: The Electronic consent intervention

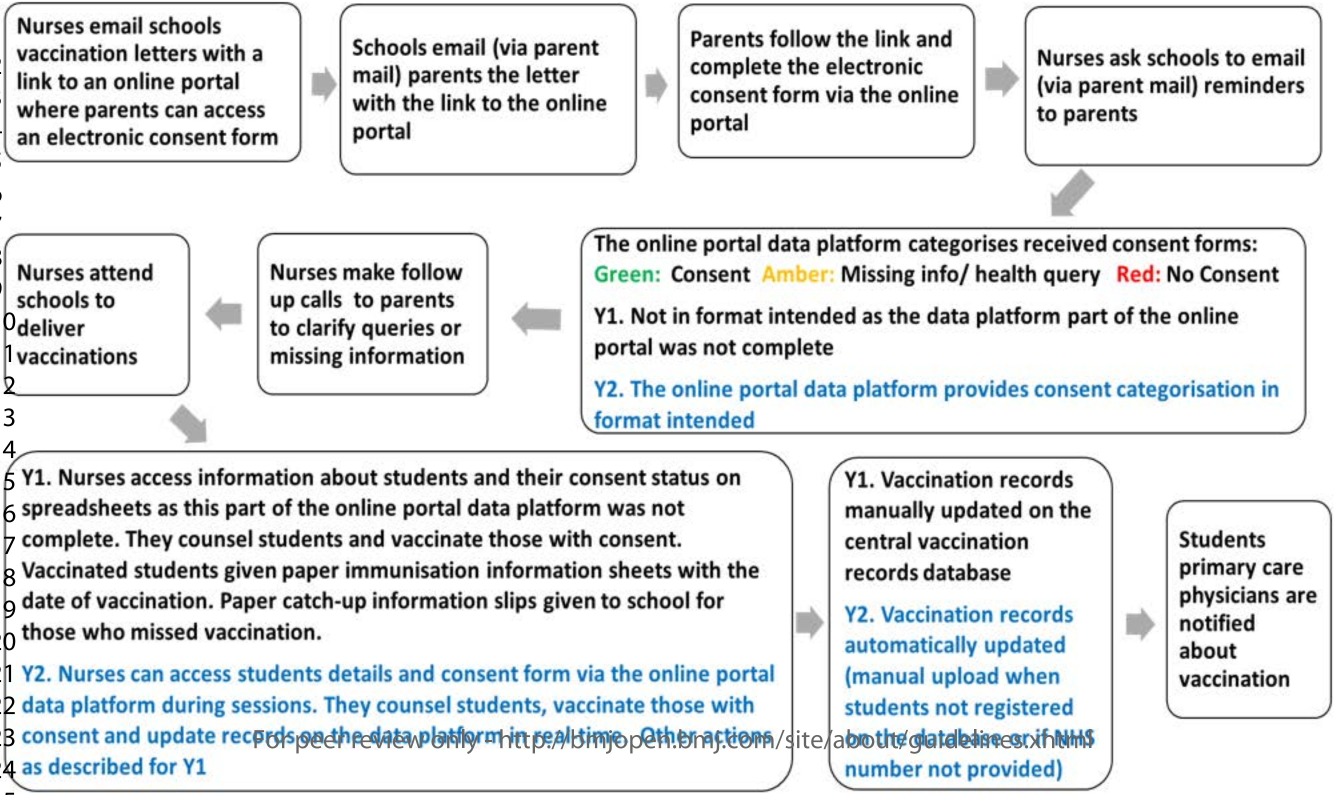

Figure 2: Theory of Change for the e-consent intervention

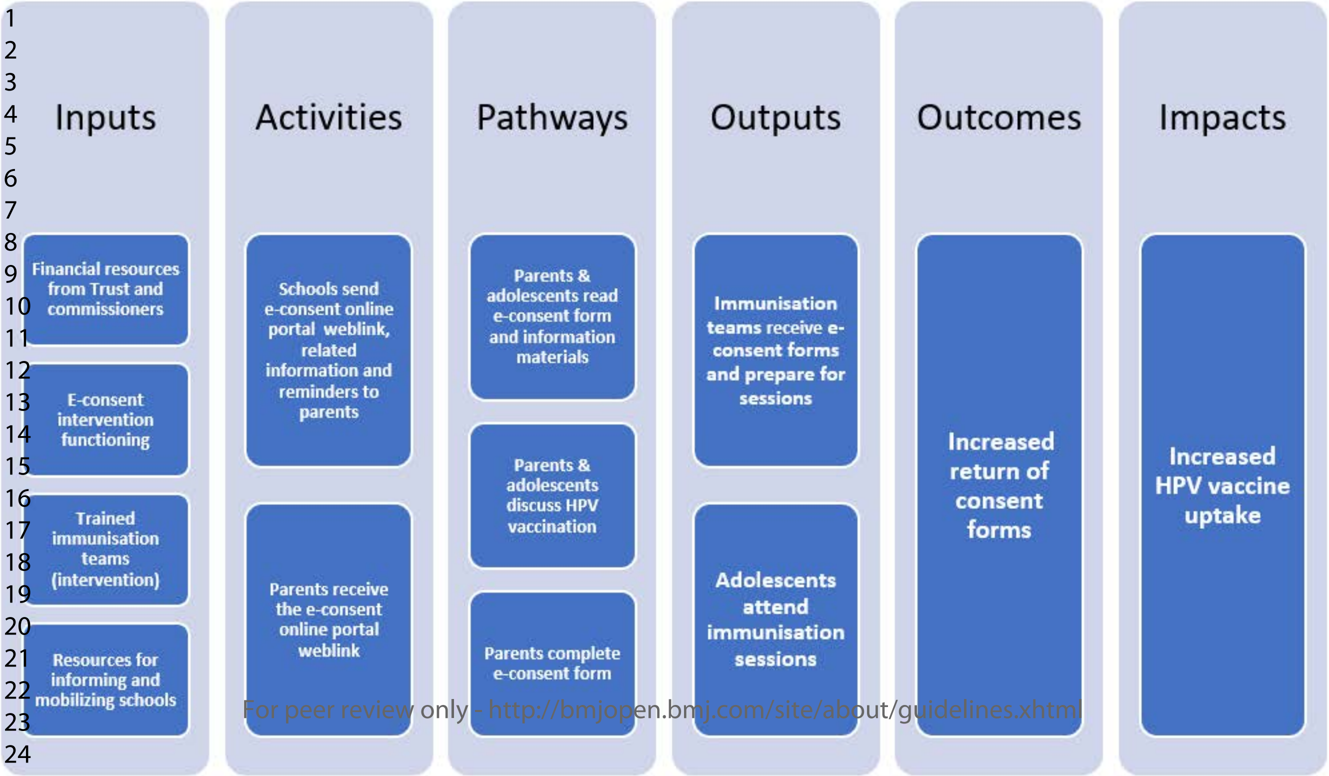

For peer review only - http://bmjopen.bmj.com/site/about/guidelines.xhtml

Additional File 1: School evaluation forms

DOES ELECTRONIC CONSENT IMPROVE THE LOGISTICS AND UPTAKE OF HPV VACCINATION IN ADOLESCENT GIRLS? A MIXED METHODS THEORY INFORMED EVALUATION OF AN INTERVENTION

The research published in this manuscript was approved by the PHE Research Support & Governance Office (Ref: NR0131) and the London School of Hygiene and Tropical Medicine Observational/Interventions Research Ethics Committee (Ref: 15839).

For peer review only

School evaluation form for e-consent schools

Introduction

The Hounslow and Richmond Community Health Trust is in the process of introducing a system that facilitates the collection of electronic consent for school-aged vaccinations. Your school has been involved in using the e-consent system as part of the HPV vaccination programme and we are keen to learn from this experience. We would be very grateful if you could complete this anonymous evaluation form and return it in the attached envelope to a member of the immunisation team at the end of the immunisation session today.

The information that you share with us will help us to evaluate the usability and acceptability of the e-consent system for school-based immunisations and assess its effectiveness in promoting consent form return and HPV vaccine uptake. The findings will guide decision-making about the future use of the e-consent system and help improve the system.

If you have any questions about this please do not hesitate to contact the research team who are based at the London School of Hygiene & Tropical Medicine.

Contacts:

Dr Tracey Chantler
LSHTM, 15-17 Tavistock Place
London, WC1H 9SH
tracey.chantler@lshtm.ac.uk

Sandra Mounier-Jack
LSHTM, 15-17 Tavistock Place
London, WC1H 9SH
sandra.mounier-jack@lshtm.ac.uk

Evaluation form

Part 1

On a scale of 1-10 (1 being poor, 10 being excellent) please rate the following by circling your number of choice. Please add comment to explain your ratings.

a) The organisation of the immunisation session

1	2	3	4	5	6	7	8	9	10
Poor				Average				Excellent	

Comments:

____

____

____

____

b) The usability of the e-consent system for obtaining consent for adolescent vaccination.

1	2	3	4	5	6	7	8	9	10
Poor				Average				Excellent	

Comments:

____

____

____

____

c) The efficiency of the e-consent system (in terms of reducing administrative burden and improving the organisation of immunisation sessions)

1	2	3	4	5	6	7	8	9	10
Poor				Average				Excellent	

Comments:

____

____

____

____

d) Communication between the immunisation team and the school

1	2	3	4	5	6	7	8	9	10
Poor				Average				Excellent	

Comments:

____

____

____

____

For peer review only

Part 2

a) What role have your school staff played in preparing for the HPV immunisation session?

1. Educational activities (*please tick*): Yes ☐ No ☐

If yes please state what these were (e.g. assembly):

____
____
____
____

2. Providing list of eligible students (*please tick*) Yes ☐ No ☐

3. Send reminders to parents (tick all relevant methods):

   a. Parent Mail ☐
   b. Post reminders to home address ☐
   c. Handing out reminders students ☐
   d. Text messages to parents/guardians ☐
   e. Phone call to parents/guardians ☐
   f. Email to parent/guardians with consent form link ☐

4. How many reminders did you send to non-respondents: No: ______________

5. Have you helped parents/adolescents access computers to be able to fill in the form online?

   Yes ☐ No ☐

b) What is your experience of using or facilitating use of the e-consent system? What do you think about obtaining consent for school-based teenage vaccination electronically?

____
____
____
____

c) What have parents and students said about the e-consent system?

____
____
____
____

For peer review only

d) Which means of obtaining consent do you think is more efficient: paper based or e-consent?

____
____
____
____
____

e) How do you think the system could be improved?

____
____
____
____
____
____

Thank you for completing this evaluation form

For peer review only

School evaluation form for paper-based consent schools

Introduction

The Hounslow and Richmond Community Health Trust is in the process of introducing a system that facilitates the collection of electronic consent for school-aged vaccinations. Your school is likely to use this system in the future hence we are keen to learn from your current experience of being involved in coordinating the HPV vaccination programme for adolescent girls. We would be very grateful if you could complete this anonymous evaluation form and return it in the attached envelope to a member of the immunisation team at the end of the immunisation session today.

The information that you share with us will help us to evaluate the usability and acceptability of the e-consent system for school-based immunisations and assess its effectiveness in promoting consent form return and HPV vaccine uptake. The findings will guide decision-making about the future use of the e-consent system and help improve the system.

If you have any questions about this please do not hesitate to contact the research team who are based at the London School of Hygiene & Tropical Medicine.

Contacts:

Dr Tracey Chantler
LSHTM, 15-17 Tavistock Place
London, WC1H 9SH
tracey.chantler@lshtm.ac.uk
07969404745

Sandra Mounier-Jack
LSHTM, 15-17 Tavistock Place
London, WC1H 9SH
sandra.mounier-jack@lshtm.ac.uk
020 7927 2929

Rosie Cooper
Address to be added

rosie.cooper@phe.gov.uk

For peer review only

Evaluation form

Part 1

On a scale of 1-10 (1 being poor, 10 being excellent) please rate the following by circling your number of choice. Please add comments to explain your ratings.

e) The organisation of the immunisation session

1	2	3	4	5	6	7	8	9	10
Poor				Average				Excellent	

Comments:

f) The usability of the current paper-based system for obtaining consent for adolescent vaccination.

1	2	3	4	5	6	7	8	9	10
Poor				Average				Excellent	

Comments:

g) The efficiency of the paper-based system (in terms of administrative burden and organisation of immunisation sessions)

1	2	3	4	5	6	7	8	9	10
Poor				Average				Excellent	

Comments:

h) Communication between the immunisation team and the school

1	2	3	4	5	6	7	8	9	10
Poor				Average				Excellent	

Comments:

For peer review only

Part 2

a) What role have your school staff played in preparing for the HPV immunisation session?

1. Educational activities (*please tick*): Yes ☐ No ☐

   If yes please state what these were (e.g. assembly):

   ____
   ____
   ____
   ____

2. Providing list of eligible students (*please tick*) Yes ☐ No ☐

3. Send reminders/consent forms to parents (tick all relevant methods):

   a. Parent Mail ☐

   b. Post reminders/consent forms to home address ☐

   c. Handing out reminders/consent forms to students ☐

   d. Text messages to parents/guardians ☐

   e. Phone call to parents/guardians ☐

   f. Email to parent/guardians with consent form attached ☐

4. How many reminders did you send to non-respondents: No: _______________

b) What is your experience of using or facilitating use of the existing consent system?

____
____
____
____
____

c) What do you think about obtaining consent for school-based teenage vaccination electronically?

____
____
____
____
____

Thank you for completing this evaluation form

For peer review only

Additional File 2: Interview Topic Guides

DOES ELECTRONIC CONSENT IMPROVE THE LOGISTICS AND UPTAKE OF HPV VACCINATION IN ADOLESCENT GIRLS? A MIXED METHODS THEORY INFORMED EVALUATION OF AN INTERVENTION

The research published in this manuscript was approved by the PHE Research Support & Governance Office (Ref: NR0131) and the London School of Hygiene and Tropical Medicine Observational/Interventions Research Ethics Committee (Ref: 15839).

Interviewees: Immunisation team members

Experience of obtaining consent in school-based vaccination programmes

- Could you tell me about your experience of obtaining consent from parents and adolescents for school-aged vaccinations?
  - What has worked well and what has been challenging?
  - How have you overcome challenges in the past?

Acceptability of the e-consent system

- What were your initial thoughts about the e-consent system? (Probes: concerns, hopes, expectations)
- What did your colleagues think about the system?
- How acceptable do you think the system is for parents, teenagers, and school staff involved in the programme?

Usability of the e-consent system

- How helpful was the training in preparing you to use the e-consent system?
- What was your experience of using the e-consent system? What worked well, what was difficult in the following stages?
  - Triage of consent forms
  - Use of the system during immunisation sessions in schools
  - Uploading data to Trust databases and Child Health Immunisation Systems
  - Any other stages
- What contact did you have with parents about the use of the e-consent system? How did parents find using the e-consent system? What did they find difficult?
- What do you think about how the system may support communication between parents/guardians and adolescents about immunisation? How does this compare with the paper-based system?
- How does the e-consent system support communication between immunisation teams, parents and students? How does this compare with the paper-based system?
- What are the advantages and disadvantaged of the e-consent system in comparison with the paper-based system?

For peer review only

Interaction between schools and immunisation teams

- What is your experience of working with schools in organising immunisation sessions?
- What were schools reactions to the introduction of the e-consent system?
- How has the e-consent system supported interactions between schools and immunisation teams? (Probes: what has worked well, what has been challenging?) How does this compare with the paper-based system?
- What have schools told you about their experience of using the e-consent system? How do they compare it with the paper-based system?
- Have schools conducted any educational activities in preparation for the HPV immunisation visits? What were these? How have you been involved in these?

Adolescent self-consent

- What do you think about giving teen-agers the opportunity to self-consent for vaccination? (Probes: age, what vaccines e.g. HPV)
- Have you obtained self-consent for adolescent immunisation? If yes, in what instances, what was the procedure, and how did you assess for competency?
- How does the e-consent system support self-consent? What is the procedure for this? Did any teen-agers use the e-consent system to give consent for HPV vaccination? What was that experience like?
- Have you had conversations with schools about adolescent self-consent? What are their views?
- Have you had conversations with parents/guardians about adolescent self-consent? What were their views?

Reflections of the use of the e-consent system

- What have been the main lessons in piloting the e-consent system (Probes: what has worked well, what needs to be changed?)
- How would you modify and improve the e-consent system based on the piloting experience?
- What is your advice for other immunisation providers thinking about implementing e-consent?
- Has the e-consent system achieved its intended outcomes from your perspective?

Any other comments

Do you have any other comments?

Thank you for your time.

For peer review only

Interviewees: Service managers

Experience of obtaining consent in school-based vaccination programmes

- Could you tell me about your past experience of managing the logistics of obtaining consent from parents and adolescents for school-aged vaccinations?
  - What has worked well and what has been challenging?
  - How have you overcome challenges in the past?

Decision to launch the e-consent system

- What were the main reasons you decided to implement the e-consent system?
- How did you decide who to collaborate with in developing the e-consent system?
- How was the process of development? What went well, what was difficult?
- How did your test the system prior to the piloting phase?

Acceptability of the e-consent system

- How did your team initially react to the plans to introduce the e-consent system? (Probes: Expectations, concerns, questions)
- Did opinion change over time, and if yes, how?
- How acceptable do you think the e-consent system is for nurses, parents, teenagers, and school staff involved in the programme?

Usability of the e-consent system

- How were you involved in using the e-consent system?
- What was your/your teams' experience of using the e-consent system? What worked well, what was difficult in the following stages?
  - Triage of consent forms
  - Use of the system during immunisation sessions in schools
  - Uploading data to Trust databases and Child Health Immunisation Systems
  - Any other stages
- What contact did you/your team have with parents about the use of the e-consent system? How did parents find using the e-consent system? What did they find difficult?
- What do you think about how the system may support communication between parents/guardians and adolescents about immunisation? How does this compare with the paper-based system?
- How does the e-consent system support communication between immunisation teams, parents and students? How does this compare with the paper-based system?
- What are the advantages and disadvantaged of the e-consent system in comparison with the paper-based system?

For peer review only

Interaction between schools and immunisation teams

- What is your experience of working with schools in organising immunisation sessions?
- What were schools reactions to the introduction of the e-consent system? How did you inform them about the e-consent system, what guidance or training did you provide?
- How has the e-consent system supported interactions between schools and immunisation teams? (Probes: what has worked well, what has been challenging?)
- How does this compare with schools that are using the paper system?
- What have schools told you about their experience of using the e-consent system? How do they compare it with the paper-based system?
- Have schools (those using e-consent and paper system) conducted any educational activities in preparation for the HPV immunisation visits? What were these? How have you/your team been involved in these?

Adolescent self-consent

- Please describe the process you have used for self-consent
- What do you think about giving teenagers the opportunity to self-consent for vaccination? (Probes: age, what vaccines e.g. HPV)
- Have you had conversations with schools about adolescent self-consent? What are their views?
- Have you had conversations with parents/guardians about adolescent self-consent? What were their views?

Reflections of the use of the e-consent system

- What have been the main lessons in piloting the e-consent system (Probes: what has worked well, what needs to be changed?)
- How will you modify and improve the e-consent system based on the piloting experience?
- What have been the costs of implementing the e-consent system?
- What is your advice for other immunisation providers thinking about implementing e-consent?
- Has the e-consent system achieved its intended outcomes from your perspective?

Any other comments

Do you have any other comments?

Thank you for your time.

For peer review only

Interviewees: Service administrators

Experience of managing the administration of the school-based vaccination programmes

- Could you tell me about your past experience of managing the administration of the school-aged vaccination programme?
    - What has worked well and what has been challenging?
    - How have you overcome challenges in the past?

Decision to launch the e-consent system (for senior administrators)

- What were the main reasons the Trust decided to implement the e-consent system?
- How was the process of development? What went well, what was difficult?
- How did your test the system prior to the piloting phase?

Acceptability of the e-consent system

- What were your initial thoughts about the plans to introduce the e-consent system? (Probes: Expectations, concerns, questions)
- Did your opinion change over time, and if yes, how?
- What is your current view on the acceptability of the e-consent system?

Usability of the e-consent system

- How are you involved in using the e-consent system?
- What was your/your teams' experience of using the e-consent system? What worked well, what was difficult in the following stages?
    - Administration of data from the consent forms
    - Administration of data from school and catch-up immunisation sessions
    - Uploading data to Trust databases and Child Health Immunisation Systems
    - Sharing data with GPs
    - Any other stages
- What are the advantages and disadvantaged of the e-consent system in comparison with the paper-based system?

Interaction between schools and immunisation teams

- What role do you play in collaborating with schools to organising immunisation sessions?
- What were schools reactions to the introduction of the e-consent system? How were they informed about the e-consent system, what guidance or training did they receive?
- How has the e-consent system supported interactions between schools and immunisation providers? (Probes: what has worked well, what has been challenging?)
- How does this compare with schools that are using the paper system?
- What have schools told you about their experience of using the e-consent system? How do they compare it with the paper-based system?

For peer review only

Reflections of the use of the e-consent system

- What have been the main lessons in piloting the e-consent system (Probes: what has worked well, what needs to be changed?)
- How will you modify and improve the e-consent system based on the piloting experience?
- What have been the costs of implementing the e-consent system?
- What is your advice for other immunisation providers thinking about implementing e-consent?
- Has the e-consent system achieved its intended outcomes from your perspective?

Any other comments

Do you have any other comments?

Thank you for your time.

For peer review only

Interviewees: Parents/guardians

Views on adolescent vaccination

- What do you know about the vaccines that are offered to teenagers? (Probes: HPV, teenage booster, Men ACWY)
- What information have you received about teenage vaccinations? Who gave you this information? Did you search for additional information, if yes where did you look and what did you find?
- What is your view about adolescent vaccination? Are there any vaccines that you are concerned about?

Experience of providing consent for adolescent vaccinations

- Could you tell me about your experience of providing consent for your teenager(s) to be vaccinated?
- How did you make your decision about providing consent?
    - Who else did you talk to about this? What did you discuss? What were their views? (Probes: Partner, teenager, other family members, friends, health professionals)
- How did you involve your teenager in this decision-making? Were you in agreement about having the vaccine?
- Have you ever changed your mind about providing consent for a vaccination, if yes why and what did you do?

Usability of the e-consent system

- How did you find using the e-consent system? Who completed the form?
- Was it easy to access the e-consent site via the email sent from school?
- What was you experience of completing the consent form online? Were there any parts that you found difficult to complete? How did you know if you had completed the form properly?
- What was your experience of accessing the HPV vaccination programme leaflets via the e-consent system? Did you read these online or did you print them out? Did you share these leaflets with your teenager or other members of the family?
- Did you contact the immunisation team for help to complete the e-consent form? What support did you require? How easy was it to contact them?
- How do you think that the e-consent form could be improved?

Acceptability of the e-consent system

- What are your views on giving consent electronically for your teenagers' vaccinations? (Probes: any concerns about sharing personal data online?)
- What did your teen-ager think about this method of giving consent?
- What are your views on how the e-consent system supports information sharing and communication about teenage vaccination?

For peer review only

Teenagers experience of immunisation in school

- What did your teenager tell you about their experience of being immunised in school? (Probes: what went well, what did they find difficult)
- How well prepared do you think your teenager was prior to the immunisation session? (Probes: access to information, educational sessions in school)

Views on adolescent self-consent

- What do you think about the option of teen-agers providing self-consent for vaccination?
- What is your understanding of self-consent in adolescence?
- Would you be happy for your teen-ager to give self-consent for vaccination? If yes, why. If no, why?

Any other comments

Do you have any other thoughts about adolescent vaccination and how consent is obtained for this?

Thank you for your time.

For peer review only

Interviewees: Adolescents (questions asked with parents)

Views on adolescent vaccination

- What do you know about the vaccines that are offered to teenagers? (Probes: HPV, teenage booster, Men ACWY)
- What information have you received about the HPV vaccination? Who gave you this information? Have you accessed any other information, if yes what was this?
- What is your view about the HPV vaccination? (Probes: what is good about it, any concerns about the vaccine or the process of vaccination)
- Who have you talked to about having the HPV vaccine? (Probes: parents, friends, school nurse, other health professionals, other family members) What did you discuss?

Experience of giving consent for vaccination

- Who decided whether you would receive the HPV vaccine? How did they make this decision?
- How were you involved in this decision-making? Were you in agreement with your parents about this decision?

Usability of the e-consent system

- Were you involved in using the e-consent system? If yes, how were you involved either at home or in school and what did you think about this system?
- What did your parents think about the e-consent system? Were there any parts of the online form that they found difficult to complete?
- Were you given the opportunity to find out more about the HPV vaccine via the e-consent system? (Probes: Access leaflets, read and discuss these with your parents)
- What do your friends think about the e-consent system?
- How do you think that the e-consent system could be improved?

Acceptability of the e-consent system

- What are your views on giving consent online for your vaccinations? (Probes: any concerns about sharing personal data?)
- What are your views on how the e-consent system supports finding out about and talking about teenage vaccination?

Experience of the immunisation session

For peer review only

- Could you tell me a bit about your experience of receiving the HPV vaccine in school? What was the session like, how did you feel, what went well, what was difficult?
- How well prepared were you for the immunisation session? (Probe: know you would be vaccinated against HPV, understood what the vaccine was for any why you needed it)
- Were you given the opportunity to ask any questions before you were vaccinated?
- Had you participated in any information sessions about vaccination at school before you were vaccinated? If yes, what were these and what did you learn?
- Had you received any other information about the HPV vaccine before you were vaccinated? If yes, who gave you this?

Views on adolescent self-consent

- What do you think about the option of teen-agers providing self-consent for vaccination?
- What is your understanding of self-consent?
- Have you ever been given the option to provide self-consent for vaccination?
- Would you be happy to provide self-consent for vaccination? If yes, why. If no, why?

Any other comments

Do you have any other thoughts about vaccination and how consent is obtained for this?

Thank you for your time

For peer review only

Adolescent Focus Group Discussion Topic Guide

Interviewees: Adolescents (Year 8/9 girls)

Views on adolescent vaccination

- What do you know about the vaccines that are offered to teenagers? (Probes: HPV, teenage booster, Men ACWY)
- What information have you received about the HPV vaccination? Who gave you this information? Have you accessed any other information, if yes what was this?
- What is your view about the HPV vaccination? (Probes: what is good about it, any concerns about the vaccine or the process of vaccination)
- Who have you talked to about having the HPV vaccine? (Probes: parents, friends, school nurse, other health professionals, other family members) What did you discuss?

Experience of giving consent for vaccination

- Who decided whether you would receive the HPV vaccine?
- How were you involved in this decision-making? Were you in agreement with your parents about this decision?

Usability of the e-consent system

- Were you involved in using the e-consent system? If yes, how were you involved either at home or in school and what did you think about this system?
- What did your parents think about the e-consent system? Were there any parts of the online form that they found difficult to complete?
- Were you given the opportunity to find out more about the HPV vaccine via the e-consent system? (Probes: Access leaflets, read and discuss these with your parents)
- What do your friends think about the e-consent system?
- How do you think that the e-consent system could be improved?

Acceptability of the e-consent system

- What are your views on giving consent online for your vaccinations? (Probes: any concerns about sharing personal data?)
- What did your parents think about this way of obtaining consent?

Experience of the immunisation session

- Could you tell me a bit about your experience of receiving the HPV vaccine in school? What was the session like, how did you feel, what went well, what was difficult?
- How well prepared were you for the immunisation session? (Probe: know you would be vaccinated against HPV, understood what the vaccine was for any why you needed it)
- Were you given the opportunity to ask any questions before you were vaccinated?

For peer review only

- Had you participated in any information sessions about vaccination at school before you were vaccinated? If yes, what were these and what did you learn?
- Had you received any other information about the HPV vaccine before you were vaccinated? If yes, who gave you this?

Views on adolescent self-consent

- What do you know about the option of teenagers providing self-consent for vaccination?
- Do you know what self-consent means?
- Would you be happy to provide self-consent for vaccination? If yes, why. If no, why?

Any other comments

Do you have any other thoughts about vaccination and how consent is obtained for this?

Thank you for your time.

For peer review only

Good Reporting of A Mixed Methods Study (GRAMMS)

The quality of mixed methods studies in health services research. Alicia O'Cathain, Elizabeth Murphy[1], Jon Nicholl Medical Care Research Unit, School of Health and Related Research, University of Sheffield, Sheffield; [1]School of Sociology and Social Policy, University of Nottingham, Nottingham, UK.

Accessed via: https://www.equator-network.org/reporting-guidelines/the-quality-of-mixed-methods-studies-in-health-services-research/

Applied to:

DOES ELECTRONIC CONSENT IMPROVE THE LOGISTICS AND UPTAKE OF HPV VACCINATION IN ADOLESCENT GIRLS? A MIXED METHODS THEORY INFORMED EVALUATION OF A PILOT INTERVENTION

(1) Describe the justification for using a mixed methods approach to the research question.

The use of a mixed methods theory-informed study design is described and justified in the strengths and limitations summary box (page 3, lines 60-62) and the study design section (page 5, lines 112-120). It is also illustrated in Figure 2. The study design allowed us to measure the effect of a pilot e-consent intervention on immunisation performance and identify mechanisms that facilitated or impeded implementation.

(2) Describe the design in terms of the purpose, priority and sequence of methods.

This is described in the study design section (page 5, lines 112-120); the choice of methods was informed by the need to obtain qualitative data to examine the implementation process and quantitative data to assess outcomes and the impact of the intervention.

(3) Describe each method in terms of sampling, data collection and analysis.

These are described in full in the methods section of the manuscript. The sampling frame for the schools participating in the evaluation is detailed in the selection of schools. The boundaries sampling of interviews participants for the qualitative methods were set by this selection. We interviewed all the staff who were actively involved in the implementation process and observed immunisation sessions at schools in each of the boroughs that piloted the intervention. The recruitment and sampling of parent and adolescent interviewees (individual and paired parent/adolescent) is outlined in lines 182-188. We were only able to recruit parents and by proxy adolescents who had ticked a box on the e-consent portal stating they were willing to be interviewed. Informed consent was obtained before all qualitative interviews as described in the ethics section at the end of the manuscript.

(4) Describe where integration has occurred, how it has occurred and who has participated in it.

For peer review only

The study team (co-authors) met every 3-4 months to discuss the results of the evaluation and compare quantitative with qualitative findings. The qualitative findings were pivotal for understanding why the pilot intervention did not achieve the expected improvement to consent form return in Year 1 and to identify mechanisms that facilitated or impeded the implementation of the intervention. Collaboration between implementers and evaluators in this real time evaluation also supported the iterative development of the pilot intervention, which is critical to the longer-term integration of new working practices.

(5) Describe any limitation of one method associated with the present of the other method.

I am not completely clear what is meant by this question. The methods complemented each other and were not limited by the presence of another method. The observations informed the interviews and findings from the qualitative data provided insights into implementation challenges which in part explained quantitative findings.

(6) Describe any insights gained from mixing or integrating methods.

As stated in point one the use of diverse methods allowed us to examine the implementation of the pilot intervention and assess its effect on consent form returns (outcomes) and HPV vaccine uptake (impact). Using qualitative methods over a period of a year was very useful to trace how the intervention was adapted over time to improve its acceptability and usability. The design of our evaluation could have been improved if we had been able to repeat the quantitative data collection in Year 2. This was not possible since the new iteration of the intervention was not ready when the original sample of e-consent schools were scheduled to receive the HPV vaccine in Year2. This is a constraint of evaluating interventions in real-time.

For peer review only

BMJ Open

DOES ELECTRONIC CONSENT IMPROVE THE LOGISTICS AND UPTAKE OF HPV VACCINATION IN ADOLESCENT GIRLS? A MIXED METHODS THEORY INFORMED EVALUATION OF A PILOT INTERVENTION

Journal:	*BMJ Open*
Manuscript ID	bmjopen-2020-038963.R2
Article Type:	Original research
Date Submitted by the Author:	10-Sep-2020
Complete List of Authors:	Chantler, Tracey; London School of Hygiene & Tropical Medicine, Global Health & Development<br>Pringle, Ellen; Public Health England, Department of Immunisation, Hepatitis and Blood Safety, Public Health England, UK<br>Bell, Sadie; London School of Hygiene and Tropical Medicine Faculty of Public Health and Policy, Global Health and Development<br>Cooper, Rosie; University Hospitals of Derby and Burton NHS Foundation Trust<br>Edmundson, Emily; Hounslow and Richmond Community Healthcare NHS Trust<br>Nielsen, Heidi; Hounslow and Richmond Community Healthcare NHS Trust<br>Roberts, Sheila; Hounslow and Richmond Community Healthcare NHS Trust<br>Edelstein, Michael; Public Health England, Immunisation and countermeasures division<br>Mounier-Jack, Sandra; London School of Hygiene and Tropical Medicine,
**Primary Subject Heading**:	Public health
Secondary Subject Heading:	Health services research
Keywords:	Public health < INFECTIOUS DISEASES, Information technology < BIOTECHNOLOGY & BIOINFORMATICS, Paediatric infectious disease & immunisation < PAEDIATRICS

SCHOLARONE™ Manuscripts

I, the Submitting Author has the right to grant and does grant on behalf of all authors of the Work (as defined in the below author licence), an exclusive licence and/or a non-exclusive licence for contributions from authors who are: i) UK Crown employees; ii) where BMJ has agreed a CC-BY licence shall apply, and/or iii) in accordance with the terms applicable for US Federal Government officers or employees acting as part of their official duties; on a worldwide, perpetual, irrevocable, royalty-free basis to BMJ Publishing Group Ltd ("BMJ") its licensees and where the relevant Journal is co-owned by BMJ to the co-owners of the Journal, to publish the Work in this journal and any other BMJ products and to exploit all rights, as set out in our licence.

The Submitting Author accepts and understands that any supply made under these terms is made by BMJ to the Submitting Author unless you are acting as an employee on behalf of your employer or a postgraduate student of an affiliated institution which is paying any applicable article publishing charge ("APC") for Open Access articles. Where the Submitting Author wishes to make the Work available on an Open Access basis (and intends to pay the relevant APC), the terms of reuse of such Open Access shall be governed by a Creative Commons licence – details of these licences and which Creative Commons licence will apply to this Work are set out in our licence referred to above.

Other than as permitted in any relevant BMJ Author's Self Archiving Policies, I confirm this Work has not been accepted for publication elsewhere, is not being considered for publication elsewhere and does not duplicate material already published. I confirm all authors consent to publication of this Work and authorise the granting of this licence.

For peer review only

DOES ELECTRONIC CONSENT IMPROVE THE LOGISTICS AND UPTAKE OF HPV VACCINATION IN ADOLESCENT GIRLS? A MIXED METHODS THEORY INFORMED EVALUATION OF A PILOT INTERVENTION

Authors

Tracey Chantler[1*§], Ellen Pringle[*2]

Sadie Bell[1], Rosie Cooper[3], Emily Edmundson[4], Heidi Nielsen[4], Sheila Roberts[4], Michael Edelstein[1,2], Sandra Mounier-Jack[1]

1. Department of Global Health and Development, Faculty of Public Health and Policy, London School of Hygiene & Tropical Medicine, 15-17 Tavistock Place, London, WC1H 9SH, UK
2. Department of Immunisation, Hepatitis and Blood Safety, Public Health England, UK
3. University Hospitals of Derby and Burton NHS Foundation Trust, Uttoxeter Road, Derby, DE22 3NE
4. Hounslow Richmond Community Healthcare NHS Trust, 18 High Street, Teddington, London, TW118HU, UK

*Joint first authors

§Corresponding author: Tracey Chantler, Department of Global Health and Development, Faculty of Public Health and Policy, London School of Hygiene & Tropical Medicine, 15-17 Tavistock Place, London, WC1H 9SH, UK, tracey.chantler@lshtm.ac.uk Tel: 07969404745

Word count: 4168

1

For peer review only

Abstract

Objectives

To evaluate the usability and acceptability of an electronic consent pilot intervention for school-based immunisations and assess its impact on consent form returns and HPV vaccine uptake.

Design

Mixed-methods theory-informed study applying qualitative methods to examine the usability and acceptability of the intervention and quantitative methods to assess its impact.

Setting and participants

The intervention was piloted in 14 secondary schools in seven London boroughs in 2018. Intervention schools were matched with schools using paper consent based on the proportion of students with English as a second language and students receiving free school meals. Participants included nurses, data managers, school-link staff, parents and adolescents.

Interventions

An electronic consent portal where parents could record whether they agreed or declined vaccination and nurses could access data to help them manage the immunisation programme.

Primary and Secondary Outcome Measures

Comparison of consent form return rates and HPV vaccine uptake between intervention and matched schools.

Results

HPV vaccination uptake did not differ between intervention and matched schools, but timely consent form return was significantly lower in intervention schools (73.3% vs 91.6%, p=0.008). The transition to using electronic consent was not straightforward, whilst schools and staff understood the potential benefits, they found it difficult to adapt to new ways of working which removed some level of control from schools. Reasons for lower consent form return in e-consent schools included

For peer review only

difficulties encountered by some parents in accessing and using the intervention. Adolescents highlighted the potential for electronic consent to by-pass their information needs.

Conclusions:

The pilot intervention did not improve consent form return or vaccine uptake due to challenges encountered in transitioning to new working practice. New technologies require embedding before they become incorporated in everyday practice. A re-evaluation once stakeholders are accustomed with electronic consent may be required to understand its impact.

Keywords

Public health, Information technology, Paediatric infectious disease and immunisation

Strengths and limitations

- The use of a theory-informed mixed methods study design allowed us to measure the effect of a pilot e-consent intervention on immunisation performance and identify mechanisms that facilitated or impeded implementation.
- The study design allowed us to account for schools, nurses, data managers, parents and adolescents' experiences of using the e-consent technology in this evaluation.
- Data limitations include the lack of interviews with school staff to complement the feedback forms and not being able to access and interview parents who found the intervention more challenging to use.
- This evaluation was limited to one cycle of implementation, which did not allow us to account for the time it takes for new practices to become embedded. The number of schools who declined to pilot the intervention was also not recorded.

For peer review only

Introduction

Vaccination against human papilloma virus (HPV) provides long-term protection against cervical and other cancers and genital warts[1, 2]. In England HPV vaccination (2 doses 6 months apart) is offered to adolescents aged 12-14 years in the school-based immunisation programme, which is delivered by nurse led immunisation teams. Whilst the school delivery model is widely accepted[3], it raises logistical challenges regarding obtaining parental consent. Immunisation teams and schools identify all students, who are eligible for vaccination and then students are typically given a paper consent form that their parents/legal guardians need to sign indicating their acceptance or refusal of vaccination. The passage of the form from teachers to students to parents and back again can impact on form return and have a detrimental effect on vaccine uptake[4-7]. Follow-up by school staff and immunisation teams improves uptake but is resource intensive[8, 9]. In the context of the drive towards a "paperless NHS"[10], there is increasing interest in the potential for technological solutions, such as electronic consent (e-consent). We evaluated the usability and acceptability of a pilot e-consent intervention from the perspective of parents and adolescents, health professionals and schools, and assessed its impact on consent form returns and HPV vaccine uptake.

The electronic consent intervention

The e-consent intervention was developed by Hounslow and Richmond Community Healthcare NHS Trust (HRCH) with the support of a software development company in 2017/18 and piloted in their adolescent girls' vaccination programme in June/July 2018. At the time of the study HRCH was responsible for administering this vaccination programme in secondary schools across eight boroughs in South London.

The e-consent intervention consisted of an online portal with an e-consent form and a data platform and related implementation procedures (Figure 1). Functionally it aimed to: 1) give parents access

For peer review only - http://bmjopen.bmj.com/site/about/guidelines.xhtml

to an online portal with information about the vaccination programme where they could register their adolescent and agree to or decline HPV vaccination; 2) give nurses electronic access to the portal to facilitate screening and enable them to update records during immunisation sessions; 3) enable automatic updating of central vaccination record databases. Parts of the online portal and data platform (specifically those relating to points 2 & 3) were not fully functioning before the intervention was first used in June 2018. Hence modifications had to be made to the way nurses screened students' information and consent forms before and during immunisation sessions. Figure 1 differentiates what happened in Year 1: June/July 2018 and in Year 2: June/July 2019 (blue text). In the quantitative part of evaluation, we focused on Year 1; the measurement of outcomes and impacts. In the qualitative part of the evaluation we captured how Year 1 experience informed adaptations to the intervention prior to reuse in Year 2.

Methods

Study design

This was a mixed-methods theory-informed evaluation study which used a 'Theory of Change' (Figure 2) as an evaluation framework. Quantitative methods were used to assess whether the pilot intervention increased consent form return and the uptake of the first dose of HPV vaccine in adolescent girls in June/July 2018 (Year 1). The steps involved in implementing the e-consent system, people's experiences of these, the interactions between inputs, activities, pathways and outputs, outcomes and impacts were investigated using qualitative methods in Year 1 and Year 2. The reason that the quantitative analysis only focused on Year 1 was that the schools that received the intervention between Year 1 & 2 changed.

For peer review only - http://bmjopen.bmj.com/site/about/guidelines.xhtml

School selection

The e-consent intervention was piloted in 14 secondary schools in seven South London boroughs in June/July 2018 (Year 1). Schools selection was purposive with the aim of including schools that differed in terms of denomination (private, state, grammar), type (mixed, single sex), socio demographic, size, vaccination uptake, and level of support to the programme.

Schools were divided into low, medium or high based on the proportion of pupils receiving free school meals and with English as an additional language. Each e-consent school was matched, as closely as possible, to a paper consent school in the same tertiles for both characteristics.

Quantitative methods & analysis

Data collection (Year 1)

At each vaccination session nurses completed a "tally sheet", with details of the consents received prior to or during the session, any absences and the number of vaccinations given. These tally sheets were completed for paper and e-consent schools.

In addition, we extracted the name of the school, date and time of consent form completion and type of consent (agreement by parent or adolescent self-consent or decline) for each consent form in the e-consent system. This non-identifiable information was combined with tally sheet data, which was manually transcribed into MS Excel. Where there were discrepancies or missing data this was checked with the immunisation teams and in the case of the e-consent schools the system data was used in preference to the tally sheet data.

Data analysis

Characteristics of the paper and e-consent schools were described in terms of proportion of pupils receiving free school meals, speaking English as an additional language, ethnicity and characteristics of the schools (religious affiliation and state/privately funded), using data from the Office of National

For peer review only - http://bmjopen.bmj.com/site/about/guidelines.xhtml

Statistics. (REF: https://www.gov.uk/government/statistics/schools-pupils-and-their-characteristics-january-2018).

For both paper and e-consent schools, we calculated: (i) the proportion of the pupils for whom a consent form had not been returned prior to the vaccination session, (ii) the proportion of pupils vaccinated at the planned session and (iii) the proportion of pupils for who a “yes” consent was received (prior to or on the day of the planned vaccination session).

We compared e-consent schools with paper consent schools in terms of timely form return, outcome of consent and vaccination uptake using Kruskall Wallis tests for statistical significance. Where data was missing from a school that school and its matched pair were excluded from the analysis. All analyses were done in MS Excel and Stata 15.

Qualitative methods & analysis

Data collection

Data collection occurred in June-December 2018 (Year 1) and June-July 2019 (Year 2). The first period coincided with and followed the first year of e-consent implementation, the second occurred during the use of e-consent in a different subset of schools.

June-July 2018 (Year 1)

Observations of e-consent school HPV immunisation sessions

Members of the evaluation team (RC, TC, SMJ) accompanied immunisation teams during seven e-consent school HPV immunisation sessions to obtain a contextual understanding of the implementation of the intervention. The evaluators documented what worked well and what if any problem-solving was applied during sessions.

School feedback forms from paper and e-consent schools

For peer review only

For peer review only - http://bmjopen.bmj.com/site/about/guidelines.xhtml

School staff involved in organising immunisation sessions were asked to complete a feedback form after the HPV immunisation sessions (Additional file 1). Forms included questions about the organisation of immunisation sessions and the usability and acceptability of e-consent and paper consent. Seven e-consent and seven paper consent schools completed forms.

Individual and peer group interviews with HRCH staff

These semi-structured interviews (SSI's) involved HRCH staff who were responsible for implementing the intervention. The programme manager was interviewed individually, the two data specialists together and member of four immunisation teams in peer-groups of two to four participants (12 interviewees) at their respective offices. In total 15 members of staff took part in hour long interviews.

Interviews with parents and adolescents from e-consent schools

These SSIs involved parents and adolescents who had used the e-consent system. Parents/legal guardians were asked to indicate their willingness to be contacted by researchers in the e-consent form (ticked a statement). Parents who responded positively were invited by email to take part in an interview to discuss their experience of using the e-consent system. A total of 12 interviews were conducted, nine with HPV vaccine acceptors and three with decliners. Four interviews were conducted in family homes, seven by telephone, and one by Skype video. Adolescents participated in five interviews.

June-July 2019 (Year 2)

Results from data collected in Year 1 informed the implementation of the e-consent intervention in 22 schools located in South London in the 2018/19 HPV vaccine programme. Only one of these schools had taken part in the 2018 pilot, the reasons for using the improved intervention in this school and not the other pilot schools was mainly due to the scheduling of the HPV vaccination sessions. The Trust also wanted to expose additional schools to the e-consent system. To reflect the

For peer review only - http://bmjopen.bmj.com/site/about/guidelines.xhtml

changes made to the intervention and its implementation two group interviews with HRCH staff (one with nine and the other with five members of staff) and one focus group discussion (FGD) with eight adolescent girls (from a school that used the e-consent intervention in 2019 only) were conducted.

The topics covered in the staff, parent and adolescent SSIs and adolescent FGD are summarised in Text Box 1 (see Additional File 2 for Topic Guides).

Text box 1: Topics covered in interview

Staff interviews	Parent and adolescent interviews
• Experience of managing the administration of the school-based vaccination programme<br>• Experience of obtaining consent in school-based vaccination programme<br>• Acceptability and usability of the e-consent system<br>• Interaction between schools and immunisation teams<br>• Adolescent self-consent<br>• Reflections on use of the e-consent system	• Views on adolescent vaccination<br>• Understanding of adolescent vaccination<br>• Experience of providing consent for the HPV vaccine<br>• Acceptability and usability of the e-consent system<br>• Teenagers experience of immunisation in school<br>• Vaccine programme communication<br>• Views on adolescent self-consent

Data analysis

The SSIs and FGD were audio recorded with participant’s permission and transcribed anonymously. Transcripts, observational field notes and school feedback forms were downloaded into a qualitative data analysis management software programme (NVivo 12). We adopted a thematic analytical approach which combined semi-deductive mapping of data to the ‘input’, ‘activities’, ‘pathways’, ‘outputs’, ‘outcomes’ and ‘impacts’ depicted in the ToC and inductive open coding to capture emerging themes[11]. We sought to account for the interdependence of ToC categories and real-life experience of managing organisational change, which do not always progress from inputs – impacts in a seamless linear manner.

For peer review only

For peer review only - http://bmjopen.bmj.com/site/about/guidelines.xhtml

Patient and public involvement

This evaluation was designed with members of HRCH who were responsible for coordinating the implementation of the e-consent intervention. This collaboration supported the iterative and reflexive development of the intervention, which is essential for longer-term integration.

Results

Quantitative

Participants

Twenty-eight schools (14 paper and 14 e-consent schools) comprising 3219 girls (1733 in paper consent and 1486 in e-consent schools) were included in the study. Of those schools, 26 were state and 2 were private schools. Twenty-one of the schools had no religious affiliation, three were Roman Catholic (all paper consent), three Church of England (two e-consent, one paper consent) and one was another Christian faith school (paper consent).

The proportion of pupils eligible for free school meals, with English as an additional language and students' ethnicity profile was similar between the e-consent and paper consent schools (Table 1).

Table 1: Characteristics of paper and e-consent schools[1,2]

	Paper consent schools (n=13)	E-consent schools (n=13)	P-value*
**% (range) of children eligible for free school meals**	17.3 (1.5-52.1)	17.3 (1.5-43.2)	0.84
**% (range) of children with English as additional language**	30.9 (5.5-53.6)	32.4 (9.1-59.2)	0.72
**% (range) of children of white British ethnicity**	30.9 (1.5-69.6)	34.6 (6.3-65.7)	0.63

*Refers to comparison of each characteristic using Kruskal-Wallis test

1. Characteristics information was not available for the two private schools
2. Data from Schools, pupils and their characteristics: January 2018, Department of Education

For peer review only

For peer review only - http://bmjopen.bmj.com/site/about/guidelines.xhtml

Return of consent forms ahead of session

Overall, 83% of consent forms (paper or e-consent) were returned prior to the vaccination session. However, amongst the 22 matched schools where this data was available, compared with paper schools timely (prior to the planned session) return was lower in the e-consent schools (73.3% vs 91.6%, p=0.008).

Outcome of consent

There was no statistically significant difference in the proportion of pupils for whom a “yes” consent was received (prior to or on the day of the session) between the paper (n=14) and e-consent (n=14) schools (85% in e-consent schools, 83% in paper consent schools, p=0.89).

Vaccination uptake

There was no statistically significant difference in the proportion of pupils that were vaccinated at the scheduled vaccination session between the paper (n=14) and e-consent (n=14) schools (80.6% vs 81.3%, p=0.93). These figures did not include those who were absent on the day and vaccinated later[i]. The final vaccine uptake across all the schools was over 86%.

Qualitative

The results of the ToC thematic analytical mapping are presented under relevant headings (‘input’, ‘activities’, ‘pathways’, ‘outputs’, ‘outcomes’) followed by an overarching theme on managing change.

Inputs

Resources and Training

There was a *‘buzz’* about the development of the e-consent intervention, its potential to streamline consent and facilitate safer data collection and NHS England had provided funding for HRCH to pilot it during the 2017/18 HPV vaccine programme. Immunisation team members were positive but

For peer review only - http://bmjopen.bmj.com/site/about/guidelines.xhtml

expressed reservations about not able to review paper consent forms prior to immunisation. Due to tight deadlines only one orientation session took place before the e-consent intervention was introduced, which meant that the bulk of learning happened on the job.

"I think as well, it was probably four days before our first session, we didn't know what we were doing...so I do feel we are running before we can walk." (Immunisation Team 2)

Intervention not fully operational

The data platform component of the online portal was not operational prior to implementation. Parents were able to access and complete the e-consent form, but the immunisation teams could not review student's online consent forms or upload data during immunisation sessions. Instead large (A3) paper sheets with information about who had provided consent were prepared by the data managers. The sheets were difficult for to decipher during busy sessions and nurses were less able to prepare cohort figures and tally sheets in advance.

"It was an anti-climax not being able to use the laptops and still have a paper sheet in front of me." (Immunisation Team 4)

Mobilizing and resourcing schools

There was limited time to collaborate with schools before the vaccination programme, although all e-consent schools were guided on how to disseminate the weblink. A few schools in 2 inner city boroughs declined to use the intervention due to concerns about: i) pre-existing barriers to electronic communication with parents, ii) whether a change to consent processes would reduce the return of consent forms and uptake of HPV vaccine, iii) adapting to a new way of working.

School immunisation link staff reported a *'loss of control'*, for example they could no longer see *'who had said yes, and who had said no'*, which restricted their ability to follow-up unreturned forms. Paper-consent schools could monitor this directly by counting forms, but with e-consent schools

For peer review only - http://bmjopen.bmj.com/site/about/guidelines.xhtml

immunisation teams had to check parental responses and tell schools which families had not replied.

Activities

Dissemination of the online e-consent portal weblink to parents

E-consent schools used different means (parent mail, email, school website, newsletters, letters) to send parents the portal weblink. Blanket reminders were mainly sent electronically, unless immunisation teams provided schools with details of non-responders. In this case follow-up could be more targeted and involve text messages and phone calls as wells as emails. One school used a translator to engage parents who did not understand the consent process due to language barriers. Another school was not willing to send out emails and asked the immunisation team to provide them with printed letters referring to the weblink to send to parents.

Of the seven e-consent schools who completed the feedback form, four were positive about the intervention and how it had been implemented stating that it had reduced their workload. Another school was mainly positive but noted that some parents had found the e-consent form difficult to access, another reported that their parent cohort had found the system very difficult to access and use, and the last school was the one who had used letters to disseminate the weblink.

Pathways

Navigating the e-consent form and related information

The e-consent form included links to an HPV vaccination leaflet. However, none of the interviewees had downloaded or read this leaflet for the following reasons: accessed information elsewhere, already sufficiently informed, older daughter vaccinated, positive about vaccination.

Proactive information seeking involving a wider range of sources was more common in families who were vaccine hesitant. Their concerns, which in some cases resulted in vaccine refusal, included the following: i) compatibility of vaccination with pre-existing illness, ii) unknown side-effects (e.g.

For peer review only - http://bmjopen.bmj.com/site/about/guidelines.xhtml

hormonal interferences while daughter establishing her cycle), iii) targeting of adolescents due to their ethnicity (nurses explained that some parents of Black-Caribbean origin were sceptical of any state intervention, like vaccination), iv) necessity of vaccination for adolescents who are not sexually active. Parents who were more confident about vaccines restricted their information seeking to NHS sources and suggested that a 'road map' to adolescent vaccination could be useful.

Adolescents reported a variety of information seeking behaviours. Some just accepted HPV vaccination as '*something that needs to be done'* and felt reassured that it was recommended by the NHS: *"I think because it's like by the NHS - it kind of gives it validation." (Adolescent 9 - Yes)*. Others wanted the HPV vaccine leaflet to include more information about HPV and related health risks and vaccine side-effects, so that they did not panic if they experienced any of these.

In the FGD students expressed a preference for paper leaflets and discussed how the e-consent could bypass them: *"…because like if it's emailed, like your mum doesn't have to share it with you. And like if I have something done like an injection, I'd like to know what's going on and when. But like she filled out the form without like telling me, so like if they'd been given out in school then I could have read it and see what's happening."*

These FGD participants wanted to have the opportunity to discuss vaccination with their parents; they were not seeking autonomy in decision-making rather wanted a degree of joint responsibility.

"I wouldn't like to be given the option to like not to have the injection done…so I'm kind of glad that my mum just decided like on her own. But I would have liked her to talk it through with me…" (FGD participant)

Parents approaches to talking to their daughters about having an HPV vaccination are summarised in Text Box 2.

For peer review only

For peer review only - http://bmjopen.bmj.com/site/about/guidelines.xhtml

Text Box 2: Parents approaches to discussing HPV vaccination with adolescent daughters

Very limited discussion

- Did not talk to daughter in detail since vaccination is a parental decision;
- Talking too much about pending vaccination may induce anxiety, especially if needle phobic.

Heads up as what to expect

- Ensured daughter knew what to expect; this exchange usually occurred shortly before the immunisation session;
- Offered to answer any questions depending on adolescents' desire to know more.

More in-depth discussions

- Joint decision-making between parents and adolescents about the important of vaccination, this sometimes involved accessing additional information.

Using the e-consent intervention

The parents we interviewed found the system easy to use and usually completed the form as soon as they received it. A few parents would have liked an email confirmation after they had completed the e-consent form.

"I thought it was very easy. I think you're probably going to get more responses that way from parents in this day and age. However, the downside is obviously you may not get that chance to discuss it. (Parent 5 – Yes)

According to feedback from nurses and schools not all parents found the intervention easy to access or use. Language barriers accounted for some difficulties, but practical issues also played a role, for example some parents had not signed up for the school parent mail system hence did not receive the weblink. Nurses also received a significant number of calls from parents reporting that the weblink would not open or that webpages froze. During an immunisation session one student stated: *"my dad said I should have the vaccine, but he did not understand the whole google business about it".* In some instances, the weblink closed a few days prior to an immunisation session to give immunisation teams time to screen student information prior to sessions. This resulted in some

For peer review only - http://bmjopen.bmj.com/site/about/guidelines.xhtml

parents who had missed the last sign-up date sending in written notes to confirm their consent/non-consent for vaccination.

Outputs

Nurses access to e-consent forms and student information

The immunisation sessions at e-consent schools were affected by nurses not being able to access electronic information about students before and during sessions. The lower return of consent forms in e-consent schools also resulted in nurses reporting that they had to contact more parents than usual during immunisation sessions to obtain verbal consent. This had implications for the nurses' workload distribution and the length of sessions. To manage these challenges immunisation teams increased the number of nurses and administrative assistants who attended e-consent school immunisation sessions.

"…we had 80 consent forms outstanding at a big school. But, normally, if you only have a couple it's fine. It meant us was making calls all morning, it took a nurse out of immunising to be able to do that, so that did have a big impact." (Immunisation Team 2)

Conducting phone calls during sessions was not straightforward. Firstly, nurses had to rely on students (if they had phones with them) or staff to help them access correct contact details. Secondly, immunisations sessions were busy and noisy which impeded communication and privacy. Thirdly, it was not always possible to reach parents who were at work or out of the house during day-time hours. If parents were uncontactable the nurses assessed if students who wanted to be vaccinated had sufficient maturity and intelligence to understand and appraise the nature and implications of the proposed vaccination[12, 13]. This process was time-consuming and not all nurses felt comfortable about vaccinating without verbal or written parent permission.

For peer review only - http://bmjopen.bmj.com/site/about/guidelines.xhtml

Transition: adapting to change and iterative development

The initial 'buzz' about the e-consent intervention decreased over time. Whilst some staff remained positive and receptive to the implementation coordinators enthusiasm and vision, others expressed a sense of half-heartedness about having to adapt quickly from a known way of obtaining consent, albeit with flaws (e.g. cost of paper, mileage clocked up in collecting paper consent forms from schools), to a new technology enabled way with some functional limitations in Year 1 (see Figure 1).

In the HRCH staff group interviews conducted in July 2019 members of the immunisation teams reflected on lessons learnt from their experience of transitioning to a *'brand new way of working'* over the past year. Key learning points from an internal organisational perspective were: (i) adopt right pace of progress when introducing new interventions with have several components, (ii) be clear about which part of a multi-component intervention is being piloted and implemented (e.g. in Year 1 it was primarily about the e-consent form), (iii) importance of timely communication, quick thinking, and flexibility when things do not quite go to plan.

In terms of school engagement HRCH staff emphasised the importance of close collaboration to devise appropriate means of consent in different educational and social contexts. The right balance of responsibility between schools and immunisation teams needs to be negotiated to maintain positive working relationships and ensure that adolescents can access essential vaccines.

"I would also say the idea of just changing to e-consent… schools need different things… it is really important to work with the school and a make sure that they are happy with everything and it suits that school, because some schools it might just not suit right now. It might suit them in a couple of years, but right now it just doesn't work." (HRCH immunisers group interview, July 2019)

HRCH made several changes to the implementation of the e-consent intervention in Year 2 (2018/19) informed by evaluation findings (Text Box 3).

For peer review only - http://bmjopen.bmj.com/site/about/guidelines.xhtml

Text Box 3:
E-consent intervention implementation changes between Yr1 and Yr2

- ❖ Taking more time to engage (emails, phone calls and meetings) with schools in preparations to find the right level of involvement
- ❖ Ensuring students receive a paper copy of the HPV adolescent programme leaflet produced by Public Health England in addition to information provided in the e-consent form
- ❖ Pushing more for assemblies and contact with adolescent girls prior to the immunisation sessions
- ❖ Providing ongoing training and mentoring of immunisation teams on use of the intervention

For peer review only

Discussion

The pilot e-consent intervention had no detected impact on vaccine uptake and did not improve the return rate of consent forms in its first year of implementation. This could be interpreted as a negative result, however this would fail to account for the embedding process required before new technologies become (or do not become) routinely incorporated into everyday practice[14], and inherent difficulties in achieving high vaccine uptake in urban centres. Our findings demonstrate that the transition from paper to e-consent was not straightforward, and that staff and schools, though mainly open to change, took time to adapt. The transition was hampered by the reduced functionality of the IT capability (intervention fidelity), limited staff training and engagement of schools. It is likely that this e-consent intervention would need to be used for more than one year to achieve any benefits to process efficiency and vaccine uptake.

This study starts to address an evidence gap and demonstrates how real-time evaluation can support the iterative development of interventions essential for their longer-term integration. Key limitations include the lack of interviews with school staff to complement the feedback forms and not being able to access and interview parents who found the intervention more challenging to use.

The 'Normalisation Process Theory' (NPT) argues that new practices become routinely embedded as the result of people working, individually and collectively, to enact them. Enactment is promoted or

For peer review only - http://bmjopen.bmj.com/site/about/guidelines.xhtml

inhibited through the operation of four mechanisms; coherence (sense-making), cognitive participation (individual buy-in), collective action (joint effort) and reflexive monitoring (user appraisal)[15]. Sense-making requires users to have a shared understanding concerning the purpose of an intervention, their responsibilities in its implementation, its potential benefits and the differences to existing practice[16]. In our study, immunisation teams recognised the potential value of the intervention but were not clear about their specific responsibilities and schools had to make sense of the change in their role in consent logistics. User buy-in is defined as user's agreement to try out a new way of completing a task and their willingness to drive and sustain the implementation of a new intervention[15]. HRCH coordinators encouragement, ability to adapt when problems arose was critical to sustaining momentum throughout the implementation phase. Most schools bought into the concept of technology-enabled consent yet their willingness to sustain this change could have benefited from earlier collaboration between immunisation teams, head teachers, and link staff. Parents and student's engagement with the e-consent intervention was variable. Parent interviewees accessed the online portal and were able to complete and submit the e-consent form with ease. However, the significant difference in consent form return rate between e-consent and paper consent schools (73.3% (n=11) vs 91.6% (n=11), p=0.008) response rate (73.3%) indicated that this was not the case for all parents. Of additional concern was that the e-consent intervention inadvertently bypassed some adolescents' information needs and related opportunity to talk to their parents about HPV vaccination. Collective action was evidenced when immunisation teams and schools worked together to make the follow-up of e-consent form non-response more targeted. Reflexive monitoring was facilitated by this real-time evaluation although more co-production activities with HRCH staff, parents, school link staff and adolescents need to be integrated in subsequent evaluations[17, 18].

There is a need to streamline consent processes for adolescent immunisation and ensure that parents and adolescents are fully informed about these preventative measures. Electronic consent

For peer review only - http://bmjopen.bmj.com/site/about/guidelines.xhtml

could be an effective option to achieve this. However, it needs to be tailored to specific contexts, and parents, schools and adolescents need to provide input in the development, implementation and evaluation of such technological interventions. Evaluations also need to factor in the time needed for new working practices to be fully integrated. Our experience suggests that to gain a complete and accurate assessment of the impact of new interventions evaluations need to collect data over more than one cycle of implementation. Introducing change that affects different actors requires all stakeholders to understand, buy in and work together in refining and co-producing complex behavioural interventions.

For peer review only

Acknowledgements

We are grateful for the information shared freely by interview participants, schools and the Hounslow and Richmond Community Healthcare NHS Trust.

Author Contributors

TC and SMJ designed the study with input from SR, EE, HN, ME and RC. EP led the quantitative data analysis with support from RC and SB. TC collected and led the analysis of the qualitative data with support from SB. All authors discussed the preliminary findings and contributed to and critically reviewed the manuscript.

Funding

The research was funded by the National Institute for Health Research Health Protection Research Unit (NIHR HPRU) in Immunisation (Grant reference: HPRU-2012-10096) at the London School of Hygiene and Tropical Medicine in partnership with Public Health England (PHE). The views expressed are those of the author(s) and not necessarily those of the NHS, the NIHR, the Department of Health or Public Health England.

For peer review only - http://bmjopen.bmj.com/site/about/guidelines.xhtml

Competing interests

Tracey Chantler, Sadie Bell, and Sandra Mounier-Jack report that they were in receipt of funding from the National Institute of Health Research while conducting this research. Michael Edelstein, Rosie Cooper and Ellen Pringle worked for Public Health England for the duration of this research.

Ethics approval

The research published in this manuscript was approved by the PHE Research Support & Governance Office (Ref: NR0131) and the London School of Hygiene and Tropical Medicine Observational/Interventions Research Ethics Committee (Ref: 15839). Participants who took part in the qualitative component of this research received study information letters and were provided the opportunity to ask questions before they agreed to take part. Written informed consent was obtained from all research participants prior to interviews (individual, peer-group, parent adolescent dyad) and the FGD. For the latter parental and adolescent informed consent was obtained and school or immunisation staff were not present during the discussion. All participants were told that their participation was voluntary, and their data was stored and presented in a manner that seeks to protect their confidentiality.

Permission for publication

Not applicable

Data availability statement

Data collection tools are provided as supplementary documents and some anonymised data is available on request via the LSHTM public repository.

For peer review only - http://bmjopen.bmj.com/site/about/guidelines.xhtml

References

1. Lu, B., et al., *Efficacy and safety of prophylactic vaccines against cervical HPV infection and diseases among women: a systematic review & meta-analysis.* BMC infectious diseases, 2011. **11**: p. 13-13.
2. Gellin, B., et al., *Quadrivalent Human Papillomavirus Vaccine.* Clinical Infectious Diseases, 2007. **45**(5): p. 609-617.
3. Paul, P. and A. Fabio, *Literature review of HPV vaccine delivery strategies: Considerations for school- and non-school based immunization program.* Vaccine, 2014. **32**(3): p. 320-326.
4. Paterson, P., et al., *Strengthening HPV vaccination delivery: findings from a qualitative service evaluation of the adolescent girls' HPV vaccination programme in England.* Journal of Public Health, 2019.
5. Bentley C, *Tackling gaps and inequalities in girls' uptake of the current HPV vaccination programme. Available at: http://emhf.org/wp-content/ uploads/2015/10/EMHF-HPV-Symposium-April-2015- REPORT.final_.pdf (last accessed 29 June 2016).* 2015.
6. Ferrer, H.B., et al., *Barriers and facilitators to HPV vaccination of young women in high-income countries: a qualitative systematic review and evidence synthesis.* BMC public health, 2014. **14**: p. 700-700.
7. Batista Ferrer, H., et al., *Barriers and facilitators to uptake of the school-based HPV vaccination programme in an ethnically diverse group of young women.* Journal of public health (Oxford, England), 2016. **38**(3): p. 569-577.
8. Boyce, T. and A. Holmes, *Addressing Health Inequalities in the Delivery of the Human Papillomavirus Vaccination Programme: Examining the Role of the School Nurse.* PLOS ONE, 2012. **7**(9): p. e43416.
9. Brabin, L., et al., *The school nurse, the school and HPV vaccination: A qualitative study of factors affecting HPV vaccine uptake.* Vaccine, 2011. **29**(17): p. 3192-3196.
10. National Information Board, *Personalised Heatlh and Care 2020. Using Data and Technology to Transform Outcomes for Patients and Citizens: A Framework for Action*. 2014, HM Government, NHS: file:///C:/Users/sdbltcha/Downloads/national_information_board_s_personalised_health_and_care_2020_framework.pdf (accessed 16th Jan 2020).
11. Boyatzis, R., *Transforming qualitative information: Thematic analysis and code development*. 1998, Thousand Oaks, CA: Sage
12. Griffith, R., *What is Gillick competence?* Human vaccines & immunotherapeutics, 2016. **12**(1): p. 244-247.
13. Public Health England, *Consent: the green book, chapter 2*, in *The Green Book: Immunisation against infectious disease*. 2013, Public Health England: https://assets.publishing.service.gov.uk/government/uploads/system/uploads/attachment_data/file/144250/Green-Book-Chapter-2-Consent-PDF-77K.pdf (accessed 22nd October 2018).
14. May, C.R., et al., *Development of a theory of implementation and integration: Normalization Process Theory.* Implementation science : IS, 2009. **4**: p. 29-29.
15. Finch, T.L., et al., *Improving the normalization of complex interventions: measure development based on normalization process theory (NoMAD): study protocol.* Implementation Science, 2013. **8**(1): p. 43.
16. Mair, F.S., et al., *Factors that promote or inhibit the implementation of e-health systems: an explanatory systematic review.* Bulletin of the World Health Organization, 2012. **90**(5): p. 357-364.
17. Farr, M., et al., *Implementing online consultations in primary care: a mixed-method evaluation extending normalisation process theory through service co-production.* BMJ Open, 2018. **8**(3): p. e019966.

For peer review only

For peer review only - http://bmjopen.bmj.com/site/about/guidelines.xhtml

18. Osborne, S.P., Z. Radnor, and K. Strokosch, *Co-Production and the Co-Creation of Value in Public Services: A suitable case for treatment?* Public Management Review, 2016. **18**(5): p. 639-653.

Figures Legends

Figure 1: The Electronic consent intervention

Figure 2: Theory of Change for the e-consent intervention

---

[i] *Paper consent would have been used in the 14 control schools at school and community based catch up clinics.*

All parents in e-consent schools would have received the weblink to the e-consent portal and in most cases will have used this link to complete the consent form. Some students who attended e-consent school catch up clinics did not have a signed e-consent form. In those instances, the nurses would phone parents during clinics or obtain adolescent self-consent if appropriate. In the case of community-based catch up clinics parents/guardians and students would usually attend together, if the e-consent had not been completed nurses would either complete the e-consent form at the clinic with the parent and student, if Wifi/technology was available or complete a paper consent form.

For peer review only

Figure 1: The Electronic consent intervention

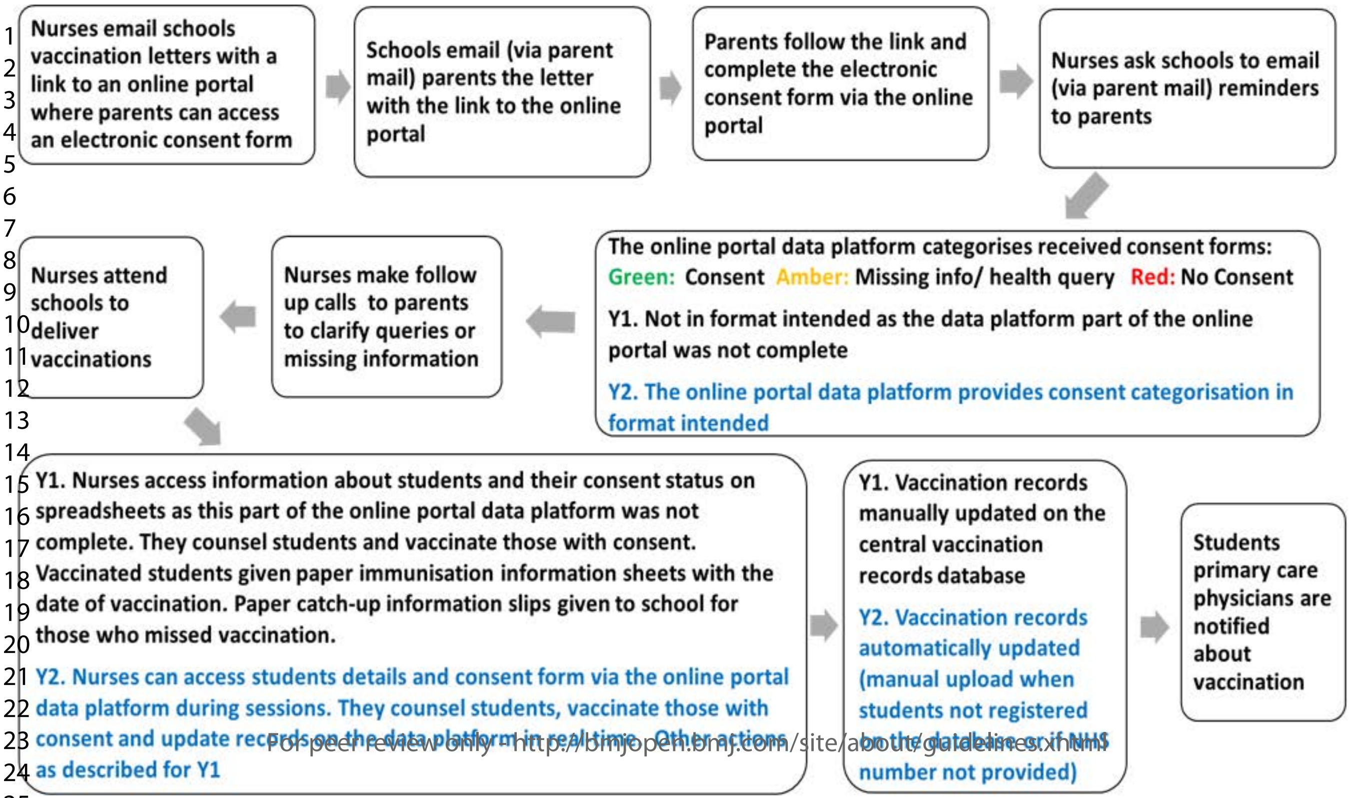

For peer review only - http://bmjopen.bmj.com/site/about/guidelines.xhtml

Figure 2: Theory of Change for the e-consent intervention

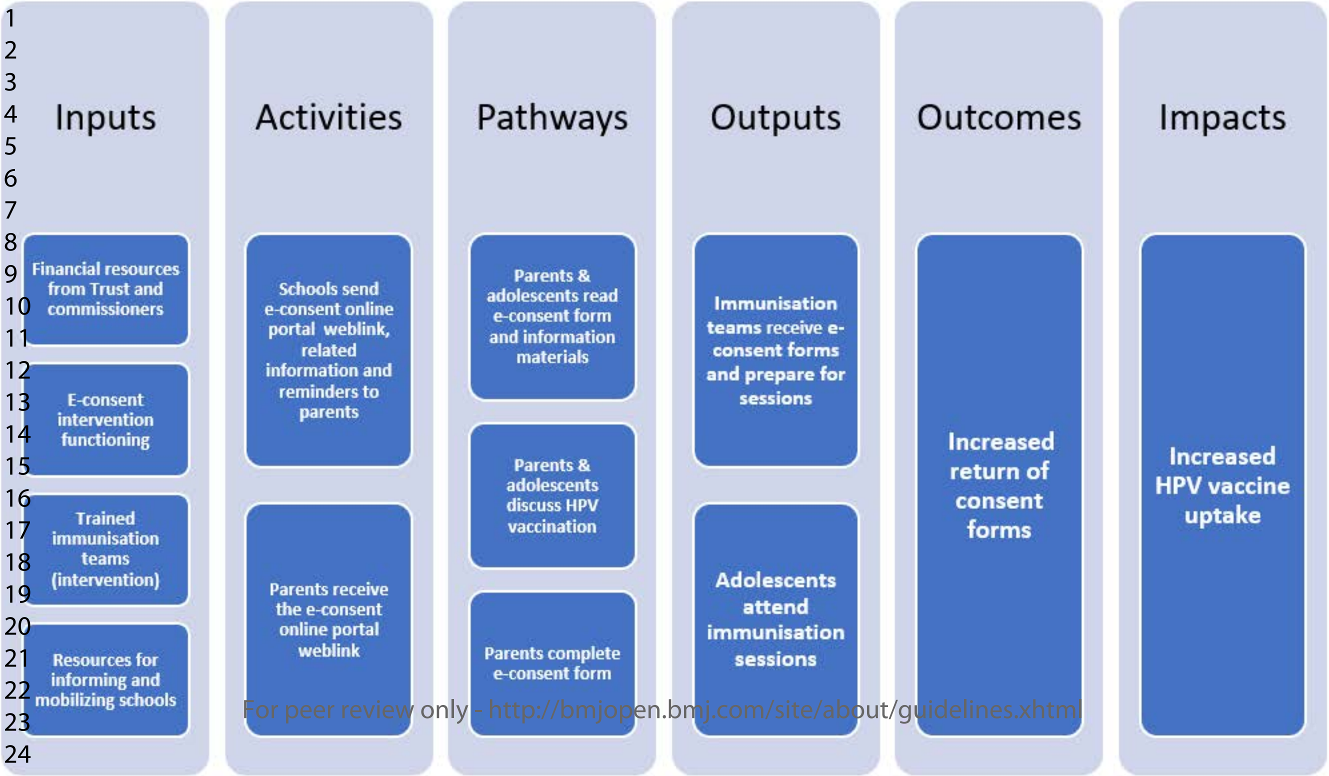

For peer review only - http://bmjopen.bmj.com/site/about/guidelines.xhtml

Additional File 1: School evaluation forms

DOES ELECTRONIC CONSENT IMPROVE THE LOGISTICS AND UPTAKE OF HPV VACCINATION IN ADOLESCENT GIRLS? A MIXED METHODS THEORY INFORMED EVALUATION OF AN INTERVENTION

The research published in this manuscript was approved by the PHE Research Support & Governance Office (Ref: NR0131) and the London School of Hygiene and Tropical Medicine Observational/Interventions Research Ethics Committee (Ref: 15839).

For peer review only

School evaluation form for e-consent schools

Introduction

The Hounslow and Richmond Community Health Trust is in the process of introducing a system that facilitates the collection of electronic consent for school-aged vaccinations. Your school has been involved in using the e-consent system as part of the HPV vaccination programme and we are keen to learn from this experience. We would be very grateful if you could complete this anonymous evaluation form and return it in the attached envelope to a member of the immunisation team at the end of the immunisation session today.

The information that you share with us will help us to evaluate the usability and acceptability of the e-consent system for school-based immunisations and assess its effectiveness in promoting consent form return and HPV vaccine uptake. The findings will guide decision-making about the future use of the e-consent system and help improve the system.

If you have any questions about this please do not hesitate to contact the research team who are based at the London School of Hygiene & Tropical Medicine.

Contacts:

Dr Tracey Chantler
LSHTM, 15-17 Tavistock Place
London, WC1H 9SH
tracey.chantler@lshtm.ac.uk

Sandra Mounier-Jack
LSHTM, 15-17 Tavistock Place
London, WC1H 9SH
sandra.mounier-jack@lshtm.ac.uk

Evaluation form

Part 1

On a scale of 1-10 (1 being poor, 10 being excellent) please rate the following by circling your number of choice. Please add comment to explain your ratings.

a) The organisation of the immunisation session

1	2	3	4	5	6	7	8	9	10
Poor				Average				Excellent	

Comments:

____
____
____
____

b) The usability of the e-consent system for obtaining consent for adolescent vaccination.

1	2	3	4	5	6	7	8	9	10
Poor				Average				Excellent	

Comments:

____
____
____
____

c) The efficiency of the e-consent system (in terms of reducing administrative burden and improving the organisation of immunisation sessions)

1	2	3	4	5	6	7	8	9	10
Poor				Average				Excellent	

Comments:

____
____
____
____

d) Communication between the immunisation team and the school

1	2	3	4	5	6	7	8	9	10
Poor				Average				Excellent	

Comments:

____
____
____
____

For peer review only

Part 2

a) What role have your school staff played in preparing for the HPV immunisation session?

1. Educational activities (*please tick*): Yes ☐ No ☐

If yes please state what these were (e.g. assembly):

____
____
____
____

2. Providing list of eligible students (*please tick*) Yes ☐ No ☐

3. Send reminders to parents (tick all relevant methods):

   a. Parent Mail ☐
   b. Post reminders to home address ☐
   c. Handing out reminders students ☐
   d. Text messages to parents/guardians ☐
   e. Phone call to parents/guardians ☐
   f. Email to parent/guardians with consent form link ☐

4. How many reminders did you send to non-respondents: No: ______________

5. Have you helped parents/adolescents access computers to be able to fill in the form online?

Yes ☐ No ☐

b) What is your experience of using or facilitating use of the e-consent system? What do you think about obtaining consent for school-based teenage vaccination electronically?

____
____
____
____

c) What have parents and students said about the e-consent system?

____
____
____
____

For peer review only

d) Which means of obtaining consent do you think is more efficient: paper based or e-consent?

____

e) How do you think the system could be improved?

____

Thank you for completing this evaluation form

For peer review only

School evaluation form for paper-based consent schools

Introduction

The Hounslow and Richmond Community Health Trust is in the process of introducing a system that facilitates the collection of electronic consent for school-aged vaccinations. Your school is likely to use this system in the future hence we are keen to learn from your current experience of being involved in coordinating the HPV vaccination programme for adolescent girls. We would be very grateful if you could complete this anonymous evaluation form and return it in the attached envelope to a member of the immunisation team at the end of the immunisation session today.

The information that you share with us will help us to evaluate the usability and acceptability of the e-consent system for school-based immunisations and assess its effectiveness in promoting consent form return and HPV vaccine uptake. The findings will guide decision-making about the future use of the e-consent system and help improve the system.

If you have any questions about this please do not hesitate to contact the research team who are based at the London School of Hygiene & Tropical Medicine.

Contacts:

Dr Tracey Chantler
LSHTM, 15-17 Tavistock Place
London, WC1H 9SH
tracey.chantler@lshtm.ac.uk
07969404745

Sandra Mounier-Jack
LSHTM, 15-17 Tavistock Place
London, WC1H 9SH
sandra.mounier-jack@lshtm.ac.uk
020 7927 2929

Rosie Cooper
Address to be added

rosie.cooper@phe.gov.uk

For peer review only

Evaluation form

Part 1

On a scale of 1-10 (1 being poor, 10 being excellent) please rate the following by circling your number of choice. Please add comments to explain your ratings.

e) The organisation of the immunisation session

1	2	3	4	5	6	7	8	9	10
Poor				Average				Excellent	

Comments:

____
____
____
____

f) The usability of the current paper-based system for obtaining consent for adolescent vaccination.

1	2	3	4	5	6	7	8	9	10
Poor				Average				Excellent	

Comments:

____
____
____
____

g) The efficiency of the paper-based system (in terms of administrative burden and organisation of immunisation sessions)

1	2	3	4	5	6	7	8	9	10
Poor				Average				Excellent	

Comments:

____
____
____
____

h) Communication between the immunisation team and the school

1	2	3	4	5	6	7	8	9	10
Poor				Average				Excellent	

Comments:

____
____
____
____
____

For peer review only

Part 2

a) What role have your school staff played in preparing for the HPV immunisation session?

1. Educational activities (*please tick*): Yes ☐ No ☐

   If yes please state what these were (e.g. assembly):

   ____
   ____
   ____
   ____

2. Providing list of eligible students (*please tick*) Yes ☐ No ☐

3. Send reminders/consent forms to parents (tick all relevant methods):

   a. Parent Mail ☐

   b. Post reminders/consent forms to home address ☐

   c. Handing out reminders/consent forms to students ☐

   d. Text messages to parents/guardians ☐

   e. Phone call to parents/guardians ☐

   f. Email to parent/guardians with consent form attached ☐

4. How many reminders did you send to non-respondents: No: ______________

b) What is your experience of using or facilitating use of the existing consent system?

____
____
____
____
____

c) What do you think about obtaining consent for school-based teenage vaccination electronically?

____
____
____
____
____

Thank you for completing this evaluation form

For peer review only

Additional File 2: Interview Topic Guides

DOES ELECTRONIC CONSENT IMPROVE THE LOGISTICS AND UPTAKE OF HPV VACCINATION IN ADOLESCENT GIRLS? A MIXED METHODS THEORY INFORMED EVALUATION OF AN INTERVENTION

The research published in this manuscript was approved by the PHE Research Support & Governance Office (Ref: NR0131) and the London School of Hygiene and Tropical Medicine Observational/Interventions Research Ethics Committee (Ref: 15839).

Interviewees: Immunisation team members

For peer review only

Experience of obtaining consent in school-based vaccination programmes

- Could you tell me about your experience of obtaining consent from parents and adolescents for school-aged vaccinations?
    - What has worked well and what has been challenging?
    - How have you overcome challenges in the past?

Acceptability of the e-consent system

- What were your initial thoughts about the e-consent system? (Probes: concerns, hopes, expectations)
- What did your colleagues think about the system?
- How acceptable do you think the system is for parents, teenagers, and school staff involved in the programme?

Usability of the e-consent system

- How helpful was the training in preparing you to use the e-consent system?
- What was your experience of using the e-consent system? What worked well, what was difficult in the following stages?
    - Triage of consent forms
    - Use of the system during immunisation sessions in schools
    - Uploading data to Trust databases and Child Health Immunisation Systems
    - Any other stages
- What contact did you have with parents about the use of the e-consent system? How did parents find using the e-consent system? What did they find difficult?
- What do you think about how the system may support communication between parents/guardians and adolescents about immunisation? How does this compare with the paper-based system?
- How does the e-consent system support communication between immunisation teams, parents and students? How does this compare with the paper-based system?
- What are the advantages and disadvantaged of the e-consent system in comparison with the paper-based system?

Interaction between schools and immunisation teams

- What is your experience of working with schools in organising immunisation sessions?
- What were schools reactions to the introduction of the e-consent system?
- How has the e-consent system supported interactions between schools and immunisation teams? (Probes: what has worked well, what has been challenging?) How does this compare with the paper-based system?
- What have schools told you about their experience of using the e-consent system? How do they compare it with the paper-based system?
- Have schools conducted any educational activities in preparation for the HPV immunisation visits? What were these? How have you been involved in these?

Adolescent self-consent

- What do you think about giving teen-agers the opportunity to self-consent for vaccination? (Probes: age, what vaccines e.g. HPV)
- Have you obtained self-consent for adolescent immunisation? If yes, in what instances, what was the procedure, and how did you assess for competency?
- How does the e-consent system support self-consent? What is the procedure for this? Did any teen-agers use the e-consent system to give consent for HPV vaccination? What was that experience like?
- Have you had conversations with schools about adolescent self-consent? What are their views?
- Have you had conversations with parents/guardians about adolescent self-consent? What were their views?

Reflections of the use of the e-consent system

- What have been the main lessons in piloting the e-consent system (Probes: what has worked well, what needs to be changed?)
- How would you modify and improve the e-consent system based on the piloting experience?
- What is your advice for other immunisation providers thinking about implementing e-consent?
- Has the e-consent system achieved its intended outcomes from your perspective?

Any other comments

Do you have any other comments?

Thank you for your time.

For peer review only

Interviewees: Service managers

Experience of obtaining consent in school-based vaccination programmes

- Could you tell me about your past experience of managing the logistics of obtaining consent from parents and adolescents for school-aged vaccinations?
    - What has worked well and what has been challenging?
    - How have you overcome challenges in the past?

Decision to launch the e-consent system

- What were the main reasons you decided to implement the e-consent system?
- How did you decide who to collaborate with in developing the e-consent system?
- How was the process of development? What went well, what was difficult?
- How did your test the system prior to the piloting phase?

Acceptability of the e-consent system

- How did your team initially react to the plans to introduce the e-consent system? (Probes: Expectations, concerns, questions)
- Did opinion change over time, and if yes, how?
- How acceptable do you think the e-consent system is for nurses, parents, teenagers, and school staff involved in the programme?

Usability of the e-consent system

- How were you involved in using the e-consent system?
- What was your/your teams' experience of using the e-consent system? What worked well, what was difficult in the following stages?
    - Triage of consent forms
    - Use of the system during immunisation sessions in schools
    - Uploading data to Trust databases and Child Health Immunisation Systems
    - Any other stages
- What contact did you/your team have with parents about the use of the e-consent system? How did parents find using the e-consent system? What did they find difficult?
- What do you think about how the system may support communication between parents/guardians and adolescents about immunisation? How does this compare with the paper-based system?
- How does the e-consent system support communication between immunisation teams, parents and students? How does this compare with the paper-based system?
- What are the advantages and disadvantaged of the e-consent system in comparison with the paper-based system?

For peer review only

Interaction between schools and immunisation teams

- What is your experience of working with schools in organising immunisation sessions?
- What were schools reactions to the introduction of the e-consent system? How did you inform them about the e-consent system, what guidance or training did you provide?
- How has the e-consent system supported interactions between schools and immunisation teams? (Probes: what has worked well, what has been challenging?)
- How does this compare with schools that are using the paper system?
- What have schools told you about their experience of using the e-consent system? How do they compare it with the paper-based system?
- Have schools (those using e-consent and paper system) conducted any educational activities in preparation for the HPV immunisation visits? What were these? How have you/your team been involved in these?

Adolescent self-consent

- Please describe the process you have used for self-consent
- What do you think about giving teenagers the opportunity to self-consent for vaccination? (Probes: age, what vaccines e.g. HPV)
- Have you had conversations with schools about adolescent self-consent? What are their views?
- Have you had conversations with parents/guardians about adolescent self-consent? What were their views?

Reflections of the use of the e-consent system

- What have been the main lessons in piloting the e-consent system (Probes: what has worked well, what needs to be changed?)
- How will you modify and improve the e-consent system based on the piloting experience?
- What have been the costs of implementing the e-consent system?
- What is your advice for other immunisation providers thinking about implementing e-consent?
- Has the e-consent system achieved its intended outcomes from your perspective?

Any other comments

Do you have any other comments?

Thank you for your time.

For peer review only

Interviewees: Service administrators

Experience of managing the administration of the school-based vaccination programmes

- Could you tell me about your past experience of managing the administration of the school-aged vaccination programme?
    - What has worked well and what has been challenging?
    - How have you overcome challenges in the past?

Decision to launch the e-consent system (for senior administrators)

- What were the main reasons the Trust decided to implement the e-consent system?
- How was the process of development? What went well, what was difficult?
- How did your test the system prior to the piloting phase?

Acceptability of the e-consent system

- What were your initial thoughts about the plans to introduce the e-consent system? (Probes: Expectations, concerns, questions)
- Did your opinion change over time, and if yes, how?
- What is your current view on the acceptability of the e-consent system?

Usability of the e-consent system

- How are you involved in using the e-consent system?
- What was your/your teams' experience of using the e-consent system? What worked well, what was difficult in the following stages?
    - Administration of data from the consent forms
    - Administration of data from school and catch-up immunisation sessions
    - Uploading data to Trust databases and Child Health Immunisation Systems
    - Sharing data with GPs
    - Any other stages
- What are the advantages and disadvantaged of the e-consent system in comparison with the paper-based system?

Interaction between schools and immunisation teams

- What role do you play in collaborating with schools to organising immunisation sessions?
- What were schools reactions to the introduction of the e-consent system? How were they informed about the e-consent system, what guidance or training did they receive?
- How has the e-consent system supported interactions between schools and immunisation providers? (Probes: what has worked well, what has been challenging?)
- How does this compare with schools that are using the paper system?
- What have schools told you about their experience of using the e-consent system? How do they compare it with the paper-based system?

For peer review only

Reflections of the use of the e-consent system

- What have been the main lessons in piloting the e-consent system (Probes: what has worked well, what needs to be changed?)
- How will you modify and improve the e-consent system based on the piloting experience?
- What have been the costs of implementing the e-consent system?
- What is your advice for other immunisation providers thinking about implementing e-consent?
- Has the e-consent system achieved its intended outcomes from your perspective?

Any other comments

Do you have any other comments?

Thank you for your time.

For peer review only

Interviewees: Parents/guardians

Views on adolescent vaccination

- What do you know about the vaccines that are offered to teenagers? (Probes: HPV, teenage booster, Men ACWY)
- What information have you received about teenage vaccinations? Who gave you this information? Did you search for additional information, if yes where did you look and what did you find?
- What is your view about adolescent vaccination? Are there any vaccines that you are concerned about?

Experience of providing consent for adolescent vaccinations

- Could you tell me about your experience of providing consent for your teenager(s) to be vaccinated?
- How did you make your decision about providing consent?
    - Who else did you talk to about this? What did you discuss? What were their views? (Probes: Partner, teenager, other family members, friends, health professionals)
- How did you involve your teenager in this decision-making? Were you in agreement about having the vaccine?
- Have you ever changed your mind about providing consent for a vaccination, if yes why and what did you do?

Usability of the e-consent system

- How did you find using the e-consent system? Who completed the form?
- Was it easy to access the e-consent site via the email sent from school?
- What was you experience of completing the consent form online? Were there any parts that you found difficult to complete? How did you know if you had completed the form properly?
- What was your experience of accessing the HPV vaccination programme leaflets via the e-consent system? Did you read these online or did you print them out? Did you share these leaflets with your teenager or other members of the family?
- Did you contact the immunisation team for help to complete the e-consent form? What support did you require? How easy was it to contact them?
- How do you think that the e-consent form could be improved?

Acceptability of the e-consent system

- What are your views on giving consent electronically for your teenagers' vaccinations? (Probes: any concerns about sharing personal data online?)
- What did your teen-ager think about this method of giving consent?
- What are your views on how the e-consent system supports information sharing and communication about teenage vaccination?

For peer review only

Teenagers experience of immunisation in school

- What did your teenager tell you about their experience of being immunised in school? (Probes: what went well, what did they find difficult)
- How well prepared do you think your teenager was prior to the immunisation session? (Probes: access to information, educational sessions in school)

Views on adolescent self-consent

- What do you think about the option of teen-agers providing self-consent for vaccination?
- What is your understanding of self-consent in adolescence?
- Would you be happy for your teen-ager to give self-consent for vaccination? If yes, why. If no, why?

Any other comments

Do you have any other thoughts about adolescent vaccination and how consent is obtained for this?

Thank you for your time.

For peer review only

Interviewees: Adolescents (questions asked with parents)

Views on adolescent vaccination

- What do you know about the vaccines that are offered to teenagers? (Probes: HPV, teenage booster, Men ACWY)
- What information have you received about the HPV vaccination? Who gave you this information? Have you accessed any other information, if yes what was this?
- What is your view about the HPV vaccination? (Probes: what is good about it, any concerns about the vaccine or the process of vaccination)
- Who have you talked to about having the HPV vaccine? (Probes: parents, friends, school nurse, other health professionals, other family members) What did you discuss?

For peer review only

Experience of giving consent for vaccination

- Who decided whether you would receive the HPV vaccine? How did they make this decision?
- How were you involved in this decision-making? Were you in agreement with your parents about this decision?

Usability of the e-consent system

- Were you involved in using the e-consent system? If yes, how were you involved either at home or in school and what did you think about this system?
- What did your parents think about the e-consent system? Were there any parts of the online form that they found difficult to complete?
- Were you given the opportunity to find out more about the HPV vaccine via the e-consent system? (Probes: Access leaflets, read and discuss these with your parents)
- What do your friends think about the e-consent system?
- How do you think that the e-consent system could be improved?

Acceptability of the e-consent system

- What are your views on giving consent online for your vaccinations? (Probes: any concerns about sharing personal data?)
- What are your views on how the e-consent system supports finding out about and talking about teenage vaccination?

Experience of the immunisation session

- Could you tell me a bit about your experience of receiving the HPV vaccine in school? What was the session like, how did you feel, what went well, what was difficult?
- How well prepared were you for the immunisation session? (Probe: know you would be vaccinated against HPV, understood what the vaccine was for any why you needed it)
- Were you given the opportunity to ask any questions before you were vaccinated?
- Had you participated in any information sessions about vaccination at school before you were vaccinated? If yes, what were these and what did you learn?
- Had you received any other information about the HPV vaccine before you were vaccinated? If yes, who gave you this?

Views on adolescent self-consent

- What do you think about the option of teen-agers providing self-consent for vaccination?
- What is your understanding of self-consent?
- Have you ever been given the option to provide self-consent for vaccination?
- Would you be happy to provide self-consent for vaccination? If yes, why. If no, why?

Any other comments

Do you have any other thoughts about vaccination and how consent is obtained for this?

Thank you for your time

For peer review only

Adolescent Focus Group Discussion Topic Guide

Interviewees: Adolescents (Year 8/9 girls)

Views on adolescent vaccination

- What do you know about the vaccines that are offered to teenagers? (Probes: HPV, teenage booster, Men ACWY)
- What information have you received about the HPV vaccination? Who gave you this information? Have you accessed any other information, if yes what was this?
- What is your view about the HPV vaccination? (Probes: what is good about it, any concerns about the vaccine or the process of vaccination)
- Who have you talked to about having the HPV vaccine? (Probes: parents, friends, school nurse, other health professionals, other family members) What did you discuss?

Experience of giving consent for vaccination

- Who decided whether you would receive the HPV vaccine?
- How were you involved in this decision-making? Were you in agreement with your parents about this decision?

Usability of the e-consent system

- Were you involved in using the e-consent system? If yes, how were you involved either at home or in school and what did you think about this system?
- What did your parents think about the e-consent system? Were there any parts of the online form that they found difficult to complete?
- Were you given the opportunity to find out more about the HPV vaccine via the e-consent system? (Probes: Access leaflets, read and discuss these with your parents)
- What do your friends think about the e-consent system?
- How do you think that the e-consent system could be improved?

Acceptability of the e-consent system

- What are your views on giving consent online for your vaccinations? (Probes: any concerns about sharing personal data?)
- What did your parents think about this way of obtaining consent?

Experience of the immunisation session

- Could you tell me a bit about your experience of receiving the HPV vaccine in school? What was the session like, how did you feel, what went well, what was difficult?
- How well prepared were you for the immunisation session? (Probe: know you would be vaccinated against HPV, understood what the vaccine was for any why you needed it)
- Were you given the opportunity to ask any questions before you were vaccinated?

For peer review only

- Had you participated in any information sessions about vaccination at school before you were vaccinated? If yes, what were these and what did you learn?
- Had you received any other information about the HPV vaccine before you were vaccinated? If yes, who gave you this?

Views on adolescent self-consent

- What do you know about the option of teenagers providing self-consent for vaccination?
- Do you know what self-consent means?
- Would you be happy to provide self-consent for vaccination? If yes, why. If no, why?

Any other comments

Do you have any other thoughts about vaccination and how consent is obtained for this?

Thank you for your time.

For peer review only

Good Reporting of A Mixed Methods Study (GRAMMS)

The quality of mixed methods studies in health services research. Alicia O'Cathain, Elizabeth Murphy[1], Jon Nicholl Medical Care Research Unit, School of Health and Related Research, University of Sheffield, Sheffield; [1]School of Sociology and Social Policy, University of Nottingham, Nottingham, UK.

Accessed via: https://www.equator-network.org/reporting-guidelines/the-quality-of-mixed-methods-studies-in-health-services-research/

Applied to:

DOES ELECTRONIC CONSENT IMPROVE THE LOGISTICS AND UPTAKE OF HPV VACCINATION IN ADOLESCENT GIRLS? A MIXED METHODS THEORY INFORMED EVALUATION OF A PILOT INTERVENTION

(1) Describe the justification for using a mixed methods approach to the research question.

The use of a mixed methods theory-informed study design is described and justified in the strengths and limitations summary box (page 3, lines 60-62) and the study design section (page 5, lines 112-120). It is also illustrated in Figure 2. The study design allowed us to measure the effect of a pilot e-consent intervention on immunisation performance and identify mechanisms that facilitated or impeded implementation.

(2) Describe the design in terms of the purpose, priority and sequence of methods.

This is described in the study design section (page 5, lines 112-120); the choice of methods was informed by the need to obtain qualitative data to examine the implementation process and quantitative data to assess outcomes and the impact of the intervention.

(3) Describe each method in terms of sampling, data collection and analysis.

These are described in full in the methods section of the manuscript. The sampling frame for the schools participating in the evaluation is detailed in the selection of schools. The boundaries sampling of interviews participants for the qualitative methods were set by this selection. We interviewed all the staff who were actively involved in the implementation process and observed immunisation sessions at schools in each of the boroughs that piloted the intervention. The recruitment and sampling of parent and adolescent interviewees (individual and paired parent/adolescent) is outlined in lines 182-188. We were only able to recruit parents and by proxy adolescents who had ticked a box on the e-consent portal stating they were willing to be interviewed. Informed consent was obtained before all qualitative interviews as described in the ethics section at the end of the manuscript.

(4) Describe where integration has occurred, how it has occurred and who has participated in it.

For peer review only

The study team (co-authors) met every 3-4 months to discuss the results of the evaluation and compare quantitative with qualitative findings. The qualitative findings were pivotal for understanding why the pilot intervention did not achieve the expected improvement to consent form return in Year 1 and to identify mechanisms that facilitated or impeded the implementation of the intervention. Collaboration between implementers and evaluators in this real time evaluation also supported the iterative development of the pilot intervention, which is critical to the longer-term integration of new working practices.

(5) Describe any limitation of one method associated with the present of the other method.

I am not completely clear what is meant by this question. The methods complemented each other and were not limited by the presence of another method. The observations informed the interviews and findings from the qualitative data provided insights into implementation challenges which in part explained quantitative findings.

(6) Describe any insights gained from mixing or integrating methods.

As stated in point one the use of diverse methods allowed us to examine the implementation of the pilot intervention and assess its effect on consent form returns (outcomes) and HPV vaccine uptake (impact). Using qualitative methods over a period of a year was very useful to trace how the intervention was adapted over time to improve its acceptability and usability. The design of our evaluation could have been improved if we had been able to repeat the quantitative data collection in Year 2. This was not possible since the new iteration of the intervention was not ready when the original sample of e-consent schools were scheduled to receive the HPV vaccine in Year2. This is a constraint of evaluating interventions in real-time.

For peer review only
